# Developing a dynamic HIV transmission model for 6 U.S. cities: An evidence synthesis

**DOI:** 10.1371/journal.pone.0217559

**Published:** 2019-05-30

**Authors:** Emanuel Krebs, Benjamin Enns, Linwei Wang, Xiao Zang, Dimitra Panagiotoglou, Carlos Del Rio, Julia Dombrowski, Daniel J. Feaster, Matthew Golden, Reuben Granich, Brandon Marshall, Shruti H. Mehta, Lisa Metsch, Bruce R. Schackman, Steffanie A. Strathdee, Bohdan Nosyk, on behalf of the localized HIV modeling study group

**Affiliations:** 1 Health Economic Research Unit at the British Columbia Centre for Excellence in HIV/AIDS, Vancouver, BC, Canada; 2 Faculty of Health Sciences, Simon Fraser University, Burnaby, BC, Canada; 3 Hubert Department of Global Health, Emory Center for AIDS Research, Rollins School of Public Health, Emory University, Atlanta, GA, United States of America; 4 Department of Medicine, Division of Allergy & Infectious Disease, adjunct in Epidemiology, University of Washington, Seattle, WA, United States of America; 5 Center for Family Studies, Department of Epidemiology and Public Health, Leonard M. Miller School of Medicine, University of Miami, Miami, FL, United States of America; 6 International Association of Providers of AIDS Care, Washington, DC, United States of America; 7 Department of Epidemiology, Brown School of Public Health, Providence, RI, United States of America; 8 Bloomberg School of Public Health, Johns Hopkins University, Baltimore, MD, United States of America; 9 Department of Sociomedical Sciences, Mailman School of Public Health, Columbia University, New York, NY, United States of America; 10 Department of Healthcare Policy and Research, Weill Cornell Medical College, New York, NY, United States of America; 11 School of Medicine, University of California San Diego, La Jolla, CA, United States of America; Western University, CANADA

## Abstract

**Background:**

Dynamic HIV transmission models can provide evidence-based guidance on optimal combination implementation strategies to treat and prevent HIV/AIDS. However, these models can be extremely data intensive, and the availability of good-quality data characterizing regional microepidemics varies substantially within and across countries. We aim to provide a comprehensive and transparent description of an evidence synthesis process and reporting framework employed to populate and calibrate a dynamic, compartmental HIV transmission model for six US cities.

**Methods:**

We executed a mixed-method evidence synthesis strategy to populate model parameters in six categories: (i) initial HIV-negative and HIV-infected populations; (ii) parameters used to calculate the probability of HIV transmission; (iii) screening, diagnosis, treatment and HIV disease progression; (iv) HIV prevention programs; (v) the costs of medical care; and (vi) health utility weights for each stage of HIV disease progression. We identified parameters that required city-specific data and stratification by gender, risk group and race/ethnicity *a priori* and sought out databases for primary analysis to augment our evidence synthesis. We ranked the quality of each parameter using context- and domain-specific criteria and verified sources and assumptions with our scientific advisory committee.

**Findings:**

To inform the 1,667 parameters needed to populate our model, we synthesized evidence from 59 peer-reviewed publications and 24 public health and surveillance reports and executed primary analyses using 11 data sets. Of these 1,667 parameters, 1,517 (91%) were city-specific and 150 (9%) were common for all cities. Notably, 1,074 (64%), 201 (12%) and 312 (19%) parameters corresponded to categories (i), (ii) and (iii), respectively. Parameters ranked as best- to moderate-quality evidence comprised 39% of the common parameters and ranged from 56%-60% across cities for the city-specific parameters. We identified variation in parameter values across cities as well as within cities across risk and race/ethnic groups.

**Conclusions:**

Better integration of modelling in decision making can be achieved by systematically reporting on the evidence synthesis process that is used to populate models, and by explicitly assessing the quality of data entered into the model. The effective communication of this process can help prioritize data collection of the most informative components of local HIV prevention and care services in order to reduce decision uncertainty and strengthen model conclusions.

## Introduction

In the United States, more than 1.1 million people were estimated to be living with HIV in 2015, including 162,500 (15%) people who had not been diagnosed [1]. Although the number of people living with HIV is increasing and access to antiretroviral medications is extending life expectancy [2], current political uncertainty related to health financing is straining resources and challenging public health departments to use available funding efficiently [3]. Further complicating these decisions is the fact that HIV epidemics tend to be heterogeneous across geographic regions [4–6]. In the United States, the majority of people living with HIV/AIDS (PLHIV) reside in large urban centers that have unique underlying epidemiological and structural features [7]. This heterogeneity across regional microepidemics necessitates prioritizing resources according to the greatest public health benefit, accounting for the local epidemiological and structural context [6, 8, 9].

Increasingly, mathematical models are being used to help set priorities to address HIV microepidemics internationally [10–13]. Dynamic HIV transmission models can estimate, within a causal framework, all relevant costs and benefits attributable to HIV care interventions over an extended time horizon [14]. Such models can be adapted for multiple settings, capturing the heterogeneity across settings and also estimating the potentially synergistic effects of combinations of public health interventions to treat and prevent HIV [15]. However, these models are often data intensive because they require context-specific information about the demographics of HIV-negative and infected populations, heterogeneous HIV risk behaviors and access to health services such as HIV testing and antiretroviral treatment (ART), among other factors. While efforts to collect and compile population-based health administrative and surveillance data are rapidly increasing, the availability of representative, high-quality data still varies substantially within and across countries [16–18].

Comprehensively reporting the evidence synthesis process and sources of data used in a model can help readers assess its validity and the credibility of its inferences. In addition, calibrating a model to match a jurisdiction’s microepidemic over a stated period is a necessary condition for ensuring a model’s external validity [19]. Despite the importance of the quality of evidence entered into a model, there are no explicit guidelines for reporting the evidence synthesis process for models used in health economic evaluation [20–23]. While the efficiency and appropriateness of systematic searching for every model parameter has been questioned, it has been suggested that search approaches should reflect the complexity of the evidence [23, 24]. A recent modeling study for Vietnam [25] described the estimation techniques and triangulation methods that were used to approximate parameter values. The study drew on national surveillance data and behavioral surveys and provided a greater level of transparency in reporting on input data sources than had been seen elsewhere. Comprehensively and transparently reporting the evidence used in mathematical models improves reproducibility and allows it to be updated more easily as newer and higher-quality data become available. More importantly, this reporting process can reveal the areas of greatest uncertainty for sensitivity analysis, and, through value of information analysis [26], identify areas where additional surveillance data are worth collecting.

We aim to provide a comprehensive description of an evidence synthesis process and reporting framework that can be used to populate and calibrate a dynamic, compartmental HIV transmission model for six US cities. We hope to maximize the transparency of our model so that interested parties can review and evaluate its structure and equations, as well as the generating process and assumptions for all parameters (25), in order to promote the use of modeling recommendations in decision making to address city-level HIV microepidemics.

## Methods

### Model structure

We adapted a previously published dynamic, compartmental HIV transmission model [27–30] to simulate the HIV microepidemics in six US cities: Atlanta, Georgia; Baltimore, Maryland; Los Angeles, California; Miami, Florida; New York City, New York; and Seattle, Washington (boundaries defined in S1 Supplement). We selected these six cities because they represent nearly one-quarter of the US population of PLHIV [31] and because they have extensive epidemiological and structural differences in their public health responses to HIV [7]. For each city, the adult population aged 15–64 was stratified on the basis of gender (male or female), race/ethnicity (black/African American, Hispanic/Latino, and non-Hispanic white/others), and HIV risk behavior type (men who have sex with men (MSM), people who inject drugs (PWID), MSM who inject drugs (MWID), and heterosexual (HET)). MSM, MWID, and HET were further stratified into subgroups based on HIV sexual risk behavior intensity (high vs. low), and PWID and MWID were categorized based on whether they were receiving opioid agonist treatment (OAT).

**Fig 1:**
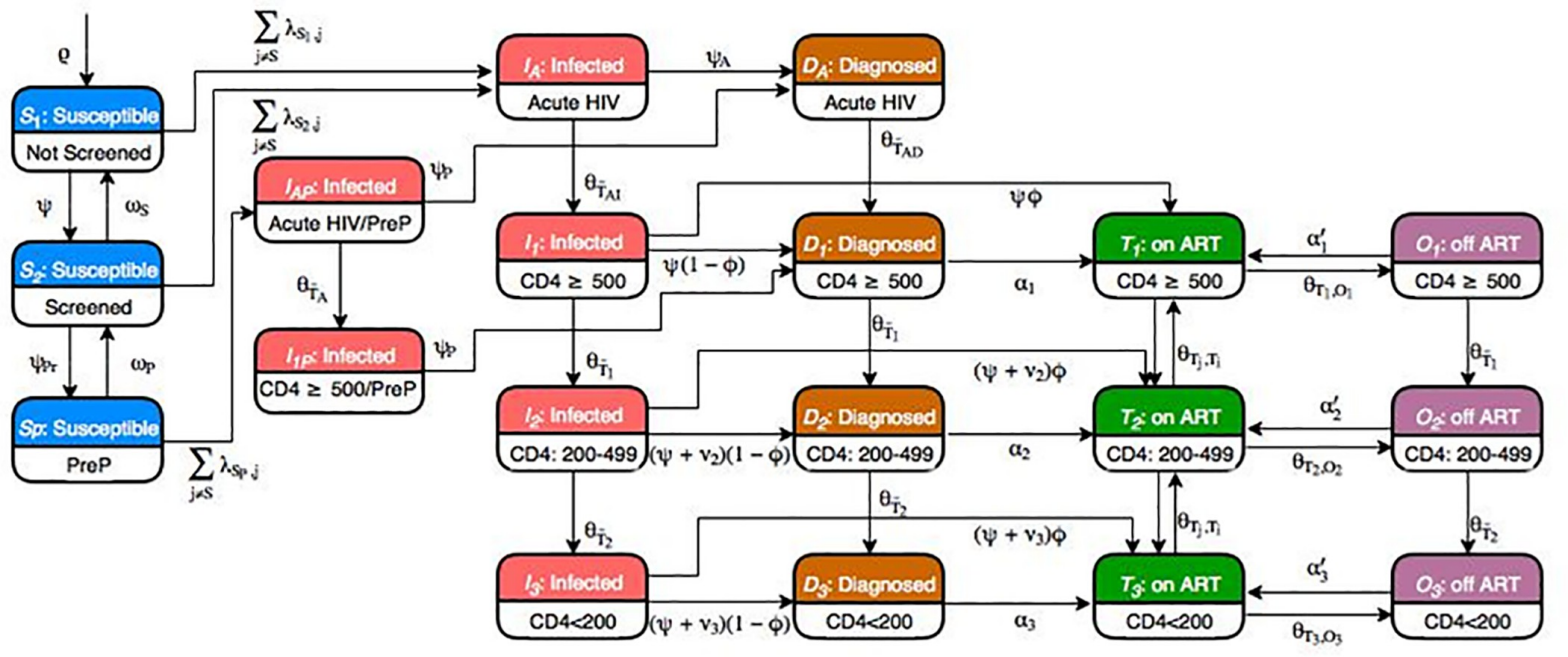
Dynamic compartmental HIV transmission model schematic diagram. For each city, the adult population aged 15–64 was stratified into compartments on the basis of (1) gender (male or female), (2) race/ethnicity (black/African American, Hispanic/Latino, and non-Hispanic white/others), and (3) HIV risk behavior type (men who have sex with men (MSM), people who inject drugs (PWID), MWID, and heterosexual (HET)). MSM, MWID, and HET were further stratified into subgroups based on HIV sexual risk behavior intensity (high vs low), and PWID and MWID were categorized based on whether they were receiving opioid agonist treatment (OAT). Individuals within each of these 42 strata (MSM: 6 groups, MWID: 12 groups; PWID: 12 groups; HET: 12 groups) progress through the model according to the 19 health states illustrated above. Prior to HIV infection, HIV-negative individuals can be screened for HIV (screened in past 12 months), and screened MSM or MWID can take pre-exposure prophylaxis (PrEP). HIV transmission can occur through three modes: heterosexual contact, homosexual contact, and needle-sharing. We specified the pattern of sexual mixing between risk groups and race/ethnicity, where assortativity determines the proportion of sexual contacts within the same group, and we varied the level of assortativity across cities (28). Following HIV infection, individuals transition through acute infection (3 months), then are classified as infected but not diagnosed, diagnosed but ART-naïve, and on- or off-ART, and partitioned according to CD4 cell count (CD4 ≥ 500, 200–499, and <200). Health state transitions occur at monthly intervals, with transition to death a possibility from each of the health states depicted, with varying probabilities.

Individuals within each of these 42 strata (MSM: 6 groups; MWID: 12 groups; PWID: 12 groups; HET: 12 groups) progress through the model according to the health states outlined in Fig 1. Prior to HIV infection, HIV-negative individuals can be screened for HIV (screened in the past 12 months), and screened MSM or MWID can take pre-exposure prophylaxis (PrEP). HIV transmission can occur through three modes: heterosexual contact, homosexual contact and needle sharing. We specified sexual mixing assortativity between risk groups and race/ethnicity to determine the proportion of sexual contacts within the same group, and varied the level of assortativity across cities [32, 33]. Following HIV infection, individuals transition through various stages beginning with acute infection (three months). They are then classified as infected but not diagnosed, diagnosed but ART-naïve, and on- or off-ART, and partitioned according to CD4 cell count (CD4 ≥ 500, 200–499, and <200). Health state transitions occur at monthly intervals and transition to death is a possibility from each of the health states depicted, with varying probabilities.

### Data requirements

We organized the data needed for the model into six model parameter categories: (i) initial HIV-negative and HIV-infected population estimates; (ii) parameters used to calculate the probability of HIV transmission; (iii) screening, diagnosis, treatment and HIV disease progression; (iv) HIV prevention programs, including syringe service programs (SSP), OAT, and PrEP; (v) the costs of medical care for HIV-negative and HIV-infected individuals; and (vi) health utility weights for each stage of HIV disease progression.

Each parameter in the model required a point estimate and range to facilitate model calibration and probabilistic sensitivity analysis according to best practice guidelines in model-based cost-effectiveness analysis [34, 35].

In addition to evidence informing model parameters, we required annual city-specific data for at least two time points to be used as calibration and validation targets for comparison of model projections [36, 37]. We chose the model calibration period (2012–2015) according to the availability of city-level surveillance data (stratified by gender, race/ethnicity, and risk group) for critical clinical and epidemiological endpoints characterizing the course of each HIV microepidemic [34].

### Evidence synthesis strategy

We first identified parameter estimates that we determined to be common across cities and generalizable across city-level microepidemics. The remaining parameters required context-specific data to adequately characterize the population mix, HIV risk behaviors and health care utilization patterns for a given city. We divided our search strategy into two parts: (i) identifying a rank order of a priori potential data sources for each model parameter category; and (ii) selecting the best data to use, given additional factors and constraints (S1 Supplement).

The best possible data source for each parameter depended on factors unique to each parameter category. For example, the most accurate and reliable source for total population numbers was city-level census data, while the best source for ART effectiveness estimates came from randomized controlled trials. For each non-city-specific (common) parameter, we selected source data based on study quality, how well a study matched the ideal study type for a given model parameter, and recency of the evidence. For city-specific parameters, we selected source data based on geographic representativeness and stratification level relative to our model requirements and recency of the evidence. We assessed recency according to evidence type as we required more up-to-date surveillance data for initial populations and calibration targets in comparison to other non-city-specific evidence such as efficacy data from RCTs or untreated HIV disease progression. When necessary, parameter estimates and ranges were derived from triangulation, defined as using numbers from multiple sources and/or from the same source but requiring additional assumptions to match our model’s level of stratification.

We used several search methods to identify evidence sources for the disparate data types, including searches in bibliographic databases (PubMed searches for (ii) parameters used to calculate the probability of HIV transmission conducted for all articles published prior to May 31, 2017; searches for (iii) Screening, diagnosis, treatment and HIV disease progression conducted for all articles published prior to February 8, 2018; searches for (iv) HIV Prevention Programs conducted for all articles published prior to October 26, 2018; and searches for (vi) Health utility weights conducted for all articles published prior to August 30, 2017), non-database searches, “snowballing” (such as searching references from key sources to identify further sources) (Google Scholar snowball searches for (ii) Parameters used to calculate the probability of HIV transmission conducted for all articles published prior to May 31, 2017), and local surveillance reports [23, 24, 38]. Where necessary due to a paucity of available published data, we sought out large and representative databases that could be used for primary analysis to further augment our evidence synthesis.

### Ranking data quality

The quality of each parameter was determined using context- and category-specific criteria, incorporating an adapted version of the Oxford Centre for Evidence-based Medicine–Levels of Evidence scale for common parameters (S1 Supplement) [39]. We ranked common parameter inputs according to the best quality of evidence that could be used to inform a given model parameter category. Best quality indicated a perfect match, moderate quality indicated that the evidence did not match perfectly or required some triangulation, and lowest-quality indicated that we derived parameter inputs by assumption or by another low quality evidence source.

For city-specific model parameters, we ranked the inputs according to how closely the evidence mapped onto the model stratification. Best quality indicated that the evidence data mapped onto the model parameter inputs by city perfectly (e.g., population-level data acquired since 2010 at the city level and stratified by risk group, gender, and race/ethnicity), moderate quality indicated that the evidence was stratified by city or region, with some level of population stratification or other triangulation, and lowest quality indicated that the evidence was at the national level, aggregated across population strata, or derived from expert opinion/assumption (e.g., aggregate data acquired prior to 2000 at the national-level).

All quality rankings were independently assessed by at least two team members and discrepancies were resolved through discussion and consensus among team members. Finally, missing city-level parameter values were assigned using a standardized algorithm to prioritize best-available data in surveillance and peer-reviewed literature at the (i) state, (ii) regional, or (iii) national level.

### Data verification

Where the available data was less than ideal in at least two ways (e.g., potentially outdated according to the parameter category, geographically non-specific, or lacking stratification by gender, risk group or race/ethnicity), we posed explicit questions to our scientific advisory committee, a collection of city-specific experts, to confirm use of the best-available data or gain access to data otherwise unavailable publicly (e.g., current studies underway, disaggregated data from surveillance and other regularly produced reports). We prepared a web-based survey specific to each of the six cities. For each parameter in question, we provided the specific definition of the parameter and the best publicly available data to populate it. Scientific advisory committee members were asked to (i) identify additional sources that we had overlooked or that were not in the public domain but could be made available to the study team, (ii) rate their confidence in proposed triangulation methods to estimate parameter values, (iii) explain their rated responses, and (iv) where possible, share the survey with other knowledgeable public health experts in their professional networks (S1 Supplement).

## Results

**Fig 2:**
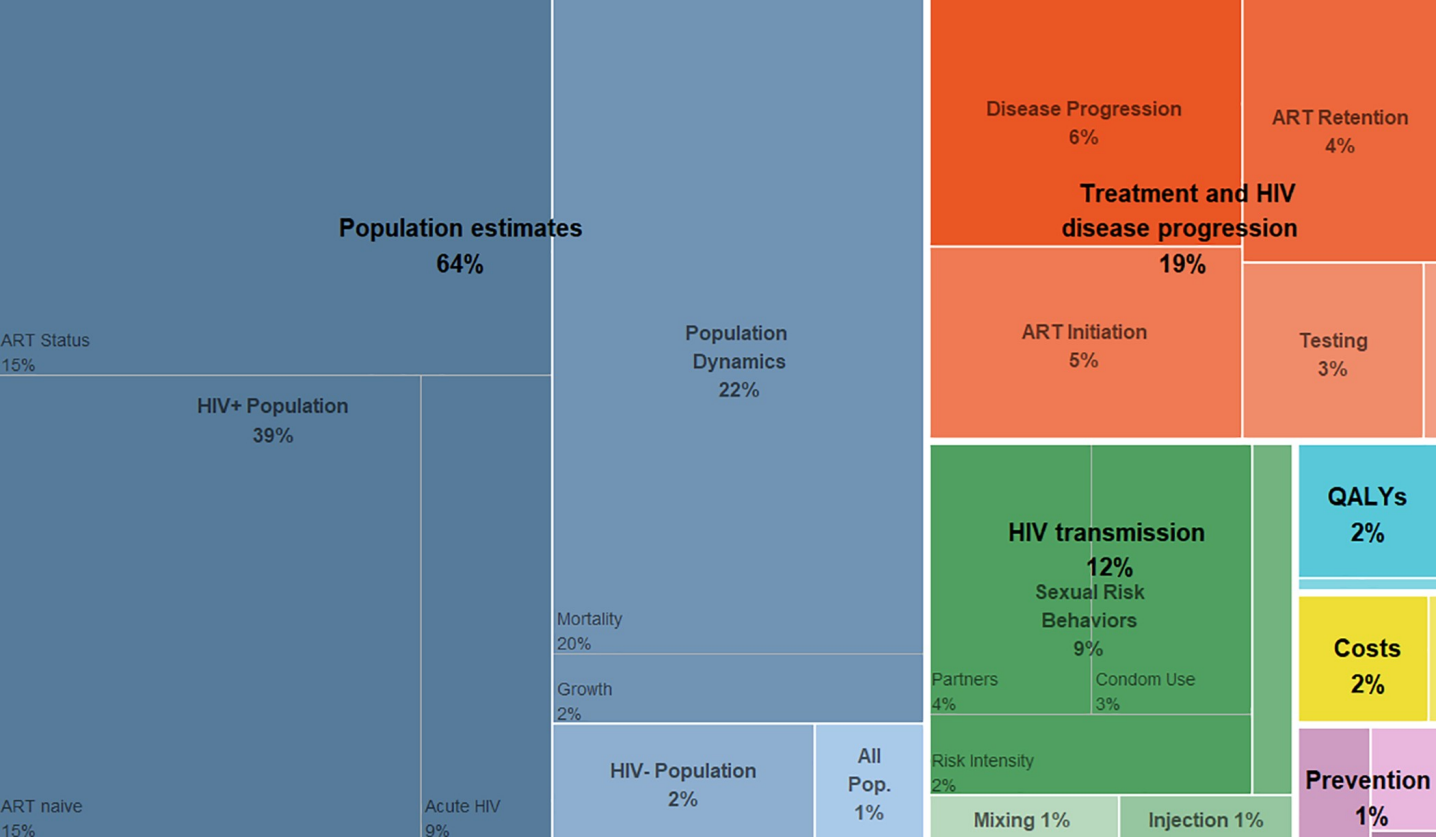
Model parameter category proportions. The boxes are proportionally scaled to the corresponding model parameter category sizes. Model parameter category labels: Population estimates ‒ 1. Initial HIV-negative and HIV-infected population estimates; HIV transmission ‒ 2. Parameters used to calculate the probability of HIV transmission; Treatment and HIV disease progression ‒ 3. Screening, diagnosis, treatment and HIV disease progression; Prevention ‒ 4. HIV prevention programs, including syringe service programs (SSP), OAT, and PrEP; Costs ‒ 5. The costs of medical care for HIV-negative and HIV-infected individuals; and QALYs ‒ 6. Health utility weights for each stage of HIV disease progression. ART: Antiretroviral treatment; All Pop.: Census population estimates; QALYs: Quality-adjusted life-years; Mixing: Sexual mixing patterns.

We identified 1,667 parameters needed to populate our dynamic, compartmental HIV transmission model (Table 1). Of these, 1,517 (91%) were unique to each city and the other 150 (9%) were common for all cities. The proportion of model parameters that composed each of the six model parameter categories varied extensively (Fig 2).

**Table 1:** Summary of model parameters and evidence quality ranking.

Model parameter category	Description of parameters	Number of common parameters	Best- or moderate-quality (%)	Number of city-specific parameters	Best- or moderate-quality (%)						Total by category
					**ATL**	**BAL**	**LA**	**MIA**	**NYC**	**SEA**	
***1*. *Initial population estimates and population dynamics***											
1.1 Risk-stratified population estimates	Total population	0	- -	18	100%	83%	100%	100%	100%	100%	18
1.2 Number of PLHIV	PLHIV population infected/unaware, diagnosed, and on-ART	84	0%	558	71%	74%	74%	71%	74%	74%	642
1.3 Population dynamics	Population entry/maturation rates, mortality rates and migration rates	0	- -	372	26%	26%	26%	26%	26%	26%	372
1.4 HIV-negative population	HIV-negative population with proportion who were screened	1	- -	42	14%	14%	14%	14%	43%	14%	43
***2*. *Parameters used to calculate the probability of HIV transmission***											
2.1 Sexual risk behaviors	Proportion of high/low sexual risk, number of same and opposite sex sexual partners, reduction in sexual partners due to diagnosis	1	100%	156	19%	8%	19%	19%	19%	19%	157
2.2 Injection risk behaviors	Number of injections, proportion of shared injections	1	100%	10	90%	0%	90%	90%	90%	90%	11
2.3 Sexual mixing patterns	Assortativity for heterosexual, MSM, and injection	0	- -	12	0%	0%	0%	0%	0%	0%	12
2.4 Probability of transmission	Probability of tranmission through sexual contact and injection, reduced transmission due to ART, and condom effectiveness	21	100%	0	- -	- -	- -	- -	- -	- -	21
***3*. *Screening*, *diagnosis*, *treatment and HIV disease progression***											
3.1 HIV testing	Rates of HIV testing, increased testing for high-risk, and symptom-based case finding rate	2	0%	42	14%	14%	14%	14%	43%	14%	44
3.2 ART initiation	ART initiation proportion at diagnosis and ART initiation rate for PLHIV who do not immediately initiate ART	0	- -	84	100%	100%	100%	100%	100%	100%	84
3.3 ART retention and re-initiation	Rates of ART retention and ART re-initiation post-dropout	0	- -	72	100%	100%	100%	100%	100%	100%	72
3.4 HIV disease progression on ART	Disease progression for diagnosed (on ART)	0	- -	108	100%	100%	100%	100%	100%	100%	108
3.5 HIV disease progression off ART	Disease progression for diagnosed (off ART), infected/unaware, and acute to chronic HIV for infected and diagnosed	4	100%	0	- -	- -	- -	- -	- -	- -	4
***4*. *HIV prevention programs***											
4.1 Syringe service programs coverage	Total syringe distribution volume	0	- -	1	0%	100%	100%	0%	100%	100%	1
4.2 Opioid agonist treatment (OAT)	Number of PWID/MWID receiving OAT, OAT entry/dropout rates, OAT effectiveness on ART adherence and reduction of shared injections	3	100%	9	0%	100%	100%	100%	100%	100%	12
4.3 Pre-exposure prophylaxis (PrEP)	PrEP uptake; PrEP effect on testing and risk of infection	3	33%	7	100%	0%	100%	100%	100%	100%	10
***5*. *Costs of medical care***											
5.1 HIV-infected	Costs of medical care among PLHIV	0	- -	24	0%	100%	100%	0%	100%	100%	24
5.2 HIV-negative	Costs of medical care for HIV-negative individuals; multiplier for PWID	0	- -	2	0%	100%	100%	100%	100%	100%	2
***6*. *Health utility weights***											
6.1 HIV-infected	Health utility weights for infected, diagnosed, on-ART by HIV disease severity; multipliers for PWID and OAT	27	100%	0	- -	- -	- -	- -	- -	- -	27
6.2 HIV-negative	Reference health state for HIV-infected; multipliers for PWID and OAT	3	0%	0	- -	- -	- -	- -	- -	- -	3
*** ***											
***Total***		**150**	39%	**1517**	56%	56%	58%	57%	60%	58%	**1667**

ATL: Atlanta, Georgia; BAL: Baltimore, Maryland; LA: Los Angeles, California; MIA: Miami, Florida; NYC: New York City, New York; SEA: Seattle, Washington; PLHIV: People living with HIV; ART: Antiretroviral therapy; MSM: Men who have sex with men; PWID: People who inject drugs; MWID: MSM PWID.

To inform each parameter’s point estimate and range, we synthesized evidence from 59 peer-reviewed publications and 24 public health and surveillance reports (Table 2) and executed primary analyses using 11 data sets (Table 3). Parameters ranked as best- to moderate-quality evidence comprised 39% of the 150 common parameters (Table 1) and 13% were directly estimated in the literature (Table 4). City-specific parameters that were ranked as best- to moderate-quality evidence ranged from 56% of the parameters for Baltimore to 60% for New York City (Table 1).

**Table 2:** Data sources used for each risk group, by model parameter category.

	ALL*	MSM	PWID	MWID	HET		ALL*	MSM	PWID	MWID	HET
***1*. *Initial population estimates and population dynamics***						***3*. *Screening*, *diagnosis*, *treatment and HIV disease progression (continued)***					
*1*.*1 Risk-stratified population estimates*						*3*.*2 ART initiation*	* *	* *	* *	* *	* *
United States Census Bureau	X					Medical Monitoring Project (MMP)		X	X	X	X
Peer-reviewed literature		X	X	X		HIV Research Network (HIVRN)		X	X	X	X
National HIV Behavioral Surveillance (NHBS) system			X	X		Local surveillance reports		X	X	X	X
*1*.*2 PLHIV (diagnosed and undiagnosed)*						State surveillance reports		X	X	X	X
Local surveillance reports		X	X	X	X	*3*.*3 ART retention and re-initiation*	* *	* *	* *	* *	* *
State surveillance reports		X	X	X	X	HIV Research Network (HIVRN)		X	X	X	X
Centers for Disease Control and Prevention (CDC)	X					*3*.*4 HIV disease progression on ART*	* *	* *	* *	* *	* *
HIV Research Network (HIVRN)		X	X	X	X	HIV Research Network (HIVRN)		X	X	X	X
*1*.*3 Population dynamics*						*3*.*5 HIV disease progression off ART*	* *	* *	* *	* *	* *
Public health surveillance reports	X					Peer-reviewed literature		X	X	X	X
HIV Research Network (HIVRN)		X	X	X	X	***4*. *HIV prevention programs***					
Peer-reviewed literature			X	X		*4*.*1 Syringe service programs coverage*	* *	* *	* *	* *	* *
United States Census Bureau	X					Local surveillance reports			X	X	
*1*.*4 HIV-negative population*						State surveillance reports			X	X	
United States Census Bureau	X					National surveillance reports			X	X	
National HIV Behavioral Surveillance (NHBS) system		X	X	X		Centre for Disease Control and Prevention (CDC)			X	X	
***2*. *Parameters used to calculate the probability of HIV transmission***						Local data			X	X	
*2*.*1 Sexual risk behaviors*	* *	* *	* *	* *	* *	*4*.*2 Opioid agonist treatment (OAT)*	* *	* *	* *	* *	* *
National HIV Behavioral Surveillance (NHBS) system		X	X	X	X	SAMHSA Treatment Episode Data Sets (TEDS)			X	X	
National Survey of Family Growth (NSFG)					X	National Survey of Substance Abuse Treatment Services (N-SSATS)			X	X	
Peer-reviewed literature	X										
The AIDS linked to IntraVenous Experience (ALIVE) cohort				X		Peer-reviewed literature			X	X	
Project AWARE		X	X	X	X	*4*.*3 Pre-exposure prophylaxis (PrEP)*	* *	* *	* *	* *	* *
*2*.*2 Injection risk behaviors*	* *	* *	* *	* *	* *	Peer-reviewed literature		X		X	
National HIV Behavioral Surveillance (NHBS) system			X	X		Centers for Disease Control and Prevention (CDC)		X		X	
*2*.*3 Sexual mixing patterns*	* *	* *	* *	* *	* *	AIDSVu		X		X	
National HIV Behavioral Surveillance (NHBS) system		X		X	X	***5*. *Costs of medical care***					
National Survey of Family Growth (NSFG)					X	*5*.*1 HIV-infected*	* *	* *	* *	* *	* *
Peer-reviewed literature		X	X	X		HIV Research Network (HIVRN)		X	X	X	X
*2*.*4 Probability of transmission*	* *	* *	* *	* *	* *	Centers for Medicare and Medicaid Services	X				
Peer-reviewed literature		X	X	X	X	Healthcare Cost and Utilization Project (HCUP)	X				
World Health Organization (WHO)	X					VA FSS Price Schedule	X				
***3*. *Screening*, *diagnosis*, *treatment and HIV disease progression***						Peer-reviewed literature	X				
*3*.*1 HIV testing*	* *	* *	* *	* *	* *	*5*.*2 HIV-negative*	* *	* *	* *	* *	* *
National HIV Behavioral Surveillance (NHBS) system		X	X	X		Medical Expenditure Panel Survey (MEPS)	X				
Behavioral Risk Factor Surveillance System (BRFSS)		X			X	Peer-reviewed literature			X	X	
New York City Community Health Survey (NYC-CHS)					X	***6*. *Health utility weights***					
Peer-reviewed literature	X					Peer-reviewed literature	X		X	X	

* All signifies that evidence source used was not stratified by HIV risk group. AWARE: HIV Rapid Testing & Counseling in Sexually Transmitted Disease Clinics; PLHIV: People living with HIV; ART: Antiretroviral therapy; MSM: Men who have sex with men; PWID: People who inject drugs; MWID: MSM PWID.

**Table 3:** Primary analyses data sources and analytic methods^†^.

Analytic sample	Stratification ^a^	Model Parameter Categories	Data access and analytic methods	Year
**NHBS-MSM:** male (≥ 18 years) reporting any oral or anal sex with a male partner during lifetime (venue-based sampling)				
18–64 years old HIV-unaware males reporting a male partner in L12M	5 cities ^d^; MSM, MWID	1.1 Risk-stratified population estimates 2.1 Sexual risk behaviors, L12M 3.1 HIV testing, L12M 4.3 Prescribed PrEP, L12M	Indirect ^b^; Summary statistics	2011, 2014
**NHBS-PWID:** adults (≥18 years) reporting any non-prescribed injection drug use in L12M (respondent-driven sampling)				
18–64 years old HIV-unaware participants injecting drugs in L12M	5 cities ^d^; PWID, MWID	1.1 Risk-stratified population estimates 2.1 Sexual risk behaviors, L12M 2.2 Injection risk behaviors, L12M 2.3 Sexual mixing patterns 3.1 HIV testing, L12M 4.3 Prescribed PrEP, L12M	Indirect ^b^; Summary statistics	2012, 2015
**NHBS-HET:** 18–60 years olds in poverty areas reporting vaginal or anal sex with an opposite sex partner in L12M (respondent-driven sampling)				
18–60 years old HIV-unaware participants who had sex in L12M	5 cities ^d^; HET	2.1 Sexual risk behaviors, L12M 2.3 Sexual mixing patterns	Indirect ^b^; Summary statistics	2013, 2016
**MMP:** adults (≥ 18 years) receiving HIV care (not necessarily on-ART) from HIV clinics in select US states				
18–64 years old HIV-aware	2 cities (NYC and LA); 3 states (GA, FL, WA)	3.2 ART initiation	Indirect ^b^; Summary statistics	2010, 2014
**HIVRN:** individuals enrolled in a consortium of adult and pediatric HIV clinics in the US (multi-site cohort study)				
15–64 years old participants enrolled between 2007 and 2015	3 regions (Northeast, South, West)	1.3 Population dynamics 3.2 ART retention 3.4 Disease progression on ART 5.1 Costs of medical care	Direct ^c^; Multivariable continuous-time multi-state Markov model	2007–15
**AWARE:** HIV-/unaware patients (≥ 18 years) seeking services from STD clinics in nine US cities (randomized controlled trial)				
All participants aged between 18–64 years attending STD clinics	3 cities (LA, Miami, Seattle)	2.1 Sexual risk behaviors, L6M	Indirect ^b^; Summary statistics	2010
**ALIVE:** adults (≥ 18 years) reporting injection drug use within the past 11 years in Baltimore (prospective cohort; community outreach recruited)				
18–64 years old HIV-unaware B/AA participants injecting drugs in L6M	Baltimore; PWID, MWID	2.1 Sexual risk behaviors, L6M	Direct ^c^; Summary statistics	2010
**NSFG:** 15–44 years old men and women from households in the US (stratified multi-stage area probability sampling)				
All participants, excluding those reporting injection drug use in L12M	4 regions (Northeast, South, West, Midwest); HET, MSM	2.1 Sexual risk behaviors, L12M 2.3 Sexual mixing patterns	Direct ^c^; Weighted summary statistics	2011–13
**BRFSS:** adult (≥ 18 years) telephone respondents from households in the US (disproportionate stratified sampling and random sampling)				
18–64 years old HIV-unaware	6 states; HET	3.1 HIV testing, L12M	Direct ^b^; Weighted summary statistics	2010
**NYC CHS:** adults (≥ 18 years) telephone respondents from households in New York City (stratified random sampling)				
18–64 years old HIV-unaware	New York City; HET	3.1 HIV testing, L12M	Direct ^b^; Weighted summary statistics	2010
**TEDS:** all individuals admitted to treatment facilities for substance use disorder that receive public funds (national client-level database)				
≥15 year old reporting OUD	5 cities ^e^; PWID	4.2 Number of individuals receiving OAT	Direct ^b^; Summary statistics	2010–14

† Further details are presented in S1 Supplement

^a^ If not specified, applicable to all risk groups of interest and/or cities

^b^ Summary statistics provided by the principal investigators of the databases

^c^ We accessed the data and performed analysis

^d^ NHBS data were not available for Baltimore

^e^ All OAT data were missing for Georgia.

NHBS: National HIV Behavioral Surveillance; MMP: Medical Monitoring Project; AWARE: HIV Rapid Testing & Counseling in Sexually Transmitted Disease Clinics in the US; ALIVE: AIDS Linked to IntraVenous Experiences study; NSFG: National Survey of Family Growth; HIVRN: HIV Research Network; HET: heterosexual; MSM: men who have sex with men; PWID: people who inject drugs; MWID: MSM who inject drugs; B/AA: black/African American; PrEP: Pre-exposure prophylaxis; L12M: last 12 months; L6M: last 6 months; STD: sexually transmitted diseases; OAT: Opioid agonist treatment; OUD: Opioid use disorder; GA: Georgia; FL: Florida; WA: Washington; LA: Los Angeles; NYC: New York City.

**Table 4:** Quality assessment for model parameters common across cities.

Model Parameter Category	Available Evidence	Best-Quality Evidence	Source
***1*. *Initial population estimates and population dynamics***	*** ***	*** ***	*** ***
1.2 Proportion of acute state among diagnosed	II—B	III—A	[40]
1.2 Proportion of acute state among infected	II—B	III—A	[40]
***2*. *Parameters used to calculate the probability of HIV transmission***	* *	* *	* *
2.1 Percentage decrease in number of sexual partners due to diagnosis	IV—A	IV—A	[41]
2.2 Reduced probability of shared injections due to HIV diagnosis	IV—A	IV—A	[42]
2.4 Probability of transmission per partnership from female to male†	IV—B	IV—A	[43–54]
2.4 Probability of transmission per partnership from male to female†	IV—B	IV—A	
2.4 Probability of transmission per partnership same sex†	IV—B	IV—A	[55–59]
2.4 Condom effectiveness for heterosexual sex	IV—A	IV—A	[60]
2.4 Condom effectiveness for homosexual sex	II—A	IV—A	[61]
2.4 Reduction in probability of transmission by sex due to ART (HET)	I—A	IV—A	[62]
2.4 Reduction in probability of transmission by sex due to ART (MSM)	I—A	IV—A	[63, 64]
2.4 Probability of transmission per shared injection†	IV—B	IV—A	[65–68]
2.4 Percentage reduction in probability of transmission by injection due to ART	V—A	IV—A	[69]
***3*. *Screening*, *diagnosis*, *treatment and HIV disease progression***	* *	* *	* *
3.1 Symptom-based case finding rate for infected (CD4 200–499*)	II—B	III—A	[70, 71]
3.1 Symptom-based case finding rate for infected (CD4 < 200*)	II—B	III—A	[70, 71]
3.5 Transition rate: acute infected to chronic state infected (CD4 ≥ 500*)	II—A	II—A	[72]
3.5 Transition rate: acute diagnosed to chronic state diagnosed (CD4 ≥ 500*)	II—B	II—A	[72]
3.5 HIV disease progression rate from CD4 ≥ 500* to CD4 200–499* (off ART)	II—B	II—A	[70, 71]
3.5 HIV disease progression rate from CD4 200–499* to CD4 < 200* (off ART)	II—B	II—A	[70, 71]
***4*. *HIV Prevention Programs***	* *	* *	* *
4.2 Percentage reduction in shared injections due to OAT	IV—B	IV—A	[73]
4.2 OAT entry/dropout rate	IV—A	III—A	[74]
4.2 Multiplier for ART dropout rate for individuals on OAT	II—A	IV—A	[75]
4.3 Percentage reduction in risk of infection for individuals on PrEP	I—A	IV—A	[76]
4.3 Screening rates for individuals on PrEP	VI—B	III—A	[77]
4.3 Average duration individuals on PrEP remain identified after screening	VI—B	III—A	[77]
***6*. *Health utility weights***	* *	* *	* *
6.1 HIV-infected non-PWID	II—A	IV—A	[78–83]
6.1 HIV-infected PWID/OAT	II—B	IV—A	[69, 84]
6.2 HIV-negative non-PWID	VI—B	IV—A	Assumption
6.2 HIV-negative PWID/OAT	VI—B	IV—A	[69, 84]

† for acute and chronic disease states

* cells/μL. ART: Antiretroviral therapy; HET: Heterosexual; MSM: Men who have sex with men; PrEP: Pre-exposure prophylaxis; SSP: Syringe services program; OAT: Opioid agonist treatment; PWID: People who inject drugs.

Type of evidence: I—Single randomized clinical trial; II—Single non-randomized trial/cohort study; III—Administrative database; IV—Systematic review/meta-analysis of multiple RCTs or cohort studies; V—Cost-effectiveness analysis; VI—Expert opinion/assumption

Types of evidence adapted from Oxford Centre for Evidence-based Medicine–Levels of Evidence [39].

Derivation method: A—Model parameter values directly available from literature; B—Model parameter values triangulated from multiple sources.

### Results of data verification

The city-specific surveys that were sent to our scientific advisory committee contained questions about four of the six model parameter categories, including questions regarding population size estimates for HIV risk groups, parameters used to calculate the probability of HIV transmission, ART dropout rates and HIV prevention programs (S1 Supplement). Each city had at least one scientific advisory committee representative respond, and two cities had multiple respondents participate. Responses helped guide triangulation methods, and updated parameter estimates were re-sent to scientific advisory committee members for final review so that they could see where their responses were incorporated and how parameter values were used in calibration and/or sensitivity analysis.

### Evidence synthesis for model parameter categories

Key results of our evidence synthesis are highlighted by model parameter category in this section. A detailed description of the derivation of each individual parameter is provided in the supplementary material (S1 Supplement), and descriptions of all datasets used in primary analysis can be found in Table 3 and in the supplementary material (S1 Supplement).

#### Initial population estimates and population dynamics

A majority of the model’s parameters (1,075; 64%) were for population estimates and population dynamics. We derived the necessary evidence from 13 public health and surveillance reports [85–98] and 9 peer-reviewed publications [40, 99–106] and from primary analyses of 5 datasets [42, 107–110] (Table 2). More than two-thirds of the city-specific evidence used for the 558 parameters (38% of all parameters) that established the size of PLHIV populations in the model were of best- or moderate-quality (Table 1). However, the limited evidence available to determine the proportion of HIV-infected individuals in the acute stage of HIV disease progression was of low-quality (Table 4), and these parameters (n = 84) represented half of all parameters common across cities (Table 1). Approximately one-quarter of the city-specific parameters that determined the probabilities of mortality (n = 372) from each health state were of best- or moderate-quality, including primary analyses of data from The HIV Research Network (HIVRN) that were used to derive mortality rates for PLHIV receiving ART [107, 111].

#### Parameters used to calculate the probability of HIV transmission

We synthesized evidence from 29 peer-reviewed publications [41, 43, 45–60, 65–69, 112–117] (Table 2) and conducted primary analyses of 6 datasets [42, 108, 110, 118–120] (Table 3) to derive the parameters that determined the probability of HIV transmission (n = 224; 13% of total parameters). The probabilities for HIV transmission per shared injection or sexual act and the effectiveness of HIV-related interventions (i.e., condom use, OAT, SSP and ART)–all common across cities–were derived from the peer-reviewed literature. All common parameters (n = 41) were of best- or moderate-quality (Table 1), and approximately a third (n = 7, 30%) were directly estimated (Table 4). In contrast, best- or moderate-quality evidence that informed city-specific sexual risk behavior parameters (n = 157) ranged from 8% to 19%, and evidence for sexual mixing pattern parameters (n = 12) was of low-quality across all cities (Table 1). Estimates of sexual risk behavior were obtained from National HIV Behavioral Surveillance (NHBS) data for MSM and PWID [42, 110] and we used region-specific population-based data from the National Survey of Family Growth (NSFG) for heterosexuals [108]. To determine ranges used in sensitivity analyses and calibration, we supplemented this evidence with primary analyses of data from the AIDS Linked to IntraVenous Experience (ALIVE) PWID cohort study [120] and from Project AWARE [119]. Lastly, we estimated injection risk behavior using NHBS data [42].

#### Screening, diagnosis, treatment and HIV disease progression

**Fig 3:**
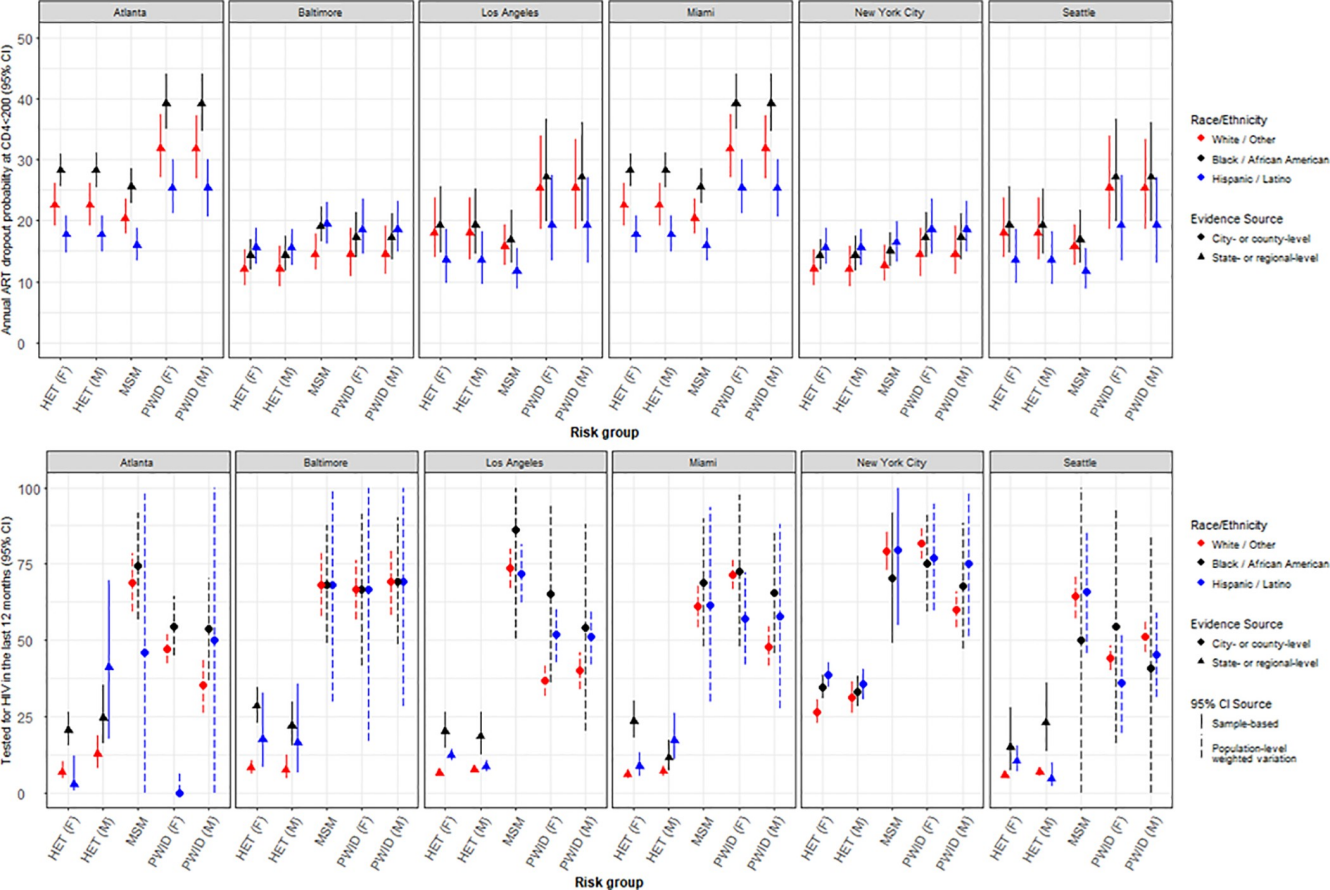
Heterogeneity in selected parameter estimates by city, risk group, gender and race/ethnicity. MSM: Men who have sex with men; PWID: People who inject drugs; HET: Heterosexuals; ART: Antiretroviral treatment; F: Female; M: Male.

Screening, diagnosis, treatment and HIV disease progression parameters represented 18% (n = 312) of all model parameters and were derived from 5 peer-reviewed publications [71, 72, 111, 121–123] and 6 public health and surveillance reports [86, 88, 90, 96–98] (Table 2) and from primary analyses using 6 data sets [42, 107, 109, 110, 124, 125] (Table 3). HIV testing rates (n = 42) were derived from primary analyses of sample data from NHBS [42, 110], the US Centers for Disease Control and Prevention’s (CDC) Behavioral Risk Factor Surveillance System [109], and the New York City Community Health Survey [125]. Notably, best- or moderate-quality evidence for stratified population-level testing rates was sparse (Table 1). In the absence of city-specific ART data, we used corresponding regional HIVRN data to obtain rates of ART initiation (n = 84) and re-initiation (n = 18) [107]. We also used HIVRN data with continuous-time multi-state Markov models to populate parameters specific to HIV disease progression rates while PLHIV are on ART (n = 108) and in relation to ART dropout rates (n = 54). The rates of HIV testing and ART dropout varied extensively across cities and across risk groups and races/ethnicities within cities (Fig 3). Rates of ART initiation were supplemented using analyses of Medical Monitoring Project (MMP) data [124], and disease progression off-ART was estimated using peer-reviewed literature [71] (Table 4).

#### HIV prevention programs

**Fig 4:**
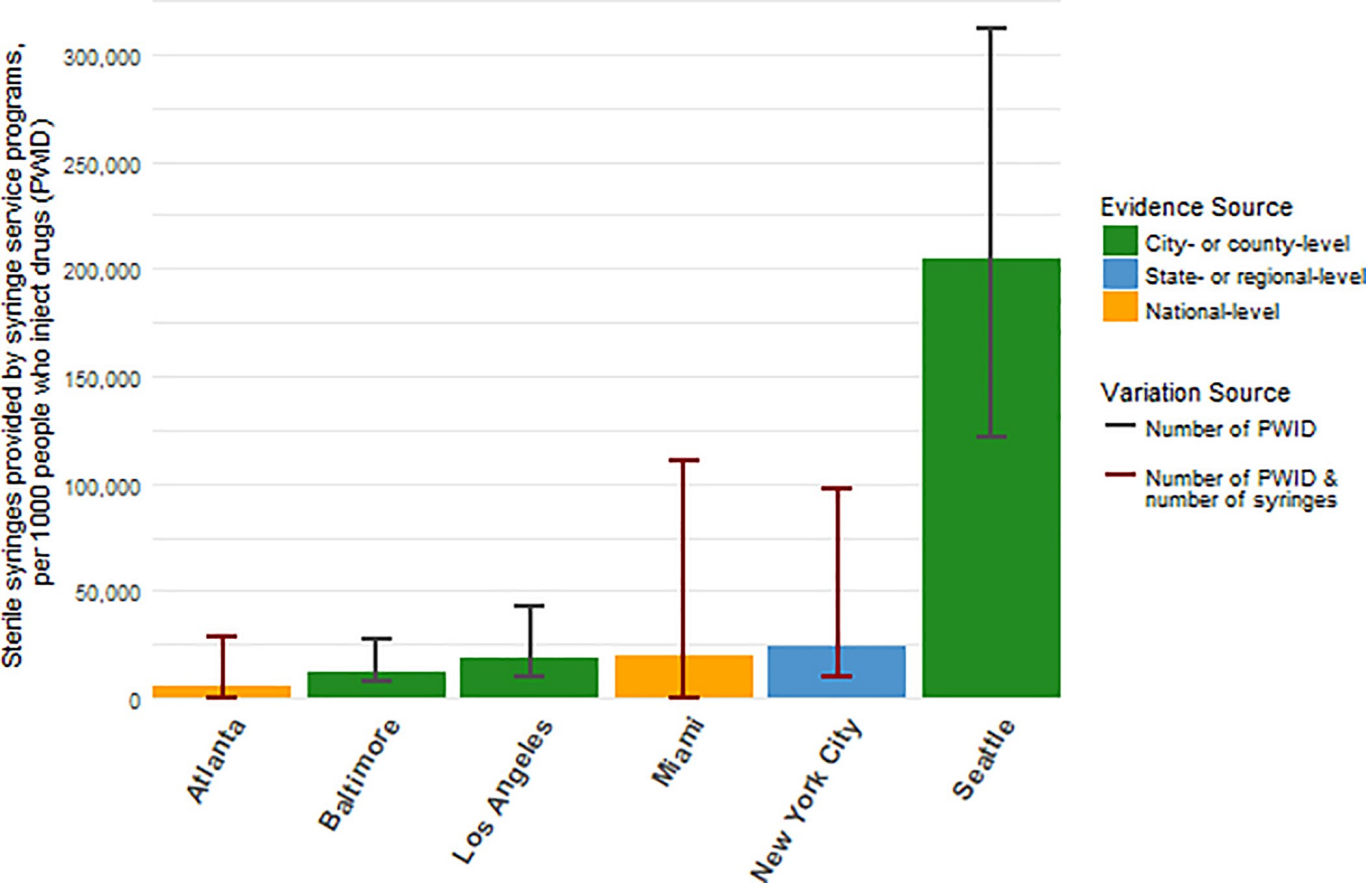
Coverage of sterile syringes programs for people who inject drugs.

Parameters for HIV prevention programs (n = 23) were derived by combining evidence from 10 local, state and national sources [77, 126–134], 7 peer-reviewed articles [73, 74, 135–139], 2 publicly accessible data sources [126, 127] (Table 2). Availability of local data sources to populate syringe distribution parameters varied greatly, and we found extensive variation across cities in relation to the availability of syringes per 1,000 PWID (Fig 4). We used state-level data from the Substance Abuse and Mental Health Services Administration combined with evidence from the peer-reviewed literature to derive the number of PWID and MWID receiving OAT with either methadone or buprenorphine [126, 127, 135, 137]. Common parameters for the protective effects of HIV prevention programs were of good quality and often directly estimated in the peer-reviewed literature (Table 4). Lastly, we used AIDSvu data to determine pre-exposure prophylaxis (PrEP) uptake [134].

#### Costs of medical care

To quantify health resource use cost parameters for infected (n = 24) and HIV-negative (n = 2) individuals, we included evidence from 4 public health and surveillance reports [140–143] and 4 peer-reviewed publications [28, 144–146] and conducted primary analysis of 1 dataset[147] (Table 2). For each city, we used corresponding regional HIVRN patient level utilization data and corresponding unit costs to estimate quarterly health care costs for HIV-infected individuals (Table 3). For HIV-negative individuals, we stratified cost estimates for MSM and HET risk groups from regional Medical Expenditure Panel Survey (MEPS) data [140] and used a multiplier to estimate costs for PWID [28].

#### Health utility weights

We used common health utility weights across all six cities to calculate quality adjusted life years (QALYs) parameters (n = 30), synthesizing evidence from 4 peer-reviewed articles (Table 4). Estimates were derived from a nationally representative sample of QALY estimates [82], a meta-analysis [83], and a study that used a sample of Veterans Affairs members to estimate the change in health-related quality of life when patients were diagnosed and became aware of their HIV status [79, 82, 83]. We also incorporated evidence from the literature to establish a framework for relevant health states in HIV infection and disease progression [78]. Evidence from two additional studies was used to update the weights used for individuals receiving treatment in the modern era of highly active ART [80, 81]. Lastly, we used QALY weight multipliers for PWID based on whether or not they were receiving OAT [69, 84]. While these sources were the best-available evidence for health utility weights, the majority were published prior to 2007. Notably, health utility weights among PLHIV may have changed over time as a result of modern advances in treatment and HIV care.

#### Calibration and validation targets

We identified 3 sets of calibration targets (17 targets in total), including stratified indicators of the annual number of new HIV diagnoses per year, the total number of diagnosed PLHIV and the annual number of all-cause deaths among PLHIV (S1 Supplement). These were representative of some of the best-quality data available and were also important to long-term clinical and epidemiological projections of city-level microepidemics, consistent with guidelines on selecting calibration and validation targets in cost-effectiveness analysis [19]. Furthermore, point estimates and ranges of HIV prevalence were used as validation targets to ensure external validity.

## Discussion

We have provided a comprehensive description of an extensive evidence synthesis process that is required to populate a dynamic, compartmental HIV transmission model for six US cities. We identified differences across cities in the quality and representativeness of evidence available to inform our model. However, we identified consistency in the lack of availability of best-quality local administrative data that are critical to assess health system performance, particularly in relation to population-level rates of HIV testing and ART engagement. Nonetheless, our findings, which used the best-available evidence, highlight fundamental differences across settings related to rates of health system engagement and access to HIV prevention programs. The modeling of targeted, locally-oriented combination implementation strategies is necessary to determine how scarce resources should be allocated to interventions that can provide the greatest value for money in a given microepidemic. Our findings emphasize the need for increased public health efforts to measure and monitor the most informative components of local HIV prevention and care services, including the delivery, uptake and effect of localized HIV programs.

Reviews of health economic models in specific disease areas typically focus on differences in model structures and projected outcomes, with limited discussion of how the differences in the quality of input data can function as an explanation for variations across outputs [148–152]. Failure to report the sources and quality of model parameters, or reporting evidence directly from other modelling exercises without assessing the quality and representativeness of the inputs, can limit the interpretability of a model thereby eroding the confidence of its recommendations [153]. Cooper et al. (2007) discussed three practical issues and methodological challenges related to the use of evidence in health decision models: (i) defining and identifying ‘relevant’ evidence, (ii) assessing the quality and relevance of different sources of evidence, and (iii) synthesizing the evidence for use in modeling exercises [24]. Their paper also provided practical recommendations to address these challenges. Namely, a) describe the search method and selection process used to identify ‘relevant’ evidence per parameter; b) evaluate the quality and representativeness of the data retrieved; and c) pool evidence using explicit criteria where applicable [24]. Decision makers must be able to interpret mathematical models to use them to develop and evaluate effective HIV responses [154]. We documented our evidence synthesis process for six different cities as comprehensively as possible for transparency and reproducibility. We hope this effort promotes the use of modeling recommendations in decision making processes that address city-level HIV microepidemics.

The sustainability of an effective and efficient HIV response is critical to the control of local microepidemics [155–157]. For instance, the benefits of a treatment intervention that increases ART engagement might only be maximized with a sufficient level of HIV testing. Modeling recommendations promoting locally-oriented combination implementation strategies depend on evidence from local health systems. Reliable evidence of interactions between PLHIV and local health systems provided by surveillance and administrative data would greatly enhance the validity of modeling recommendations. Furthermore, and despite a paucity of behavioral data available describing sexual risk behavior and race/ethnicity mixing, recent evidence suggests that racial assortativity alone cannot adequately explain observed disparities in HIV incidence [158]. However, a meaningful share of this disparity can be explained by differential ART engagement by race [158]. This discrepancy further highlights the need for an improved use of routinely collected surveillance data (e.g., laboratory viral load monitoring can be used as a reasonable proxy of ART engagement) to allow for a better understanding of how to improve HIV care. Similarly important for locally-oriented modeling recommendation, city-level estimates of population sizes for PWID and MWID risk groups are either completely lacking or in critical need of updating [102]. Varying assumptions about risk groups in the modeling of epidemic dynamics can alter cost-effectiveness conclusions and intervention recommendations despite good model calibration [159], suggesting the need for a careful assessment of the potential value of collecting data about subpopulations that can have a disproportionate impact on local microepidemics. These examples of imperfect or missing data underline the importance of the data collection efforts of the US CDC’s NHBS and MMP, which provide behavioral information about people at risk of HIV and disease and treatment status of PLHIV [42, 110, 160].

Systematically conducting one-way sensitivity analyses and probabilistic sensitivity analysis to quantify the uncertainty in model recommendations resulting from parameters derived from evidence of poorer quality or representativeness is critical for further information gathering [34]. Value of information analysis [161] should guide the identification of influential parameters requiring additional research to reduce uncertainty in the decision making process. This exercise can also help define the collection of city-specific HIV surveillance data [162]. Data source identification and parameter estimate derivation should therefore be reported in sufficient detail to allow readers with the necessary expertise to perform a detailed evaluation of the model and possibly replicate it [19]. Ultimately, the development of formal guidelines pertaining to the evidence synthesis process and how it informs decision models should result in increased guidance for those engaged in the reporting process. These guidelines would include the creation of a standardized checklist that emulates the Consolidated Health Economic Evaluation Reporting Standards [163]. Publishers now routinely require this kind of checklist in cost-effectiveness studies.

This comprehensive evidence synthesis process had several potential limitations. First, the search for evidence sources was not systematic; however, we used a systematic structured identification strategy [24]. Furthermore, we included best-quality evidence sources from a narrative review of high-impact, current and diverse HIV models to further inform our initial identification process [164] in order to mitigate the potential correlation between sources. Second, we have not assessed the impact that the uncertainty in poorer quality parameters could have on model recommendations since this was beyond the scope of this study but rather report these findings in work elsewhere [32]. Third, despite a data verification process involving a scientific advisory committee composed of city-specific experts that helped to resolve instances where evidence was of poor representativeness, the number of respondents involved was low. Future evidence syntheses could benefit from a broad inclusion of public health officials. Lastly, given the relative wealth of surveillance data sources in the United States, the extent to which this evidence synthesis process could be replicated needs to be assessed on a case-by-case basis. As the possibilities for exercises of similar scope continue to grow in other regions and disease areas, our reporting framework can bolster future efforts.

Better integration of modelling in decision making can be achieved by systematically reporting on the evidence synthesis process that is used to populate models and by explicitly assessing the quality of data. The effective communication of this process can help prioritize data collection of the most informative components of local HIV prevention and care services in order to reduce decision uncertainty and strengthen model conclusions.

## Supporting information

S1 Supplement Supporting information includes descriptions of city boundaries (Supplement A), search strategy for model inputs (Supplement B), derivation of all model parameters (Supplement C), description of data sets used for primary analysis (Supplement D), derivation of model calibration/validation targets and PSA probability distributions (Supplement E), and the data verification survey/results from our scientific advisory committee (SAC) (Supplement F).
(PDF)

S2 Supplement **Supplement Tables** Supporting information includes (i) excel file of values, ranges and PSA distributions for all model parameters, (ii) excel file of values and ranges for calibration/validation targets for all cities, and (iii) pdf of full survey given to SAC members for data verification.
(ZIP)
