# Supplementary material for: Developing a dynamic HIV transmission model for 6 U.S. cities: An evidence synthesis
Source: PLoS One. 2019 May 30;14(5):e0217559. doi: 10.1371/journal.pone.0217559 (PMC6542533; doi:10.1371/journal.pone.0217559)
Supplement: S1 Supplement — Supporting information includes descriptions of city boundaries (Supplement A), search strategy for model inputs (Supplement B), derivation of all model parameters (Supplement C), description of data sets used for primary analysis (Supplement D), derivation of model calibration/validation targets and PSA probability distributions (Supplement E), and the data verification survey/results from our scientific advisory committee (SAC) (Supplement F). (PDF) [file pone.0217559.s001.pdf]

S1 Supplement

Developing a dynamic HIV transmission model for 6 U.S. cities: an evidence synthesis

Emanuel Krebs[1], Benjamin Enns[1], Linwei Wang[1], Xiao Zang[1,2], Dimitra Panagiotoglou[1], Carlos Del Rio[3], Julia Dombrowski[4], Daniel J Feaster[5], Matthew Golden[4], Reuben Granich[6], Brandon Marshall[7], Shruti H Mehta[8], Lisa Metsch[9], Bruce R Schackman[10], Steffanie A Strathdee[11], Bohdan Nosyk[1,2], **on behalf of the localized HIV modeling study group**.

1. Health Economic Research Unit at the British Columbia Centre for Excellence in HIV/AIDS; 2. Faculty of Health Sciences, Simon Fraser University; 3. Hubert Department of Global Health, Emory Center for AIDS Research, Rollins School of Public Health, Emory University; 4. Department of Medicine, Division of Allergy & Infectious Disease, adjunct in Epidemiology, University of Washington; 5. Center for Family Studies, Department of Epidemiology and Public Health, Leonard M. Miller School of Medicine, University of Miami; 6. International Association of Providers of AIDS Care; 7. Department of Epidemiology, Brown School of Public Health; 8. Bloomberg School of Public Health, Johns Hopkins University; 9. Department of Sociomedical Sciences, Mailman School of Public Health, Columbia University; 10. Department of Healthcare Policy and Research, Weill Cornell Medical College; 11. School of Medicine, University of California San Diego.

S1 Supplement A: City Boundaries

Availability of highest quality stratified data from surveillance reports determined the inclusion or exclusion of specific counties within each city's boundaries. Counties included in city boundaries for Atlanta, Baltimore, Los Angeles, and Miami match those included in the definition of Ryan White Eligible Metropolitan Area (EMA) or Transitional Grant Area (TGA) while New York City and Seattle boundaries are restricted to a subset of counties for our purposes.

S1 Figure A1. Selected cities

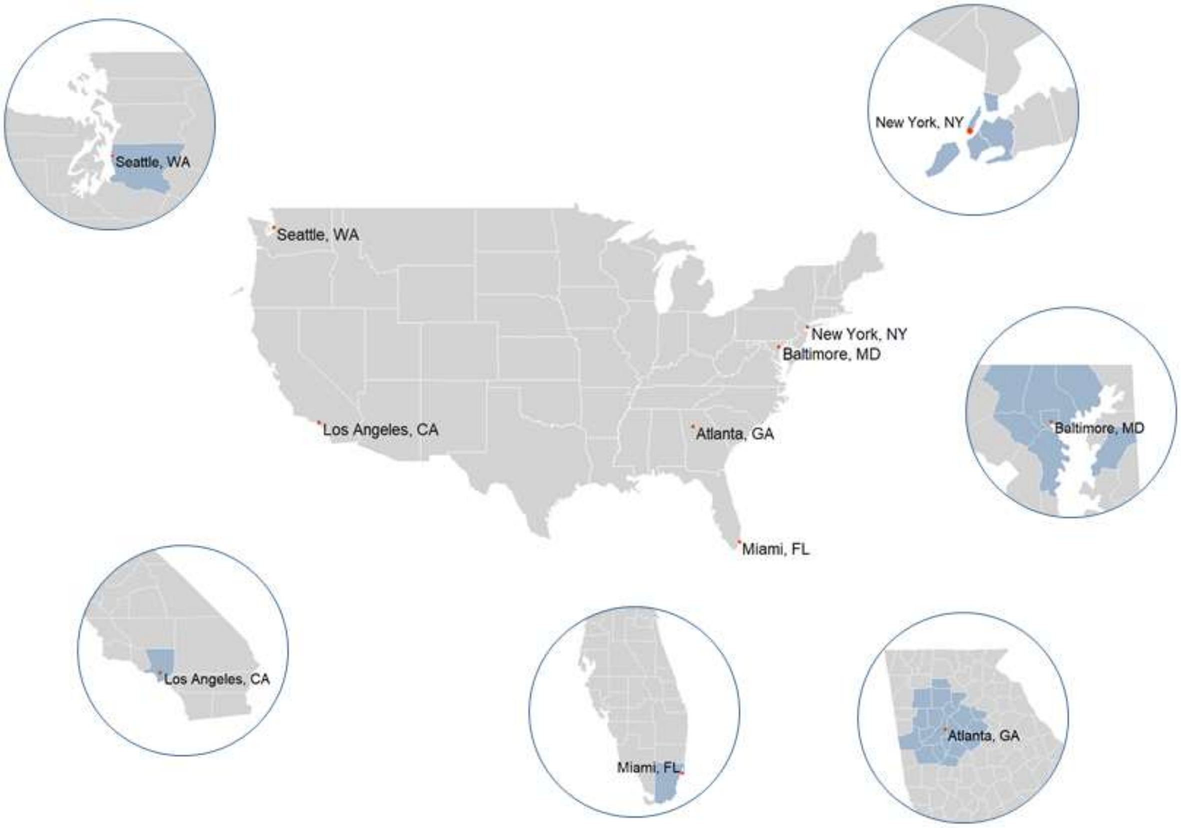

Counties included in each city are found in brackets: Atlanta (Barrow, Bartow, Carroll, Cherokee, Clayton, Cobb, Coweta, DeKalb, Douglas, Fayette, Forsyth, Fulton, Gwinnett, Henry, Newton, Paulding, Pickens, Rockdale, Spalding, Walton); Baltimore (Anne Arundel, Baltimore City, Baltimore County, Carroll, Harford, Howard, Queen Anne's); Los Angeles (Los Angeles county); Miami (Miami-Dade county); New York City (county with borough in brackets: New York [Manhattan], Kings [Brooklyn], Queens [Queens], Bronx [Bronx], Richmond [Staten Island]); Seattle (King county). Excluded counties for New York City compared to Ryan White EMA definition include Westchester, Rockland and Putnam, and excluded counties for Seattle compared to Ryan White TGA definition include Snohomish and Island.

S1 Supplement B: Search Strategy for Model Inputs

We divided our search strategy into two parts: (1) identifying a rank order of potential data sources for each domain, and (2) selection of the best data to use, given additional factors and constraints. We identified the best possible data sources for each domain, ranked by suitability depending on factors unique to each model parameter category [1,2,3]. For example, the most accurate and reliable source for initial total population numbers was city-level census data, while the best source for ART effectiveness estimates came from randomized controlled trials (RCT). For city-specific parameters, we selected source data based on geographic representativeness, stratification level relative to our model requirements, and time-period. For non-city-specific (common) parameters, we selected source data based on study quality, how well the evidence matched the ideal evidence for a given model parameter (Manuscript Table 4), and whether or not our required model parameters could be directly estimated from evidence sources.

For model parameters relating to the probability of HIV transmission (S1 Supplement C, Section 2) condom effectiveness, ART effectiveness, probability of transmission, and disease progression for PLHIV off-ART, we documented our literature search methods, results, and choice of evidence to inform model parameters (S1 Table B2). Specifically, the starting set for our snowball literature search was derived from the references for target parameters previously used in modeling studies [4,5,6]. Literature cited in any of the start set papers were searched using Google Scholar. From this initial search, we defined key words (first restricting to words in titles) and searched for “review” type publications. The search strategies were broadened when the limited search resulted in no relevant publications. As per our a priori selection criteria (S1 Table B1), we prioritized data published from randomized control trials (RCT), and where not available, relied on peer-reviewed observational studies with or without meta-analysis. Where meta-analysis was not performed for the parameter of interest, or when the evidence included in the meta-analysis was heterogeneous across settings, we reviewed the individual studies included, and if necessary calculated parameter estimates (not otherwise reported) using the data reported in the original paper.

S1 Supplement Table B1: Identification and selection criteria for model inputs

Model Parameter Category	Identification Criteria - Quality of Evidence Ranking by Category†	Selection Criteria Ranking by Category‡
**1. Initial population estimates and population dynamics**		
1.1 Risk-stratified population estimates<br>1.2 Number of PLHIV<br>1.3 Population dynamics<br>1.4 HIV-negative population	1. Central statistics, health care system statistics, life tables; 2. Sample-based studies; 3. Expert opinion	1. Geographic representativeness*; 2. Population stratification**; 3. Time-period
**2. Parameters used to calculate the probability of HIV transmission**		
2.1 Sexual risk behaviors<br>2.2 Injection risk behaviors	1. Health care system statistics, population-based surveys; 2. Sample-based studies; 3. Expert opinion	1. Type of evidence***; 2. Time-period
2.3 Sexual mixing patterns	1. Longitudinal cohort studies and case series, nationally collected or compiled statistics or routinely collected admin data, disease registries, or epidemiological database; 2. Data from RCTs; 3. Expert opinion	1. Geographic representativeness*; 2. Population stratification**; 3. Time-period
2.4 Probability of transmission	1. Systematic synthesis of RCTs, single RCTs, or systematic synthesis of cohort studies; 2. Observational studies (e.g. cohort, case control); 3. Non-analytical studies	1. Type of evidence***; 2. Time-period
**3. Screening, diagnosis, treatment and HIV disease progression**		
3.1 HIV testing<br>3.2 ART initiation<br>3.3 ART retention and re-initiation	1. Case series or analysis of reliable admin database; 2. Old case series, estimates from RCTs or other observational studies; 3. Other modeling studies, expert opinion	1. Geographic representativeness*; 2. Population stratification**; 3. Time-period
3.4 HIV disease progression on ART	1. Systematic synthesis of RCTs, single RCTs, or systematic synthesis of cohort studies; 2. Observational studies (e.g. cohort, case control); 3. Non-analytical studies	1. Geographic representativeness*; 2. Population stratification**; 3. Time-period
3.5 HIV disease progression off ART	1. Longitudinal cohort studies and case series, nationally collected or compiled statistics or routinely collected admin data, disease registries, or epidemiological database; 2. Data from RCTs; 3. Expert opinion	1. Type of evidence***; 2. Time-period
**4. HIV prevention programs**		
4.1 SSP coverage<br>4.2 Opioid agonist treatment<br>4.3 Pre-exposure prophylaxis	1. Case series or analysis of reliable admin database; 2. Old case series, estimates from RCTs or other observational studies; 3. Other modeling studies, expert opinion	1. Geographic representativeness*; 2. Population stratification**; 3. Time-period
**5. Costs of medical care**		
5.1 PLHIV<br>5.2 HIV-negative	1. Case series or analysis of reliable admin database; 2. Old case series, estimates from RCTs or other observational studies; 3. Other modeling studies, expert opinion	1. Geographic representativeness*; 2. Population stratification**; 3. Time-period
**6. Health utility weights**		
6.1 PLHIV<br>6.2 HIV-negative	1. Central statistics, health care system statistics, life tables; 2. Sample-based studies; 3. Expert opinion	1. Type of evidence***; 2. Time-period

† Quality rankings for evidence categories 1-4 adapted from Zang et al. (2018) [7], quality rankings for categories 5-6 adapted from Cooper et al. (2005), Zechmeister-Koss et al. (2014) and Paisley (2016) [2,3,8]. ‡ Given the same quality of evidence, geographic representativeness was prioritized for city-specific parameters, while evidence most closely matching the data requirements was prioritized for common parameters.

* Geographic representativeness prioritized as: 1. City/MSA-level, 2. State-level, 3. Regional, 4. National

** Given the same quality of evidence, sources with more granular levels of population stratification were prioritized over sources that aggregated across strata.

*** Type of evidence criteria applied to common parameters and ranked according to data requirements for each category (Manuscript Table 4). Type of evidence: I - Single randomized clinical trial; II - Single non-randomized trial/cohort study; III - Administrative database; IV - Systematic review/meta-analysis of multiple RCTs or cohort studies; V - Cost-effectiveness analysis; VI - Expert opinion/assumption. Types of evidence adapted from Oxford Centre for Evidence-based Medicine – Levels of Evidence [9].

S1 Supplement Table B2: Search strategy for infectivity/transmission parameters

Parameters	[a] Seed papers	Citing articles	Key words	Resulting articles	[b] Relevant articles	[f] Estimates	Triangulation methods	Value (Range)	[k] Evidence type
**Condom effectiveness**									
Heterosexual sex	[10]	851	"allintitle: review AND HIV AND condom"	2	2; [10]; [11]	80.2% (95% CI: 56.3%-91.0%); 71% (95% CI: 57%-80%)	Range estimates capturing all the 95%CI	80% (57%- 91%)	Meta-analysis of observational studies
[c] Homosexual sex	N/A	N/A	(((homosexual) OR men who have sex with men)) AND ((((((condom[MeSH Terms]) AND effectiveness) AND HIV)))	83	2; [12]; [13]	70.5% (95% CI: 58.2%-79.2%); 64% (95% CI: 32%-80%)	Range estimates capturing all the 95%CI	70.5% (58.2%-79.2%)	Prospective cohort and meta-analysis of observational studies
**ART effectiveness**									
Heterosexual transmission	[14]	4617	"allintitle: review AND HIV AND antiretroviral"	11	2; [14]; [15]	96% (95% CI: 73%-99%); 91% (95%CI: 79-96%)	Range estimates capturing all the 95%CI	91% (79%-96%)	Meta-analysis of prospective cohort studies
Homosexual transmission	[16,17]	75	"allintitle: HIV AND antiretroviral"	44	1; [16,17]	100% (95% CI: 99.7%-100%)	Supplemented by evidence from heterosexual transmission.	91% (79%-96%)	Meta-analysis of prospective cohort studies
Needle sharing transmission	[18]				[19,20]; [18]	50% (25%-75%); 50% (10%-90%)	Range estimates capturing all ranges used in the modeling studies	91% (79%-96%)	Modelling studies
**Probability of HIV transmission per shared injection**									
[d] By CD4 strata	[21]; [22]	217; 28	"allintitle: (infectivity OR transmission)"	35; 2	4; [21]; [23]; [24,25]	Overall: 0.0067 ; 0.0063 (95% CI: 0.0041, 0.0092); By CD4: limited evidence.	[h] Triangulated CD4-specific estimates based on the evidence reviewed and assumptions.	CD4≥500: 0.003 (0.0014-0.0092); 200-499: (0.004 (0.0014-0.0092); <200: 0.006 (0.0041-0.02).	Systematic review of observational studies
**Probability of sexual transmission per partnership**									
Heterosexual transmission (overall)	[26]; [27]; [28]; [29]; [30]; [31]; [32]; [33]; [34]	2946; 251; 528; 304; 266; 101; 406; 701;	"allintitle: HIV AND (infectivity OR transmission OR infection OR infectious OR infectiousness) AND review"	21; 1; 3; 3; 7; 1; 3; 5	[e] 12 [35] [36], [37,38] [38,39] [27,31,40,41] [15] [42] [43]	[f] Calculated rate: 5.35; 6.61; 5.80; 3.6; 5.64	[i] Triangulated the gender and CD4 specific transmission probability based on reviewed evidence and assumptions.	M-F-CD4<200=0.10 (0.05-0.14); M-F-CD4 _200-499=0.05 (0.02-0.07); M-F-CD4≥500=0.03 (0.02-0.07); F-M-CD4<200=0.05 (0.0125-0.14); F-M-CD4 200-499=0.025 (0.005-0.07); F-M-CD4≥500=0.015	Systematic review of observational studies
Heterosexual transmission (Male to female)						[f] Calculated Rate per 100 person-year: 6.30; 4.53; 5.74; 4.36; 7.26			
Heterosexual transmission (Female to male)						[f] Calculated rate estimates: 3.05; 4.60; relative rate (M to F vs F to			

						M): 1.58; 2.07; relative risk: 2.3 95% CI (1.1-4.8).		(0.005-0.07).	
Heterosexual transmission (By CD4 strata)						AIDS vs non-AIDS: 1.06; 4.8; 3.3 95% CI: (1.6-6.8); 3.6.			
Homosexual transmission (overall)					[32,44]; [45]; [46];[29]; [44]	0.075; 0.102 (95% CI: 0.043-0.160); 0.051 (95% CI: 0.022, 0.08); 0.073 (95% CI: 0.018, 0.128).	Range estimated based on multiple studies (0.05-0.10); Point: 0.073 (meta-analysis)	M-M-CD4<200=0.125 (0.05,0.2); M-M-CD4 _200-499=0.065 (0.025,0.10); M-M-CD4≥500=0.045 (0.025,0.10)	Systematic review of observational studies and meta-analysis of prospective cohort studies.
Homosexual transmission (by CD4 strata)						Limited evidence by CD4.	Same as estimating heterosexual transmission probabilities.		
**Acute stage characteristics**									
Duration	[g] [47]; [48,49]; [47] [28,50,51,52] [40] [48,49] [26,47]; [53]	N/A	N/A	N/A	[47]; [48,49]; [50] [40]; [53]	Rakai studies: 87 days; 150 days; 1.7 months (95% credible interval: 0.55-6.8 months); Prospective studies: 52 days; Meta-analysis: 1.5-12 months	Point estimate was determined based on the prospective studies; range was determined based on more recent studies using the Rakai and European studies and rounded to integer.	1.7 months (0.55-6.8)	Prospective studies and systematic review of observational studies.
Transmission rate ratio (acute vs asymptomatic)		N/A	N/A	N/A	[47]; [48,49]; [47] [28,50,51,52] [40] [48,49] [26,47]; [53]	Rakai studies: 26; 11.7; European study: 3.5; Prospective studies: observed an 1.3-2.5 fold of log_{10} viral load increase.	[j] Point estimates triangulated based on the observed increase in viral load and rate ratio associated with viral load increase.	5.3 (0.79-57)	Prospective studies and meta-analysis of retrospective studies
**Reduced probability of unprotected sexual contacts due to HIV diagnosis/awareness**									
Overall	[54], [55], [56], [57]		“allintitle: ‘meta-analysis’”.	1; 19; 1; 5	[58]; [59] ; [60]; [61];	Odds ratios: 0.69 (95% CI: 0.53–0.90); 0.63 (95% CI: 0.54, 0.75); 0.79 (95% CI:	Point estimates based on [61] which is the most relevant; range estimates	68% (59%-76%)	Meta-analysis of observational studies

						0.69, 0.89); Prevalence ratio: 0.53 (95% CI: 0.45, 0.60); 0.68 (95% CI: 0.59, 0.76).	capturing all the 95%CI.		

[a] Snowball search was conducted using Google Scholar.

[b] The articles were considered relevant if they directly reported the estimates on parameters of interest, or if the parameters of interest can be derived based on the contents reported in the papers; the seed paper was included if they were relevant.

[c] Previous studies did not distinguish the condom effectiveness for heterosexual and homosexual sex; as there was no seed paper, we used PubMed Mesh term search strategy.

[d] Evidence on transmission probability by CD4 strata was limited, and we first obtained the evidence on transmission probability overall, and triangulated the CD4 strata-specific estimates based on different evidence sources: review and meta-analysis of needle-stick studies found the transmission probability through needle stick is 0.0041 (95% CI: 0.0017-0.0095) among AIDS patients, and 0.0024 (0.0014-0.004) among all patients; another study of majority sample with AIDS reported 0.003-0.004; observational study on probability of HIV transmission due to deep injuries estimated a transmission probability of 0.023 (0.02-0.07).

[e] Among all the seed papers and citing papers, estimates from a total of 8 populations/study samples in the North American and European settings were extracted, where the primary subtype is HIV-1 B.

[f] Estimates were directly extracted from the selected papers unless noted. Generally, the per-act transmission probability was reported; we calculated the rates of transmission per 100 person-year based on data reported in the papers.

[g] We did not use the snowball search strategy for these parameters. We found a meta-analysis paper, and based on its content, we were aware that two study populations (the Rakai study and the European study) were commonly used for estimating the duration and infectivity of the acute infection (both are retrospective studies); in addition, we identified another two prospective studies based on expert recommendations of the literature.

[h] Transmission probability through needle sharing is larger than transmission through needle stick and smaller than transmission through deep injuries; and transmission probability increases with lower CD4 counts.

[i] Rates for male to female transmission were transformed to probability using p=1-exp(-rt), resulting in transmission probability ranged from 0.043 to 0.070 (those studies did not distinguish HIV stages or CD4); if assuming the relative risk of AIDS vs non-AIDS to be 2; and assuming two extreme scenarios that those studies estimating male to female transmissions included only AIDS patients and only non-AIDS patients, respectively; we can arrived at the estimates ranges for male to female transmission to be: >0.043-<0.140 for CD4<200; and >0.0215-<0.070 for CD4>=200; according to the range, we determined the point estimates as the mid-point of the ranges; female to male transmission probabilities were estimated based on male to female's and the relative risk estimates.

[j] One US prospective study observed an 1.3 fold increase of log10 viral load during the acute stage; literature review[26,47] indicated that transmission rate ratio per log10 viral load increase is 2.09 (95% CI: 1.47, 2.97); therefore, acute stage transmission rate ratio=2.6 (2.09^1.3); another prospective study observed an 2.5 fold increase in viral load; altogether ,the range of this estimate can be determined 1.65 (1.47^1.3) to 15 (2.97^2.5).

[k] Underlined type of evidence indicated the evidence supporting point estimates and range estimates, and the non-underlined evidence supporting the range estimates.

S1 Supplement C: Derivation of initial and dynamic model parameter values for each city

S1 Supplement C describes the process of identifying and selecting evidence, as well as the process of transforming the evidence into parameter inputs for our dynamic transmission model. In this supplement, we draw a distinction between the dimensions of evidence (e.g. the number of different sources, level of stratification, etc.) and the dimensions of model parameters (i.e. the inputs that directly allow us to populate our dynamic transmission model). Our model required estimates for 1667 total parameters; however, the available evidence did not allow us to estimate every parameter value directly. In some cases the dimensions of evidence and model parameters were the same (i.e. a direct 1-to-1 mapping of evidence to parameter values with no additional triangulation or adaptation required), particularly when we were able to estimate parameter values directly from primary data analysis. In other cases, model parameter dimensions were substantially different from those of the evidence and required combining evidence from different sources, along with assumptions. For example, section 4.2.1 details the derivation of the number of individuals on OAT by city, which we calculated from a number of different sources due to a lack of direct and comprehensive surveillance estimates for every city.

Each subsection describes the dimension of model input parameters, including the level of stratification used in the model (i.e. gender, race/ethnicity, etc.) and whether a parameter was city-specific or common across cities (total number of parameters for each city). We also discuss the identification and selection process for evidence sources (outlined in S1 Supplement Table B1), as well as the equations used to derive model input data, when these values were not available directly from reports, literature sources, or estimated directly from primary data analysis. For transparency in our derivation and population of model parameter inputs, plain-language equations in each subsection describe how we derived each model parameter, with subscripts indicating on which subgroups a particular piece of evidence/parameter value is stratified (gender, race/ethnicity, risk group, CD4 cell count among PLHIV).

For example, "$Total\ infected\ PLHIV_{Race/Ethnicity \times Gender \times Risk \times CD4}$" indicates that the parameter values for the total number of infected PLHIV are stratified by race/ethnicity (black/African American, Hispanic/Latino, and non-Hispanic white/others), gender (male/female), risk group (people who inject drugs (PWID), men who have sex with men (MSM), MSM who inject drugs (MWID), heterosexual (HET)), and CD4 cell count (≥ 500 cells/µL, 200-499 cells/µL, < 200 cells/µL)).

We also discuss any assumptions and other information required to generate model parameter values, as well as how we incorporated parameter and evidence uncertainty into our model via ranges. We discuss distributional choices and assumptions for probabilistic sensitivity analysis (PSA), based on parameter data types, in S1 Supplement E.

Tables for evidence sources are organized by section and subsection, and attached in a separate Excel file.

1. Initial population estimates and population dynamics

1.1 Risk-stratified initial population estimates

Initial population estimates capture 15-64 city-level population numbers that were stratified on the basis of gender (male or female), race/ethnicity (black/African American, Hispanic/Latino, and non-Hispanic white/others), and HIV risk behavior type (men who have sex with men (MSM), people who inject drugs (PWID), MSM who inject drugs (MWID), and heterosexual (HET)). MSM, MWID, and HET were further stratified into subgroups based on HIV sexual risk behavior intensity (high vs. low) (discussed in section 2.1.1), and PWID and MWID were categorized based on whether they were receiving opioid agonist treatment (OAT) (discussed in section 4.2.1), resulting in 42 population subgroups. We distributed these 42 subgroups among 19 health states, including HIV-negative, PLHIV who are unaware (3 CD4 cell count strata and acute HIV), HIV-diagnosed (3 CD4 strata and acute HIV), PLHIV on-ART and off-ART (3 CD4 strata each) (PLHIV population discussed in section 1.2). We also included PrEP states for HIV-negative, acute and chronic HIV among infected/unaware PLHIV (3 PrEP strata) (discussed in section 4.3.1). This resulted in 798 initial population values (42 subgroups x 19 health states).

1.1.1 Population aged 15-64

Model input parameters: To derive initial populations for all risk groups in our model, we required total population numbers by city, stratified by race/ethnicity (black/African American, Hispanic/Latino, white/other) and gender (male/female) (6 parameter values).

Identification and selection of evidence: We identified central statistics databases for initial population values, prioritizing population estimates based on geographic representativeness, time-period, and sampling method (S1 Supplement Table B1). For total population numbers, we selected city-level census data from the United States Census Bureau, stratified by ethnicity and gender into 6 subgroups and available for each city in 2011 (S2 Supplement Table 1.1.1) [62].

<u>Derivation of model parameters</u>

Estimates for population numbers in each gender/race/ethnicity subgroup were directly available from cross-tabulated census data and did not require any additional assumptions.

<u>Initial population</u>

$$Population\ total_{Race/Ethnicity \times Gender} = Directly\ estimated\ from\ census\ data$$

1.1.2 PWID population

Model input parameters: We required initial population numbers of PWID by city, stratified by race/ethnicity and gender (6 parameter values).

Identification and selection of evidence: We identified city-specific PWID prevalence estimates and selected literature estimates using national surveillance data from 2007 as the best available evidence (S2 Supplement Tables 1.1.2.1 & 1.1.2.2) [63].

Derivation of model parameters

We derived estimates for PWID population by multiplying ethnicity-stratified total population numbers by gender-weighted, race/ethnicity-specific prevalence estimates for each city. We used prevalence estimates from the most recent available year, and assumed that prevalence rates remained constant to 2011. To derive gender- and race/ethnicity-stratified PWID prevalence estimates, we assumed that gender proportions of PWID were equivalent within race/ethnicity strata.

Total population of PWID within each city

$$PWID\ population_{Race/Ethnicity \times Gender} = Population\ total_{Race/Ethnicity \times Gender} * Prevalence\ PWID_{Race/Ethnicity \times Gender}$$

1.1.3 MSM population

Model input parameters: We required initial population numbers of MSM by city, stratified by race/ethnicity (3 parameter values).

Identification and selection of evidence: We identified literature sources as the best available evidence for MSM population numbers, and selected a study using population-level survey data for county- or CBSA-specific estimates of MSM proportions among males between 2009-2013 (S2 Supplement Table 1.1.3) [64].

Derivation of model parameters

We derived initial MSM population estimates by multiplying total city-level population estimates for males from census data by county- or CBSA-specific MSM proportions. Given large differences in MSM populations between boroughs of New York City and availability of data, we used population-weighted, borough-level MSM prevalence estimates for total MSM in New York City [64]. Furthermore, we assumed that MSM proportions for Staten Island were equivalent to those in Brooklyn for New York City.

Initial MSM population

$$Population\ MSM_{Race/Ethnicity} = Male\ population_{Race/Ethnicity} * \ Proportion\ MSM\ among\ male$$

1.1.4 MWID population

Model input parameters: We required initial population numbers of MWID by city, stratified by race/ethnicity (3 parameter values).

Identification and selection of evidence: We identified national survey data as the best available city-specific evidence for Atlanta, Los Angeles, Miami, New York, and Seattle for both MSM proportions among male PWID populations, and PWID proportions among MSM populations from National HIV Behavioral Surveillance (NHBS) survey data for MSM and PWID cohorts [65]. For Baltimore, we used national NHBS reports (S2 Supplement Tables 1.1.4.1 & 1.1.4.2) [66].

Derivation of model parameters

We derived MWID population estimates by triangulating from two different derivation methods using NHBS survey data (MSM among PWID population, and PWID among MSM population). Our point estimate averaged these two methods of derivation, and we used each method individually as upper and lower range values.

Initial MWID population (method 1)

$$Population\ MWID\ (method\ 1)_{Race/Ethnicity} = \ Population\ male\ PWID_{Race/Ethnicity} * \ Proportion\ MSM\ among\ male\ PWID_{Race/Ethnicity}$$

Initial MWID population (method 2)

$$Population\ MWID\ (method\ 2)_{Race/Ethnicity} = \ Population\ MSM_{Race/Ethnicity} * \ Proportion\ MSM\ among\ PWID_{Race/Ethnicity}$$

Initial MWID population (average of derivation method 1 and method 2)

$$Population\ MWID_{Race/Ethnicity} = \frac{Population\ MWID\ (method\ 1)_{Race/Ethnicity} + \ Population\ MWID\ (method\ 2)_{Race/Ethnicity}}{2}$$

1.1.5 Heterosexual population

Model input parameters: We required initial population numbers of heterosexuals by city, stratified by race/ethnicity and gender, which we derived within the model.

Identification and selection of evidence: For total population numbers, we selected city-level census data from the United States Census Bureau for each city in 2011. Refer to section 1.1.1 for derivation of overall population estimates by city.

Derivation of model parameters

HIV risk groups in the model were mutually exclusive and collectively exhaustive of the entire population, so we derived the initial heterosexual population as the remaining individuals not identified as PWID, MSM or MWID.

Total heterosexual population, aged 15-64:

$$\begin{aligned} Heterosexual\ population_{Race/Ethnicity \times Gender} &= Total\ population_{Race/Ethnicity \times Gender} - MSM\ population_{Race/Ethnicity} \\ &- PWID\ population_{Race/Ethnicity \times Gender} \\ &- MWID\ Population_{Race/Ethnicity} \end{aligned}$$

1.2 Number of PLHIV

The initial number of PLHIV were a subset of the total population numbers derived in Section 1.1. To derive initial population numbers for PLHIV in our model, we used a combination of city-level surveillance data for diagnosed PLHIV, PLHIV on treatment, as well as estimates of the proportion of PLHIV who are aware of their status. We stratified our estimates according to observed CD4 strata proportions, as well as the proportion of individuals with acute HIV.

1.2.1 HIV infected individuals

Model input parameters: We required initial population values for the number of PLHIV who were diagnosed and infected/unaware by city. For each group of diagnosed and infected/unaware, this included 42 stratified parameter values by gender, race/ethnicity and risk group, as well as high/low sexual risk among HET, MSM, MWID, and OAT status among PWID/MWID x 3 CD4 cell count categories (≥ 500 cells/μL, 200-499 cells/μL, and < 200 cells/μL) (252 parameter values). To capture the relative percentage of PLHIV who were infected but unaware at any given time in the model, we also required the proportion of PLHIV who were aware of their status, stratified by gender, race/ethnicity and risk group (18 parameter values).

Identification and selection of evidence: We identified city-level HIV surveillance data to derive the total numbers of diagnosed PLHIV, stratified by risk group, ethnicity and gender. We identified national HIV cohort data for CD4 stratification among diagnosed, and literature estimates for CD4 stratification among infected but unaware PLHIV. We identified surveillance estimates for the proportion of PLHIV who are aware of their status among those infected. We selected city-specific surveillance data for initial population numbers of diagnosed PLHIV, stratified individually by race/ethnicity, gender, risk group and CD4 cell count for 2011 (Described in S1 Supplement E) [67,68,69,70,71,72]. We selected data for the proportion of PLHIV who are aware of their status based on gender and risk group, weighted by state-level estimates from CDC reports of the proportion of PLHIV aware of their status relative to the national average (S2 Supplement Table 1.2.1) [73,74]. For CD4 stratification among PLHIV who were infected but unaware, we used literature estimates (S2 Supplement Table 1.2.3) [75], and among diagnosed PLHIV we used regional HIVRN data (S2 Supplement Table 1.2.4) [76].

Derivation of model parameters

We derived the total number of diagnosed PLHIV directly from surveillance data. We derived the total number of infected PLHIV indirectly, using total diagnosed PLHIV divided by the proportion of PLHIV who are aware of their status. We triangulated the proportion of PLHIV who were aware of their status, for each city, using a weighting for state-level proportion of PLHIV who are aware of their status relative to the national average and applied this weighting to proportions for each gender and risk group. We assumed that proportions were equivalent across race/ethnic groups. We stratified diagnosed PLHIV by CD4 count according to observed proportions in HIVRN data. We derived estimates for CD4 cell count proportions among MSM, stratified by race/ethnicity, and HET, stratified by race/ethnicity and gender. Due to small sample sizes, we combined estimates for PWID and MWID for each HIVRN region under the assumption that CD4 stratification among PWID and MWID were equivalent. Finally, we distributed PLHIV from unknown risk groups equally among all gender, race/ethnicity and CD4 strata for each city [77].

Number of diagnosed PLHIV

$$Total\ diagnosed\ PLHIV_{Race/Ethnicity\,\times\,Gender\,\times\,Risk} = Directly\ available\ from\ city\ surveillance\ data$$

CD4 stratification of diagnosed PLHIV

$$CD4\ stratification\ at\ diagnosis_{Race/Ethnicity\,\times\,Gender\,\times\,Risk\,\times\,CD4} = Directly\ estimated\ from\ HIVRN\ primary\ data\ analysis$$

Total diagnosed PLHIV stratified by CD4

$$Total\ diagnosed\ PLHIV_{Race/Ethnicity \times Gender \times Risk \times CD4} = Total\ diagnosed\ PLHIV_{Race/Ethnicity \times Gender \times Risk} * CD4\ stratification\ at\ diagnosis_{CD4}$$

Total infected/unaware PLHIV

$$Total\ infected\ PLHIV_{Race/Ethnicity \times Gender \times Risk} = \frac{Total\ diagnosed\ PLHIV_{Race/Ethnicity \times Gender \times Risk}}{Proportion\ HIV\ aware}$$

Total infected/unaware PLHIV stratified by CD4

$$Total\ infected/unaware\ PLHIV_{Race/Ethnicity \times Gender \times Risk \times CD4} = (Total\ infected\ PLHIV_{Race/Ethnicity \times Gender \times Risk} - Total\ diagnosed\ PLHIV_{Race/Ethnicity \times Gender \times Risk}) * Proportion\ CD4\ strata\ among\ infected/unaware$$

1.2.2 ART status

Model input parameters: For PLHIV on-ART and off-ART post-initiation, we required 42 stratified parameter values by gender, race/ethnicity and risk group, as well as high/low sexual risk among HET, MSM, MWID, and OAT status among PWID/MWID, all stratified by 3 CD4 cell count categories (≥ 500 cells/µL, 200-499 cells/µL, and < 200 cells/µL) and by city (252 total parameter values). We also required the proportion of PLHIV ever on ART, and currently on ART, by city, stratified by gender, race/ethnicity and risk group (36 total parameter values).

Identification and selection of evidence: We identified city-specific surveillance data as the best available evidence. Given limitations in surveillance data for the proportion of individuals who had ever initiated ART, we used the proportion of individuals linked to care as a proxy for those who had ever initiated ART (S2 Supplement Table C1.2.5) [67,68,69,70,71,72]. We used city-specific surveillance data to estimate the proportion of individuals currently on ART among those who were ever on ART, based on city-level estimates of individuals in receipt of ART in the previous year (S2 Supplement Table C1.2.6) [67,68,69,70,71,72]. We derived CD4 cell count distributions for PLHIV on and off ART from primary analysis of HIVRN data by region using data from 2011-2015 (S2 Supplement Tables C1.2.8 & C1.2.9) [76].

Derivation of model parameters

Given the lack of available evidence, as well as IAS guidelines recommending immediate ART initiation for all infected PLHIV [78], we assumed that the proportion of diagnosed PLHIV ever initiating ART was approximated by the proportion of diagnosed PLHIV who were linked to care. For initial CD4 distributions of diagnosed individuals on and off ART, we derived cross-tabulated proportions directly from HIVRN data using combined estimates from 2011-2015, stratified by race/ethnicity for all risk groups, in addition to gender among HET. We assumed CD4 distribution to be equivalent for male and female PWID.

PLHIV ever on ART

$$\begin{aligned} &Proportion\ of\ PLHIV\ ever\ on\ ART_{Race/Ethnicity \times Gender \times Risk} \\ &\quad = Proportion\ of\ PLHIV\ linked\ to\ care_{Race/Ethnicity \times Gender \times Risk} \\ &\quad = Estimated\ from\ city\ surveillance\ data \end{aligned}$$

PLHIV currently on ART

$$\begin{aligned} &Proportion\ currently\ on\ ART\ among\ ever\ on\ ART_{Race/Ethnicity \times Gender \times Risk} \\ &\quad = Estimated\ from\ city\ surveillance\ data \end{aligned}$$

Initial PLHIV on ART

$$\begin{aligned} &Total\ PLHIV\ on\ ART_{Race/Ethnicity \times Gender \times Risk} \\ &= Proportion\ ever\ on\ ART_{Race/Ethnicity \times Gender \times Risk} \\ &* Proportion\ currently\ on\ ART\ among\ ever\ on\ ART_{Race/Ethnicity \times Gender \times Risk} \\ &* Total\ diagnosed\ PLHIV_{Race/Ethnicity \times Gender \times Risk \times CD4} \end{aligned}$$

Initial PLHIV off-ART post-initiation

$$\begin{aligned} &Total\ PLHIV\ off\ ART_{Race/Ethnicity \times Gender \times Risk} \\ &\quad = Total\ PLHIV\ ever\ on\ ART_{Race/Ethnicity \times Gender \times Risk} \\ &\quad - Total\ PLHIV\ on\ ART_{Race/Ethnicity \times Gender \times Risk} \end{aligned}$$

1.2.3 Proportion of acute HIV

Model input parameters: We required estimates of PLHIV in acute stage HIV, stratified by gender, race/ethnicity, risk group, high/low sexual risk among HET, MSM, MWID and OAT status among PWID/MWID, for infected/unaware and diagnosed/ART naïve (84 parameter values).

Identification and selection of evidence: We identified literature sources as the best available evidence to derive the proportion of PLHIV in acute stage HIV among infected/unaware and diagnosed/ART naïve PLHIV (S2 Supplement Table C1.2.7) [75].

Derivation of model parameters

Given limited evidence, we assumed that the proportion of individuals in acute stage HIV was the same for all infected PLHIV, both infected/unaware and diagnosed/ART naïve. Furthermore, we assumed that proportions of individuals with acute HIV were drawn equally from all strata (race/ethnicity, gender, risk group, CD4 cell count).

Acute state HIV

$$\begin{aligned} &Total\ acute\ HIV\ among\ diagnosed/ART\ naive\ PLHIV_{Gender \times Ethnicity \times Risk \times CD4} \\ &\qquad = Total\ diagnosed/ART\ naive\ PLHIV_{Gender \times Ethnicity \times Risk \times CD4} \\ &\qquad * Proportion\ acute\ HIV\ among\ PLHIV \end{aligned}$$

$$\begin{aligned} &Total\ acute\ HIV\ among\ infected/unaware_{Gender \times Ethnicity \times Risk \times CD4} \\ &\qquad = Infected/unaware\ PLHIV_{Gender \times Ethnicity \times Risk \times CD4} \\ &\qquad * Proportion\ acute\ among\ PLHIV \end{aligned}$$

1.3 Population dynamics

In order to capture population dynamics unique to each city, we calculated entry, maturation and mortality rates for each city. For population dynamics among HIV-negative populations, we calculated net population changes stratified by gender and race/ethnicity based on in- and out-maturation (i.e. individuals who are 14 turning 15, and 64 turning 65) combined with long-term projected growth. We calculated out-maturation rates among PLHIV based on diagnosed PLHIV who are 64 turning 65, based on surveillance data for each city. For mortality rates, we used a combination of published mortality estimates and primary data analysis for PLHIV, as well as population life tables for HIV-negative individuals.

1.3.1 Population entry, growth and maturation rates

Model parameter inputs: We required population growth parameters for HIV-negative individuals (18 parameter values), as well as out-maturation rates for PLHIV (18 parameter values).

Identification and selection of evidence: We identified census data from the United States Census Bureau as the best available evidence, stratified by race/ethnicity and gender [62], as well as population growth projections for each city.

Derivation of model parameters

We derived in-maturation rates based on the population proportion aged 15-19, relative to the total population, and out-maturation rates based on the population proportion aged 60-64, relative to the total population. To ensure that our model matched long-term population growth projections and changing demographics in each city, we used external reports and/or data to adjust total population growth parameters. Projections stratified by age, gender and race/ethnicity, were available for Atlanta [79], Baltimore [80], Los Angeles [81], and Miami [82]. For New York City, race/ethnicity stratified growth projections were not available at the city, county or state level, so we triangulated projections using age-gender stratified estimates combined with national trends for changing race/ethnic population compositions to 2040 [83,84]. For Seattle, stratified growth projections were not available at the city level, so we used population projections for Washington State [85]. We incorporated these projections to ensure that population growth parameters produced projections that matched the overall growth rates from long-term projections accounting for external factors and trends affecting city growth rates. We assumed that population entry and maturation rates were the same across risk groups within race/ethnicity and gender strata (S2 Supplement Tables C1.3.1.1 & C1.3.1.2). PLHIV maturation rates were derived from HIV surveillance data, using the same methods as general population maturation. We distinguished PLHIV maturation rates from those of the general population to reflect the different age structure of PLHIV cohorts (S2 Supplement Table C1.3.2).

$$In\ maturation\ HIV-negative = \frac{(Total\ population\ aged\ 15-19_{Race/Ethnicity \times Gender})/5}{Total\ population_{Race/Ethnicity \times Gender}}$$

$$Out\ maturation\ HIV-negative = \frac{(Total\ population\ aged\ 60-64_{Race/Ethnicity \times Gender})/5}{Total\ population_{Race/Ethnicity \times Gender}}$$

$$Out\ maturation\ PLHIV = \frac{(Total\ PLHIV\ aged\ 60-64_{Race/Ethnicity \times Gender})/5}{Total\ PLHIV_{Race/Ethnicity \times Gender}}$$

$$Population\ growth\ rate\ HIV-negative = Back-calculated\ from\ 2040\ population\ projections$$

$$Population\ entry\ HIV-negative_{Race/Ethnicity \times Gender} = In\ maturation_{Race/Ethnicity \times Gender} + Population\ growth_{Race/Ethnicity \times Gender}$$

$$Net\ population\ growth\ HIV - negative_{Race/Ethnicity \times Gender}$$
$$= Population\ entry\ HIV - negative_{Race/Ethnicity \times Gender}$$
$$- Out\ maturation\ HIV - negative_{Race/Ethnicity \times Gender}$$

1.3.2 Mortality rates

Model parameter inputs: We required monthly mortality rates, stratified by race/ethnicity, gender, risk group, high/low risk among MSM, MWID and HET, as well as OAT status among PWID and MWID (42 subgroups). We stratified mortality estimates among subgroups by HIV-status (HIV-negative, off-ART (3 CD4 cell count categories + acute HIV), on-ART (3 CD4 cell count categories)) (336 parameter values).

Identification and selection of evidence: We selected population life tables for mortality rates of HIV-negative HET and MSM aged 15-64 (S2 Supplement Table C1.3.3.1) [86,87,88,89,90,91], longitudinal cohort data from HIVRN for mortality rates among PLHIV on-ART (S2 Supplement Tables C1.3.3.4, C1.3.3.5, C1.3.3.6) [92], and literature sources for PLHIV off-ART (S2 Supplement Tables C1.3.3.2 & C1.3.3.3) [93]. For mortality rates among HIV-negative PWID we used peer-reviewed literature estimates [94], as well as for PWID/MWID who were on OAT (S2 Supplement Table C1.3.5) [95].

Derivation of model parameters

We assumed equivalent mortality rates for infected/unaware, ART-naïve, and off-ART PLHIV within each gender, race/ethnicity, risk group, CD4 strata. We assumed that mortality rates among high-risk HET, MSM and MWID were equivalent to those of low-risk HET, MSM, and MWID. We assumed that mortality rates for HET and MSM were equivalent between HIV-negative, infected acute HIV and PLHIV with CD4 count ≥ 500 cells/μL, and were based on population life tables. We used standardized mortality ratio multipliers from literature estimates to derive mortality rates for HIV-negative PWID/MWID relative to non-PWID/MWID, as well as all PWID/MWID on OAT relative to off OAT. For PLHIV on-ART, we estimated mortality rates directly from HIVRN data for each stratified subpopulation.

For PLHIV with CD4 200-499 cells/μL and < 200 cells/μL who were off-ART, we used HIVRN data, pooled across all PLHIV groups and cities, as well as literature estimates to derive point estimates and ranges for mortality rates [93,96]. Additionally, for PWID among off-ART PLHIV, we used mortality rate multipliers for those with CD4 200-499 cells/μL and 200 cells/μL with mortality hazard ratios derived from HIVRN data as the point estimates and lower bounds, and literature estimates as upper bounds (S2 Supplement Tables C1.3.4.1 & C1.3.4.2) [97].

Mortality rates for HIV-negative population

$$Baseline\ mortality\ rate_{Race/Ethnicity \times Gender} = Population\ life\ tables_{Race/Ethnicity \times Gender}$$

$$Mortality\ rate\ susceptible\ (PWID) = Literature\ estimates$$

$$Mortality\ rate\ susceptible\ (MWID)_{Race/Ethnicity \times Gender} = \frac{Baseline\ morality\ rate_{Race/Ethnicity \times Gender} + Mortality\ rate\ susceptible\ (PWID)}{2}$$

Mortality rates for PLHIV

$$Mortality\ rate\ PLHIV\ on\ ART_{Race/Ethnicity \times Gender \times Risk \times CD4} = Direct\ estimate\ from\ HIVRN\ primary\ analysis$$

$$Mortality\ rate\ PLHIV\ off\ ART\ (CD4\ 200-499\ cells/\mu L;\ CD4 < 200\ cells/\mu L) = Direct\ estimate\ from\ HIVRN\ analysis$$

Mortality rates for PWID/MWID

$$Mortality\ rate\ PWID/MWID\ off\ OAT\ (CD4\ 200 - 499\ cells/\mu L;\ CD4 < 200\ cells/\mu L)_{Race/Ethnicity \times Gender \times CD4} = Mortality\ rate_{Race/Ethnicity \times Gender \times CD4} * PWID\ mortality\ hazard\ ratio\ (CD4\ 200 - 499\ cells/\mu L;\ CD4 < 200\ cells/\mu L)$$

$$Mortality\ rate\ PWID/MWID\ on\ OAT_{Race/Ethnicity \times Gender \times CD4} = Mortality\ rate\ PWID/MWID\ off\ OAT_{Race/Ethnicity \times Gender \times CD4} * Mortality\ hazard\ ratio\ for\ PWID\ on\ OAT$$

1.4 HIV-negative population

Given that the health states in our model were mutually exclusive and collectively exhaustive, we derived the HIV-negative population from population-level census data as the remaining individuals in each population subgroup after all PLHIV health states had been populated.

1.4.1 Total HIV-negative population

Model input parameters: We required initial population numbers for HIV-negative individuals, stratified by risk group, race/ethnicity and gender, high/low sexual risk among HET, MSM, MWID, and OAT status among PWID/MWID. We derived these numbers within our model, based on the numbers in all other health states.

Identification and selection of evidence: For total HIV-negative population numbers, we selected city-level census data from the United States Census Bureau for each city in 2011 (S2 Supplement Table C1.1.1) [62]. Please refer to section 1.1.1 for derivation of overall population estimates by city.

<u>Derivation of model parameters</u>

We derived initial numbers of HIV-negative individuals in the model as the difference between total population numbers and all infected PLHIV (including both infected/unaware and diagnosed).

<u>Total HIV-negative population</u>

$$\begin{aligned} &Total\ HIV - negative\ population_{Ethnicity \times Gender \times Risk} \\ &\qquad = Total\ population_{Ethnicity \times Gender \times Risk} \\ &\qquad - Total\ infected\ PLHIV_{Ethnicity \times Gender \times Risk} \end{aligned}$$

1.4.2 Screened among HIV-negative

Model input parameters: We required the proportion HIV-negative individuals who had been HIV-screened at baseline, stratified by risk group, race/ethnicity and gender, for each city (42 parameter values). We also required the average duration of time that HIV-negative individuals remained identified after HIV screening.

Identification and selection of evidence: We identified NHBS, Behavioral Risk Factor Surveillance System (BRFSS) and the New York City Community Health Survey (NYC-CHS) data as the best sources of evidence to derive the initial proportion of HIV-negative individuals screened for HIV (S2 Supplement Table C1.4.1) [65].

<u>Derivation of model parameters</u>

We derived testing rates based on the percentage of individuals receiving an HIV test in the past 12 months. We stratified by high/low sexual risk behavior among HET and MSM and we assumed that testing rates for low risk HET, MSM and MWID were equivalent. We used NHBS data to derive testing rates for high risk and BRFSS for low risk, with the exception of the use of the NYC-CHS for testing rates in New York City [98,99]. To derive ranges for NHBS testing rates, we used stratified BRFSS standard error

estimates [94, 95]. We assumed that HIV-negative individuals remained identified for 12-months after screening.

Total screened among HIV-negative population

$$Total\ screened\ among\ HIV-negative_{Race/Ethnicity \times Gender \times Risk} = Total\ HIV-negative\ population_{Race/Ethnicity \times Gender \times Risk} * HIV\ testing\ rate_{Race/Ethnicity \times Gender \times Risk}$$

2. Parameters used to calculate the probability of HIV transmission

2.1 Sexual risk behaviours

To model sexual risk behaviour, we included parameters capturing the distinction between individuals with high and low sexual risk behaviours by stratifying initial population estimates based on the number of sexual partners. We also included parameters for the reduction in sexual partners due to HIV diagnosis and the probability of condom use during same- and opposite-sex sexual encounters.

2.1.1 Stratification by high/low sexual risk behavior

Model input parameters: We required proportions of individuals with high and low risk sexual behavior among infected and HIV-negative MSM/MWID and HET, stratified by gender and race/ethnicity for each city (36 total parameter values).

Identification and selection of evidence: We identified National Survey of Family Growth (NSFG) data as the best source of evidence to derive proportions of high/low risk among HET [100], and NHBS data to derive proportions of high/low risk among MSM/MWID (S2 Supplement Tables C2.1.1.1 & C2.1.1.2) [65]. We supplemented NSFG and NHBS data with primary analysis of data from the AIDS Linked to IntraVenous Experience (ALIVE) PWID cohort study [101], and from Project AWARE to inform ranges used in sensitivity analyses and/or calibration [102].

<u>Derivation of model parameters</u>

We derived the proportion of high risk among HIV-negative HET using the proportion of individuals who had 5 or more sexual partners in the past 12 months. We derived the proportion of high risk among HIV-negative individuals by assumption, based on the percentage of MSM reporting condom-less sex in their most recent encounter with a casual partner [103], and among MSM/MWID PLHIV [104], and HET PLHIV [105], from literature sources. We used proportions of PLHIV individuals with STDs as a proxy for high-risk behavior. We derived proportions of high risk among infected by assuming a constant multiplier on low risk HET and MSM/MWID.

<u>Proportion of high risk among HIV-negative</u>

$$Proportion\ of\ high\ risk\ HIV-negative\ HET\ =\ Direct\ estimate\ from\ NSFG\ data$$

$$Proportion\ of\ high\ risk\ HIV-negative\ MSM/MWID\ =\ By\ assumption$$

<u>Proportion of high risk among PLHIV</u>

$$Proportion\ of\ high\ risk\ infected\ HET = Proportion\ of\ high\ risk\ HIV-negative\ HET * Multiplier\ for\ high\ risk\ infected\ vs.HIV-negative$$

$$Proportion\ of\ high\ risk\ infected\ MSM/MWID\ = Literature\ sources$$

We used 95% confidence intervals derived from NSFG estimates as upper and lower range values.

2.1.2 Number of sexual partners

Model input parameters: We required monthly numbers of same sex partners, stratified by race/ethnicity and risk group for MSM and MWID (18 parameter values). We required monthly numbers of opposite sex partners, stratified by gender, race/ethnicity and risk group (42 parameter values).

Identification and selection of evidence: We identified NSFG data as the best source of evidence for opposite sex partners among HET/PWID, and same- and opposite-sex sexual partners for low risk MSM [100]. We selected NHBS data to derive numbers of same- and opposite-sex partners among high risk MSM and MWID (S2 Supplement Tables C2.1.2.1, C2.1.2.2, C2.1.2.3) [103].

Derivation of model parameters

We assumed that monthly numbers of same and opposite sex sexual partners were equivalent between MSM and MWID, as well as opposite sex partners between PWID and HET.

We used NSFG and NHBS means as point estimates and 95% confidence intervals as ranges around point estimates. As we defined high-risk individuals by higher numbers of sexual partners than low-risk, we constrained the lower bounds of high-risk to be greater or equal to the upper bounds of low-risk individuals for the same race/ethnicity, gender and risk group strata. This ensured that the number of sexual partners for high-risk individuals in calibration and PSA was always greater than the number for low-risk individuals.

2.1.3 Decrease in number of sexual partners due to diagnosis

Model parameter inputs: We required an estimate for the decrease in number of sexual partners due to HIV diagnosis, common across cities (1 parameter value).

Identification and selection of evidence: We identified systematic review/meta-analysis literature sources as the best available evidence for the decrease in sexual partners due to diagnosis (S2 Supplement Table C2.1.3) [61].

Derivation of model parameters

We derived the decrease in sexual partners due to diagnosis from literature estimates (S1 Supplement Table B2). We assumed that the percentage reduction in unprotected sex for PLHIV who were aware vs. unaware estimated in the literature was a suitable approximation of sexual risk behavior for the percentage reduction in sexual partners due to diagnosis. We further assumed that the proportional reduction in the number of sexual partners due to diagnosis was constant across gender, race/ethnicity and risk groups.

$$Percentage\ reduction\ in\ number\ of\ sexual\ partners\ due\ to\ diagnosis$$
$$= Percentage\ reduction\ in\ unprotected\ sex\ for\ PLHIV\ aware\ vs.unaware\ of\ status$$

We derived range estimates from 95% confidence intervals in literature sources.

2.1.4 Probability of condom use

Model input parameters: We required estimates of condom use probability for heterosexual sex and homosexual sex, stratified by gender and race/ethnicity, as well as by all risk groups for heterosexual sex and MSM/MWID for homosexual sex (60 parameter values).

Identification and selection of evidence: We identified NSFG data as the best available evidence source for condom use probabilities for heterosexual sex among HET and PWID [100], and NHBS data for condom use probabilities for heterosexual and homosexual sex among MSM and MWID (S2 Supplement Tables C2.1.4.1 & C2.1.4.2) [103].

Derivation of model parameters

We assumed that condom use probabilities were equivalent among low-risk HET, low-risk MSM/MWID and PWID, by race/ethnicity and gender. For high-risk opposite- and same-sex MSM/MWID, we assumed that probabilities were equivalent among MSM and MWID. We used regional data from NSFG to derive estimates for each city, based on census regions.

Condom use probability for heterosexual sex

$$Condom\ use\ probability\ heterosexual\ sex\ among\ HET/PWID_{Race/Ethnicity \times Gender}$$
$$= Directly\ estimated\ from\ NSFG\ primary\ analysis$$

$$Condom\ use\ probability\ heterosexual\ sex\ among\ MSM/MWID_{Race/Ethnicity} = Directly\ estimated\ from\ NHBS\ primary\ analysis$$

We used 95% confidence intervals derived from NSFG and NHBS estimates as upper and lower range values.

2.2 Injection risk behaviours

To model injection risk behaviour, we included parameters capturing the monthly number of injections for PWID/MWID, proportion of shared injections, as well as the effect of HIV diagnosis on injection sharing.

2.2.1 Number of injections

Model parameter inputs: We required an estimate for the monthly number of injections for PWID/MWID, for each city (1 parameter value).

Identification and selection of evidence: We identified peer-reviewed modeling studies as the best available evidence for monthly injections (S2 Supplement Table C2.2.1) [94].

<u>Derivation of model parameters</u>

We assumed that number of injections was equivalent across gender, race/ethnicity and between PWID and MWID.

$$Number\ of\ injections\ for\ PWID/MWID = Literature\ estimates$$

2.2.2 Proportion of shared injections

Model parameter inputs: We required an estimate for the proportion of shared injections for MWID, stratified by race/ethnicity, and PWID, stratified by gender and race/ethnicity for each city (9 parameter values).

Identification and selection of evidence: We identified NHBS data as the best available evidence to derive estimates for the proportion of injections that are shared in Atlanta, Los Angeles, Miami, New York City, and Seattle [106], and we used New York City estimates by assumption for Baltimore, as city-specific NHBS data were unavailable (S2 Supplement Table C2.2.2).

<u>Derivation of model parameters</u>

We derived the proportion of shared injections, based on the proportion of PWID and MWID who reported sharing injections in the past 12 months. We assumed that the

proportion of shared injections was equivalent across gender and between PWID and MWID.

$$Proportion\ of\ injections\ that\ are\ shared_{Race/Ethnicity} = Directly\ estimated\ from\ NHBS\ data_{Race/Ethnicity}$$

We used 95% confidence intervals derived from NHBS proportions as upper and lower range estimates.

2.2.3 Decrease in number of shared injections due to diagnosis

Model parameter inputs: We required an estimate for the decrease in shared injections for PWID/MWID due to diagnosis, common across cities (1 parameter value).

Identification and selection of evidence: We identified NHBS data as the best available evidence to derive estimates for the decrease in shared injections due to HIV diagnosis (S2 Supplement Table C2.2.3) [106].

<u>Derivation of model parameters</u>

To estimate the percentage reduction in shared injections, we calculated the reduced probability for distributive injection sharing after diagnosis. We assumed that the percentage reduction in shared injections was equivalent across gender, race/ethnicity and between PWID and MWID.

$$Percentage\ reduction\ in\ shared\ injections = Directly\ estimated\ from\ NHBS\ data$$

We used 95% confidence intervals derived from NHBS proportions as upper and lower range estimates.

2.3 Sexual mixing patterns

We explicitly modeled sexual mixing patterns within and between race/ethnicity groups, to capture the dynamics of HIV infection among race/ethnicity groups in a particular city, given differences in race/ethnicity composition by city.

Model input parameters: We required assortative sexual mixing parameters by city, for the proportion of sexual partners of the same race/ethnicity for both HET/PWID and MSM/MWID, stratified by race/ethnicity (6 parameter values).

Identification and selection of evidence: We identified NSFG data as the best available evidence for assortative sexual mixing among HET/PWID, and literature estimates for

assortative sexual mixing among MSM/MWID [107,108] (S2 Supplement Tables C2.3.1 & C2.3.2).

Derivation of model parameters

We based our estimates of assortative sexual mixing among HET and PWID on the assortative mixing among high and low risk opposite-sex encounters among individuals in the NSFG. We used literature sources for same-sex assortative mixing among MSM and MWID, using estimates from a Houston-based cohort study for Atlanta, Baltimore, Miami and New York City [107], and estimates from a San Francisco cohort study for Los Angeles and Seattle [108]. We assumed the same mixing patterns for HET and PWID, as well as high/low risk among MSM and MWID.

Heterosexual assortative mixing

$$Assortative\ sexual\ mixing\ HET/PWID_{Race/Ethnicity} = Directly\ estimated\ from\ NSFG$$

Homosexual assortative mixing

$$Assortative\ sexual\ mixing\ MSM/MWID_{Race/Ethnicity} = Directly\ estimated\ from\ literature\ sources$$

We used 95% confidence intervals derived in primary analysis of NSFG for upper and lower range estimates among HET and PWID, and 95% confidence intervals from literature estimates for MSM and MWID.

2.4 Probability of transmission

To model the probability of HIV transmission, we included parameters capturing the baseline probabilities of transmission via sexual contact and injection (modeled as probabilities of transmission per sexual act or shared injection), and the protective effects of ART and condom use in reducing transmission.

2.4.1 Probability of transmission from sexual contact

Model input parameters: We required estimates for the probability of HIV transmission per heterosexual and homosexual sexual contact, common across cities, race/ethnicity and risk group. Heterosexual estimates were further stratified by whether the contact was male-to-female or female to male, and CD4 cell count/acute HIV (12 parameter values).

Identification and selection of evidence: We identified literature sources as the best available evidence sources to derive model parameters for heterosexual sex (male-to-

female), heterosexual sex (female-to-male), homosexual sex, and a multiplier for probability of transmission for PLHIV with acute HIV (S2 Supplement Tables C2.4.1.1, C2.4.1.2, C2.4.1.3, C2.4.1.4) [15,27,31,35,36,37,38,39,40,41,42,43].

Derivation of model input parameters

We derived input parameters for the probability of transmission via sexual contact by triangulating data from different literature sources (Table B2). We used a multiplier to derive the probability of transmission for individuals with acute HIV, relative to chronic HIV with CD4 < 200 cells/µL.

$$Probability\ of\ transmission\ from\ heterosexual\ sex\ (male\ to\ female)_{CD4} = Synthesized\ from\ literature\ estimates$$

$$Probability\ of\ transmission\ from\ heterosexual\ sex\ (female\ to\ male)_{CD4} = Synthesized\ from\ literature\ estimates$$

$$Probability\ of\ transmission\ from\ homosexual\ sex_{CD4} = Synthesized\ from\ literature\ estimates$$

$$Probability\ of\ transmission\ from\ heterosexual/homosexual\ sex\ (acute\ HIV) = Probability\ of\ transmission\ from\ heterosexual/homosexual\ sex\ (CD4 < 200\ cells/\mu L) * Multiplier\ for\ increased\ infectivity\ in\ acute\ HIV\ stage$$

We derived ranges based on the range of 95% confidence intervals in literature estimates.

2.4.2 Probability of transmission from shared injection

Model input parameters: We required estimates for the probability of HIV transmission per injection, common across cities, race/ethnicity, risk group and stratified by CD4 category/acute HIV (4 parameter values).

Identification and selection of evidence: We identified systematic literature reviews of observational studies as the best available evidence to derive parameter estimates for the probability of transmission from shared injection (S2 Supplement Table C2.4.2) [21,23,24,25].

Derivation of model parameters

We derived input parameters for the probability of transmission from injection by triangulating data from different literature sources (S1 Supplement Table B2).

$$Probability\ of\ transmission\ from\ shared\ injection = Synthesized\ from\ literature\ estimates$$

We derived ranges based on the range of 95% confidence intervals from literature estimates.

2.4.3 ART effectiveness on probability of transmission from sexual contact

Model input parameters: We required estimates of ART effectiveness for transmission via heterosexual and homosexual sex, common across cities, gender, race/ethnicity, and risk groups (all risk groups for heterosexual sex and MSM/MWID for homosexual sex) (2 parameter values).

Identification and selection of evidence: We identified systematic reviews/meta-analyses of multiple literature sources as the best available evidence sources to estimate parameter value inputs for heterosexual sex [14,15], and homosexual sex [16,17] (S2 Supplement Table C2.4.3).

Derivation of model parameters

We derived model parameter values for ART effectiveness for transmission via both heterosexual and homosexual contact based on a large meta-analysis study of ART effectiveness [15] (S1 Supplement Table B2).

ART effectiveness heterosexual

$$Percentage\ reduction\ in\ probability\ of\ transmission\ from\ ART\ (heterosexual) = Synthesized\ from\ literature\ estimates$$

ART effectiveness homosexual

$$Percentage\ reduction\ in\ probability\ of\ transmission\ from\ ART\ (homosexual) = Synthesized\ from\ literature\ estimates$$

We derived ranges based on the range of 95% confidence intervals from literature estimates.

2.4.4 ART effectiveness on probability of transmission from injection

Model input parameters: We required estimates of ART effectiveness for transmission via injection, common across cities, gender, race/ethnicity, and PWID/MWID (1 parameter value).

Identification and selection of evidence: We identified systematic reviews/meta-analyses of literature estimates as the best available evidence to derive parameter value estimates (S2 Supplement Table C2.4.4) [18,19,20].

Derivation of model parameters

We derived model parameter values by synthesizing literature estimates and ranges (S1 Supplement Table B2).

ART effectiveness injection

$$Percentage\ reduction\ in\ probability\ of\ transmission\ from\ ART\ (injection) = Synthesized\ from\ literature\ estimates$$

We derived ranges based on the range of 95% confidence intervals from literature estimates.

2.4.5 Condom effectiveness on probability of transmission

Model input parameters: We required estimates of condom effectiveness for heterosexual and homosexual sex, common across cities, gender, race/ethnicity, and risk groups (all risk groups for heterosexual sex and MSM/MWID for homosexual sex) (2 parameter values).

Identification and selection of evidence: We identified systematic reviews/meta-analyses of multiple literature sources as the best available evidence to derive parameter value estimates (S2 Supplement Tables C2.4.5.1 & C2.4.5.2) [10,11,12,13].

Derivation of model parameters

We derived model parameter values by synthesizing literature estimates and ranges (S1 Supplement Table B2).

Condom effectiveness heterosexual

$$Percentage\ reduction\ in\ probability\ of\ transmission\ from\ condom\ use\ (heterosexual) = Synthesized\ from\ literature\ estimates$$

Condom effectiveness homosexual

$$Percentage\ reduction\ in\ probability\ of\ transmission\ from\ condom\ use\ (homosexual) = Synthesized\ from\ literature\ estimates$$

We derived ranges based on the range of 95% confidence intervals from literature estimates.

3. Screening, diagnosis, treatment and HIV disease progression

3.1 HIV Testing

To model HIV testing, we included model parameters capture the monthly rates at which infected/unaware PLHIV are diagnosed from HIV-symptom-based case finding (for infected PLHIV with CD4 cell counts < 500 cells/µL), as well as HIV testing rates for HIV-negative and PLHIV who are unaware.

3.1.1 Symptom-based case finding rate

Model input parameters: We required symptom-based case finding rates for infected/unaware PLHIV (CD4 cell counts < 200 cells/µL; 200-499 cells/µL), common across cities, gender, race/ethnicity and risk group (2 parameter values).

Identification and selection of evidence: We identified cohort studies as the best available source of evidence to inform estimates of symptom-based case finding rates [109,110].

<u>Derivation of model parameters</u>

We derived symptom-based case-finding rates for infected/unaware PLHIV with CD4 cell counts of 200-499 cells/µL and ≥ 500 cells/µL from literature estimates, assuming an equal rate across cities, gender, race/ethnicity and risk group.

3.1.2 HIV testing rates

Model input parameters: We required city-specific HIV testing rates, stratified by gender, race/ethnicity, risk group, as well as high/low sexual risk among MSM, MWID and HET and OAT status among PWID/MWID (42 parameter values).

Identification and selection of evidence: We identified NHBS, Behavioral Risk Factor Surveillance System (BRFSS) and the New York City Community Health Survey (NYC-CHS) data as the best available source of evidence to derive testing rates [65,106,111].

<u>Derivation of model parameters</u>

We derived testing rates based on the percentage of individuals receiving an HIV test in the past 12 months. We stratified by high/low sexual risk behavior among HET and MSM and we assumed that testing rates for low risk HET, MSM and MWID were equivalent. We used NHBS data to derive testing rates for high risk and BRFSS for low risk, with the exception of the use of the NYC-CHS for testing rates in New York City [98,99]. To derive ranges for NHBS testing rates, we used stratified BRFSS standard error

estimates [94, 95]. We assumed that HIV-negative individuals remained identified for 12-months after screening (S2 Supplement Table C3.1.2.1).

We derived testing rates based on the percentage of individuals receiving an HIV test in the past 12 months (yearly probability of HIV test), which we converted to a monthly rate using the formula below. We stratified testing rates by high/low risk sexual behavior among HET and MSM. We assumed that testing rates for low risk HET and MSM were equivalent, as well as testing rates for PWID and MWID (S2 Supplement Table C3.1.2.1). To derive ranges for NYC testing rates, we used additional evidence in estimates from the CDC's Behavioral Risk Factor Surveillance System (BRFSS) and the New York City Community Health Survey (NYC-CHS)

HIV testing rates

$$Monthly\ HIV\ testing\ rate_{Race/Ethnicity \times Gender \times Risk} = -\frac{1}{12} * ln(1 - Yearly\ probability\ of\ HIV\ test)$$

3.2 ART initiation

To model ART initiation, we included parameters for the proportion of diagnosed PLHIV initiating ART within 30 days of diagnosis, as well as the monthly rate of previously diagnosed PLHIV (more than 30 days past diagnosis) initiating ART. The combination of these two parameters allowed us to calculate the total number of PLHIV initiating ART every month.

Model input parameters: We required gender, race/ethnicity and risk-group-stratified proportions, for PLHIV initiating ART immediately upon diagnosis (< 30 days post-diagnosis) (42 parameter values), and individuals initiating ART ≥ 30 days post-diagnosis each month (42 parameter values).

Identification and selection of evidence: We identified city-level surveillance data as the best available evidence for Baltimore, Los Angeles, Miami, New York City and Seattle, state-level surveillance data for Atlanta, to estimate the proportions of individuals who were linked to HIV care (S2 Supplement Table C3.2.1) [67,68,69,70,71,72]. We used regional HIVRN data from 2011-2015 to estimate the proportion of PLHIV who initiated ART immediately upon diagnosis (within 30 days) (S2 Supplement Tables C3.2.2.1, C3.2.2.2, C3.2.2.3), as well as the monthly rate of individuals initiating ART more than 30-days post-diagnosis (S2 Supplement Tables C3.2.3.1, C3.2.3.2, C3.2.3.3) [92]. We supplemented ART initiation rates from HIVRN primary analysis with MMP data [112].

Derivation of model parameters

We calculated the number of PLHIV initiating ART, using the proportion linked to care as the denominator. To derive monthly ART initiation rates for individuals more than 30-days post-diagnosis, we divided the number of PLHIV initiating ART post-30-days, by the total number of follow-up months for PLHIV diagnosed but not initiated ART (PLHIV initiating ART more than 30-days post-diagnosis/month).

ART initiation

$$Proportion\ of\ PLHIV\ linked\ to\ care_{Race/Ethnicity\times Gender\times Risk\times CD4} = HIV\ surveillance\ reports$$

$$Proportion\ PLHIV\ initiating\ ART\ within\ 30\ days\ of\ diagnosis_{Race/Ethnicity\times Gender\times Risk\times CD4} = Direct\ estimate\ from\ HIVRN\ primary\ analysis$$

$$Monthly\ rate\ of\ individuals\ initiating\ ART \geq 30\ days\ post-diagnosis_{Race/Ethnicity\times Gender\times Risk\times CD4} = Direct\ estimate\ from\ HIVRN\ primary\ analysis$$

3.3 ART retention and re-initiation

To model ART retention and re-initiation, we included parameters for the monthly transition probability for PLHIV on ART to dropout stratified by CD4 cell count, as well as the monthly transition probabilities for PLHIV re-initiating ART, not stratified by CD4 cell count.

3.3.1 ART retention

Model input parameters: We required ART dropout rates by CD4 category, stratified by gender, ethnicity and risk group (54 parameter values).

Identification and selection of evidence: We identified longitudinal HIV cohort data from HIVRN as the best available evidence to estimate monthly transition rates for PLHIV on-ART to off-ART (S2 Supplement Tables 3.3.1.1, C3.3.1.2, C3.3.1.3) [92].

Derivation of model parameters

We estimated the probabilities of ART-dropout by simultaneously estimating the transition probability between CD4 strata, ART dropout probability, and ART re-initiation probability using a continuous-time multi-state Markov model. Full details on estimation of disease progression are described elsewhere [113].

ART dropout

$$ART\ dropout\ rate\ CD4\ \geq 500\ cells/\mu L_{Race/Ethnicity \times Gender \times Risk} = Directly\ estimated\ from\ HIVRN\ primary\ analysis$$

$$ART\ dropout\ rate\ CD4\ 200 - 499\ cells/\mu L_{Race/Ethnicity \times Gender \times Risk} = Directly\ estimated\ from\ HIVRN\ primary\ analysis$$

$$ART\ dropout\ rate\ CD4\ < 200\ cells/\mu L_{Race/Ethnicity \times Gender \times Risk} = Directly\ estimated\ from\ HIVRN\ primary\ analysis$$

3.3.2 ART re-initiation

Model input parameters: We required gender, ethnicity and risk-group-stratified rates for individuals re-initiating ART after interruptions in treatment (18 parameter values).

Identification and selection of evidence: We identified longitudinal HIV cohort data from HIVRN as the best available evidence to estimate monthly transition rates for PLHIV on ART (S2 Supplement Table C3.3.2.1) [92].

Derivation of model parameters

We assumed that ART re-initiation rates were equivalent across CD4 cell categories. We estimated ART re-initiation probabilities by simultaneously estimating the transition probability between CD4 strata, ART dropout probability, and ART re-initiation probability using a continuous-time multi-state Markov model. Full details on estimation of disease progression are described elsewhere [113].

ART re-initiation

$$ART\ re-initiation\ rate_{Race/Ethnicity \times Gender \times Risk} = Directly\ estimated\ from\ HIVRN\ primary\ analysis$$

3.4 HIV disease progression on ART

To model HIV disease progression for PLHIV on ART, we included monthly probabilities for transition between CD4 cell count categories. PLHIV on ART could transition between any of the three CD4 cell count categories.

3.4.1 Disease progression for PLHIV on ART

Model input parameters: We required monthly transition probabilities between CD4 cell count categories, stratified by gender, race/ethnicity and risk group for PLHIV on ART (108 parameter values).

Identification and selection of evidence: We identified longitudinal HIV cohort data from HIVRN as the best available evidence to estimate monthly disease progression transition rates for PLHIV on ART (S2 Supplement Tables C3.4.1.1, C3.4.1.2, C3.4.1.3, C3.4.1.4, C3.4.1.5, C3.4.1.6) [92].

Derivation of model parameters

We estimated disease progression on ART by simultaneously estimating the transition probability between CD4 strata, ART dropout probability, and ART re-initiation probability using a continuous-time multi-state Markov model. Full details on estimation of disease progression are described elsewhere [113].

HIV disease progression on ART

$$Transition\ probability\ from \geq 500\ cells/\mu L\ to\ 200 - 499\ cells/\mu L\ for\ PLHIV\ on\ ART_{Race/Ethnicity \times Gender \times Risk} = Directly\ estimated\ from\ HIVRN\ primary\ analysis$$

$$Transition\ probability\ from \geq 500\ cells/\mu L\ to < 200\ cells/\mu L\ for\ PLHIV\ on\ ART_{Race/Ethnicity \times Gender \times Risk} = Directly\ estimated\ from\ HIVRN\ primary\ analysis$$

$$Transition\ probability\ from\ 200 - 499\ cells/\mu L\ to < 200\ cells/\mu L\ for\ PLHIV\ on\ ART_{Race/Ethnicity \times Gender \times Risk} = Directly\ estimated\ from\ HIVRN\ primary\ analysis$$

$$Transition\ probability\ from\ 200 - 499\ cells/\mu L\ to \geq 500\ cells/\mu L\ for\ PLHIV\ on\ ART_{Race/Ethnicity \times Gender \times Risk} = Directly\ estimated\ from\ HIVRN\ primary\ analysis$$

$$Transition\ probability\ from < 200\ cells/\mu L\ to\ 200 - 499\ cells/\mu L\ for\ PLHIV\ on\ ART_{Race/Ethnicity \times Gender \times Risk} = Directly\ estimated\ from\ HIVRN\ primary\ analysis$$

$$Transition\ probability\ from\ <\ 200\ cells/\mu L\ to\ \geq 500\ cells/\mu L\ for\ PLHIV\ on\ ART_{Race/Ethnicity\times Gender\times Risk} = Directly\ estimated\ from\ HIVRN\ primary\ analysis$$

We derived ranges from 95% confidence intervals generated in primary analysis of HIVRN data.

3.5 HIV disease progression off ART

To model HIV disease progression off ART, we included monthly transition probabilities for transition between CD4 cell count categories for PLHIV off ART.

3.5.1 Disease progression for infected and diagnosed PLHIV

Model input parameters: We required transition probabilities from acute to chronic HIV for infected/unaware and diagnosed PLHIV, common across city, gender, race/ethnicity, and risk group (2 parameter values).

Identification and selection of evidence: We identified longitudinal cohort studies as the best available evidence for estimates of disease progression off ART (S2 Supplement Tables C3.5.1.1 & C3.5.1.2) [109,110].

Derivation of model parameters

We derived our estimates based on disease progression from ≥ 200 cells/μL to 200-499 cells/μL using the transition rate from HIV to AIDS observed from events per 100 person-years [110]. We assumed that monthly disease progression rates from ≥ 500 cells/μL to 200-499 cells/μL and from 200-499 cells/μL to < 200 cells/μL for infected/unaware PLHIV were equivalent. In the absence of treatment, we assumed that PLHIV progressed from higher to lower CD4 cell counts while off ART.

HIV disease progression for infected and diagnosed

$$Transition\ probability\ from\ CD4 \geq 500\ cells/\mu L\ to\ CD4\ 200-499\ cells/\mu L\ for\ infected\ and\ diagnosed\ PLHIV = Synthesized\ from\ literature\ estimates$$

$$Transition\ probability\ from\ CD4\ 200-499\ cells/\mu L\ to\ CD4 < 200\ cells/\mu L\ for\ infected\ and\ diagnosed\ PLHIV = Synthesized\ from\ literature\ estimates$$

3.5.2 Transition from acute to chronic states for infected and diagnosed

Model input parameters: We required transition rates from acute to chronic HIV for infected/unaware and diagnosed PLHIV, common across city, gender, race/ethnicity, and risk group (2 parameter values).

Identification and selection of evidence: We identified longitudinal cohort studies as the best available evidence to estimate transitions from acute to chronic HIV states for infected/unaware and diagnosed PLHIV (S2 Supplement Tables C3.5.2.1 & C3.5.2.2) [50].

Derivation of model parameters

We derived the transition from acute to chronic HIV by converting literature estimates for the duration that PLHIV spend in acute HIV into a monthly rate. We derived transitions from acute to chronic HIV for diagnosed PLHIV based on assumptions relative to infected/unaware PLHIV.

HIV disease progression from acute to chronic HIV

$$Transition\ rate\ from\ acute\ to\ chronic\ HIV\ for\ infected/unaware\ PLHIV = \frac{1}{Monthly\ duration\ of\ acute\ HIV\ state}$$

$$Transition\ rate\ from\ acute\ to\ chronic\ HIV\ for\ diagnosed\ PLHIV = \frac{1}{Monthly\ duration\ of\ acute\ HIV\ state}$$

4. HIV prevention programs

To model the efficacy of HIV prevention programs, we included model parameters capturing total syringe distribution volumes from syringe services programs (SSP) and OAT coverage among PWID/MWID by city, as well as time-varying estimates of pre-exposure prophylaxis (PrEP) coverage among HIV-negative MSM/MWID.

4.1.1 SSP coverage

We derived SSP coverage parameters for cities in our model based on the total volume of syringe distribution, divided by the PWID/MWID population.

Model input parameters: To calculate SSP coverage, we required total SSP volume estimates by city (1 parameter value).

Identification and selection of evidence: We identified the best available evidence for Atlanta based on estimates from the Atlanta Harm Reduction Coalition in 2016 [114]. Estimates for Baltimore were based on the City of Baltimore Syringe Exchange Program in 2016 [115]. Estimates for Los Angeles were based on direct correspondence with the City of Los Angeles AIDS Coordinator's Office for Los Angeles [116]. Estimates for Miami were based on national CDC estimates, as local surveillance estimates were not available [117]. Estimates for New York City were based on New York state department of health reports in 2012 [118]. Estimates for Seattle were based on direct correspondence with Public Health – Seattle & King County for Seattle (Supplement Table C4.1) [119].

Derivation of model parameters

We assumed that syringes were distributed equally according to the gender and race/ethnicity proportions in the underlying population of PWID/MWID.

SSP distribution volume

$$Syringe\ distribution\ volume = \ Estimates\ from\ city\ and\ national\ reports$$

We derived lower ranges for Atlanta and Miami based on assumptions, and upper ranges based on national urban SSP averages [117]. We derived SSP low ranges for NYC from 2006 estimates, and high ranges by extrapolating SSP volume trends to 2016.

4.1.2 SSP effectiveness for reducing shared injections

Model input parameters: We required a multiplier for reduction in shared injections due to SSP for PWID and MWID, common across cities (1 parameter value).

Identification and selection of evidence: We identified meta-analyses of observational studies as the best source for estimating the effectiveness of SSP in reducing shared injections (S2 Supplement Table C4.1.2) [120].

Derivation of model parameters

Given the lack of direct evidence for the reduction in shared injections due to SSP, we derived our parameter estimate based on the pooled effect size of SSP in reducing HIV transmission for PWID/MWID. Two pooled effect sizes were reported in the source study (one analysed from all included studies and one only across the subset of higher quality studies) and we derived the parameter based on the latter estimate.

$$Multiplier\ for\ shared\ injections\ covered\ by\ SSP \\ = (1 - Effect\ size\ of\ SSP\ in\ reducing\ HIV\ transmission)$$

We used 95% confidence intervals from literature estimates to derive high and low ranges for the reduction in shared injections due to SSP.

4.2 Opioid agonist treatment

To model opioid agonist treatment (OAT), we included parameters capturing the initial stratification of PWID/MWID on- and off-OAT. We also included monthly rates for OAT retention, and the efficacy of OAT in improving adherence to ART and reducing shared injections.

4.2.1 Number of individuals receiving OAT

Model input parameters: We required numbers of PWID and MWID receiving OAT, stratified by race/ethnicity for PWID and MWID, as well as gender for PWID (9 parameter values).

Identification and selection of evidence: We identified administrative databases and surveillance data as the best available evidence for the number of PWID/MWID on OAT. We used TEDS data to estimate the number of individuals receiving methadone in 2010 (S2 Supplement Table C4.2.1) [121]. We estimated the number of individuals receiving Buprenorphine using the DATA waivered physician capacity for each city, and the proportion of statewide DATA waivered physicians in each city (S2 Supplement Table C4.2.2) [122], and the proportion of DATA-waivered physicians accepting Medicaid, which we used to estimate the proportion of PWID patients among physicians' total capacity (S2 Supplement Tables C4.2.5.1 & C4.2.5.2) [123], stratified by gender and race/ethnicity (S2 Supplement Tables C4.2.3 & C4.2.4) [121,124]. Given the lack of evidence for the number in individuals receiving methadone in Atlanta and Miami, we

used the midpoint of the upper and lower range as a point estimate and assumed a lower bound of ‘1’.

<u>Derivation of model parameters</u>

<u>Number of PWID receiving OAT in OTPs</u>

$$PWID\ receiving\ OAT\ in\ OTPs_{Race/Ethnicity\,\times\,Gender} = Direct\ estimate\ from\ TEDS$$

<u>Number of PWID receiving OAT with Buprenorphine</u>

$$DATA\ waivered\ physician\ capacity = SAMHSA$$

$$\begin{aligned} &Proportion\ of\ DATA\ waivered\ physicians\ by\ city \\ &\qquad = Proportion\ of\ statewide\ DATA\ waivered\ physicians\ in\ city \end{aligned}$$

$$\begin{aligned} &Proportion\ of\ PWID\ among\ DATA\ waivered\ physician\ patient\ capacity \\ &\qquad = Proportion\ of\ physicians\ accepting\ Medicaid \end{aligned}$$

$$\begin{aligned} &PWID\ receiving\ OAT\ from\ DATA\ waivered\ physicians \\ &\qquad = DATA\ waivered\ physician\ capacity \\ &\qquad * Proportion\ of\ DATA\ waivered\ physicians\ by\ city \\ &\qquad * Proportion\ of\ PWID\ among\ DATA\ waivered\ physician\ patient\ capacity \end{aligned}$$

$$\begin{aligned} &PWID\ receiving\ OAT\ with\ Buprenorphine_{Ethnicity\,\times\,Gender} \\ &\qquad = Proportion\ gender\ PWID * Proportion\ ethnicity\ PWID \\ &\qquad * PWID\ receiving\ OAT\ from\ DATA\ waivered\ physicians \end{aligned}$$

<u>Total PWID receiving OAT</u>

$$\begin{aligned} &PWID\ receiving\ OAT_{Race/Ethnicity\,\times\,Gender} \\ &\qquad = PWID\ receiving\ OAT\ in\ OTPs_{Race/Ethnicity\,\times\,Gender} \\ &\qquad + PWID\ receiving\ OAT\ with\ Buprenorphine_{Race/Ethnicity\,\times\,Gender} \end{aligned}$$

We derived ranges for PWID on OAT based on the variation in proportion of PWID among DATA-waivered physician patients.

4.2.2 OAT entry and dropout

Model input parameters: We required OAT dropout rates for PWID and MWID, common across cities, gender and race/ethnicity (1 parameter value).

Identification and selection of evidence: We identified systematic reviews of cohort studies as the best available evidence to estimate OAT retention (S2 Supplement Table C4.2.6) [125].

Derivation of model parameters

We derived OAT dropout rates for PWID/MWID as the midpoint between the lowest and highest observed dropout rates among cohort studies in the systematic review.

OAT dropout rate

$$OAT\ dropout\ rate\ =\ Midpoint\ between\ lowest\ and\ highest\ observed\ OAT\ dropout\ rate$$

We derived ranges using the highest and lowest observed OAT dropout rates among studies used to derive the midpoint estimate.

4.2.3 OAT effectiveness for adherence to ART

Model input parameters: We required a multiplier for increased retention in ART due to OAT for PWID and MWID, common across cities (1 parameter value).

Identification and selection of evidence: We identified cohort-based observational studies as the best source for estimating the effectiveness of OAT in improving ART retention (S2 Supplement Table C4.2.7) [126].

Derivation of model parameters

We derived the multiplier for OAT effectiveness on ART dropout rates using odds-ratios for the effect of OAT on reducing ART dropout rates.

$$OAT\ multiplier\ for\ ART\ dropout\ rate =\ Odds\ ratio\ for\ reduced\ ART\ dropout\ on\ OAT$$

We used 95% confidence intervals from literature estimates to derive upper and lower ranges for the reduction in ART dropout due to OAT.

4.2.4 OAT effectiveness for reducing shared injections

Model input parameters: We required a multiplier for proportional reduction in shared injections due to OAT for PWID and MWID, common across cities (1 parameter value).

Identification and selection of evidence: We identified meta-analyses of cohort studies as the best source for estimating the effectiveness of OAT in reducing shared injections (S2 Supplement Table C4.2.8) [127].

Derivation of model parameters

Given the lack of direct evidence for the reduction in shared injections due to OAT, we derived our parameter estimate based on the rate ratio for HIV infection risk for PWID/MWID on OAT relative to those off OAT.

$$Multiplier\ for\ shared\ injections\ on\ OAT = (1 - Rate\ ratio\ for\ risk\ HIV\ infection\ on\ OAT)$$

We used 95% confidence intervals from literature estimates to derive high and low ranges for the reduction in shared injections due to OAT.

4.3 Pre-exposure prophylaxis

To model the scale-up of pre-exposure prophylaxis (PrEP), following the approval of medication for use as PrEP in 2012, we included parameters for the number of HIV-negative MSM/MWID on PrEP, screening rates, duration that HIV-negative individuals remain on PrEP, as well as the efficacy of PrEP in reducing HIV transmission.

4.3.1 PrEP uptake

Model input parameters: We required total numbers of HIV-negative MSM/MWID receiving PrEP in 2012-2017 by city (4 parameter values), as well as the stratification by race/ethnicity (3 parameter values). We required parameters for screening rates for individuals on PrEP (1 parameter value), as well as the average duration that HIV-negative individuals remain identified while on PrEP (1 parameter value).

Identification and selection of evidence: We identified surveillance data and peer-reviewed literature as the best available evidence for estimating the number of individuals on PrEP (S2 Supplement Tables C4.3.1.1, C4.3.1.2, C4.3.2, C4.3.3, C4.3.4, C4.3.5) [128].

Derivation of model parameters

We set initial numbers of HIV-negative MSM/MWID on PrEP to zero in 2011 (baseline) to begin model calibration, as Truvada was not approved by the FDA until 2012 [129]. For all cities, we derived total numbers on PrEP for 2012 through 2017 (to account for its recent rapid growth in uptake among MSM) using AIDSVu surveillance data on the number of unique men who had at least one day of prescribed PrEP by ZIP code [130]. We used state-level growth from 2016 to 2017 to derive city-level estimates for the number of individuals on PrEP in 2017, as city-level data were not available. We derived ranges for the number of individuals on PrEP using a lower bound derived from Seattle &

King County surveillance reports [119] and an upper bound from literature estimates [131]. We derived race/ethnicity proportions receiving PrEP for New York City based on Medicaid prescription data [132], and for other cities except Miami using weighted national race/ethnic proportions of MSM accessing PrEP [128], weighted by statewide race/ethnic distribution of the MSM population [133]. For Miami, given a large disparity in race/ethnic composition between Florida State and Miami-Dade County, we weighted the PrEP proportion estimates with city-level MSM demographics that we estimated from model instantiation (S1 Supplement C, Section 3.1.1). To derive screening rates for individuals on PrEP, we assumed that individuals on PrEP were tested every three months as this was a requirement for PrEP [134] (S2 Supplement Table C4.3.4). To derive the average duration the HIV-negative individuals remain on PrEP, we assumed that individuals remained identified for 3-months to mirror CDC testing guidelines [134] (S2 Supplement Table C4.3.5).

Total HIV-negative individuals on PrEP

$$\begin{aligned} &Total\ susceptible\ MSM/MWID\ on\ PrEP\ (year) \\ &\qquad = Numbers\ reported\ directly\ in\ AIDSVu\ data \end{aligned}$$

Screening rates for individuals on PrEP

$$\begin{aligned} &Screening\ rates\ for\ MSM/MWID\ on\ PrEP \\ &\qquad = Assumption\ based\ on\ requirements\ for\ PrEP\ prescribing \end{aligned}$$

Average duration of time that individuals remain identified after screening (on PrEP)

$$\begin{aligned} &Average\ duration\ MSM/MWID\ remain\ identified\ on\ PrEP \\ &\qquad = Assumption\ based\ on\ requirements\ for\ PrEP\ prescribing \end{aligned}$$

4.3.2 PrEP effectiveness

Model input parameters: We required multipliers for the reduction in transmission due to PrEP for MSM/MWID, common across cities, race/ethnicity, and risk group (1 parameter value).

Identification and selection of evidence: We identified literature estimates for PrEP effectiveness in reducing HIV transmission as the best available evidence (S2 Supplement Table C4.3.3) [135,136].

Derivation of model parameters

We derived PrEP effectiveness from PrEP efficacy estimates for individuals maintaining optimal protective levels of adherence (≥4 doses/week) [135], multiplied by the proportion of individuals maintaining protective levels of adherence [136].

<u>PrEP effectiveness</u>

$$PrEP\ effectiveness = PrEP\ efficacy\ at\ optimal\ adherence * Proportion\ maintaining\ protective\ adherence$$

We derived ranges for PrEP efficacy from 95% confidence intervals in the source study.

5. Costs of Medical Care

We derived monthly costs among PLHIV from primary analysis of longitudinal HIV cohort health care utilization data, combined with unit costs for individual expenditure items. We estimated costs among HIV-negative individuals using nationally representative health care expenditure data from the general population.

5.1 Medical costs among PLHIV

Model input parameters: We required monthly health care costs for PLHIV, stratified by CD4 cell count (> 500 cells/μL; 200-499 cells/μL; < 200 cells/μL), treatment status (infected/unaware, diagnosed, on-ART, off-ART), and risk group (PWID, non-PWID), for each city (26 parameter values).

Identification and selection of evidence: We identified longitudinal cohort data from HIVRN data to estimate health resource use for PLHIV [92]. For unit costs of individual health care components, we selected Medicaid physician fee schedules [137], prescription drug costs from national FSS price schedules [138], costs of diagnostic testing [139], and cost estimates for emergency department visits and inpatient hospitalizations [140] (S2 Supplement Tables C5.1.1, C5.1.2, C5.1.3, C5.1.4, C5.1.5, C5.1.6).

Derivation of model parameters

We applied unit costs to HIVRN utilization records to derive total medical care costs among PLHIV, stratified by risk group and CD4 cell count. We estimated medical care costs of PLHIV by HIVRN region and applied estimates to cities within each region. Full details of cost estimation are described elsewhere [141].

$$Mean\ health\ care\ costs\ among\ PLHIV_{Risk \times CD4 \times ART} = Directly\ estimated\ from\ HIVRN\ data$$

We used 95% confidence intervals derived from HIVRN primary analysis estimates to derive upper and lower range values.

5.2 Medical costs among HIV-negative

Model input parameters: We required monthly health care costs for HIV-negative, stratified by risk group (PWID, non-PWID), for each city (2 parameter values).

Identification and selection of evidence: We identified population-level estimates of health care costs from MEPS data in 2016, stratified by census region, to capture health care costs of HIV-negative individuals in our model [142] (S2 Supplement Table C5.2.1). We used a multiplier for costs among HIV-negative PWID based on observed cost

differences among HIV-positive PWID and non-PWID in HIVRN primary analysis (S2 Supplement Table C5.2.2) [143,144].

Derivation of model parameters

$$\begin{aligned} &Mean\ yearly\ health\ care\ costs\ among\ susceptible \\ &\qquad = Directly\ estimated\ from\ MEPS\ data \end{aligned}$$

$$Monthly\ health\ care\ costs\ among\ susceptible = \frac{Mean\ yearly\ expenditure\ per\ person}{12}$$

$$\begin{aligned} &Monthly\ health\ care\ costs\ among\ susceptible\ PWID \\ &\qquad = Monthly\ health\ care\ costs\ among\ susceptible * PWID\ cost\ multiplier \end{aligned}$$

We used 95% confidence intervals from MEPS estimates to derive high and low ranges. We derived ranges for PWID costs among HIV-negative from literature estimates.

6. Health utility weights

Quality-adjusted life years (QALYs) for individuals were a primary outcome of our cost-effectiveness model. To calculate QALYs, we included model input parameters that assigned health utility weights to each health state, based on literature estimates and additional assumptions.

6.1 PLHIV

Model input parameters: We required health utility weights for PLHIV, stratified by risk group (PWID vs. non-PWID), OAT status among PWID (OAT vs. non-OAT), and CD4 cell count, stratified by infected, diagnosed, and on-ART (27 parameter values).

Identification and selection of evidence: We identified longitudinal cohort studies as the best available evidence to derive health utility weights. We selected health utility weights from literature sources for infected PLHIV [145,146,147,148,149,150] (S2 Supplement Table C6.2.1), diagnosed PLHIV [145,146,147,148,149,150] (S2 Supplement Table C6.2.2), on-ART PLHIV [145,146,147,148,149,150] (S2 Supplement Table C6.2.3), all PWID (S2 Supplement Table C6.2.4) [20], and OAT among PWID (S2 Supplement Table C6.2.5) [151].

__Derivation of model parameters__

$$Health\,utility\,weight\,PLHIV\,(infected/diagnosed/on\,ART)_{CD4} = Synthesized\,from\,literature\,estimates$$

$$Health\,utility\,weight\,PLHIV\,PWID\,(infected/diagnosed/on\,ART)_{CD4} = Health\,utility\,weight\,PLHIV\,(infected/diagnosed/on\,ART)_{CD4} * PWID\,multiplier$$

$$Health\,utility\,weight\,PLHIV\,PWID\,on\,OAT\,(infected/diagnosed/on\,ART)_{CD4} = Health\,utility\,weight\,(infected/diagnosed/on\,ART)_{CD4} * OAT\,multiplier$$

We used 95% confidence intervals from literature estimates to derive ranges for health utility weights.

6.2 HIV-uninfected

Model input parameters: We required health utility weights for HIV-negative individuals, stratified by risk group (PWID vs. non-PWID), OAT status among PWID (OAT vs. non-OAT) (3 parameter values).

Identification and selection of evidence: We identified longitudinal cohort studies as the best available evidence to derive health utility weights. For PWID health utility weights, we used literature estimates (S2 Supplement Table 6.2.4) [20], for OAT vs. non-OAT we used literature estimates (S2 Supplement Table 6.2.5) [151].

Derivation of model parameters

We assumed quality of life weights for non-PWID, HIV-negative individuals to be 1, as a reference health state for relative quality of life weights in other health states.

$$Health\,utility\,weight\,HIV-negative = 1\;(reference\,health\,state)$$

$$Health\,utility\,weight\,HIV-negative\,PWID = Health\,utility\,weight\,HIV-negative * PWID\,multiplier$$

$$Health\,utility\,weight\,HIV-negative\,PWID\,on\,OAT = Health\,utility\,weight\,HIV-negative\,PWID * OAT\,multiplier$$

We used 95% confidence intervals from literature estimates to derive high and low ranges for health utility weights.

S1 Supplement D: Primary Analyses

This supplement provides a brief description of the data sources from which we conducted primary data analysis to populate our model parameter values.

1. National HIV Behavioral Surveillance (NHBS)

Data source description

The Centre for Disease Control and Prevention (CDC) conducts behavioral surveillance in rotating annual cycles and focuses on a different population at increased risk for HIV in each year (cycle): men who have sex with men (MSM), persons who inject drugs (PWID), and heterosexuals at increased risk for HIV infection (HET). NHBS is conducted in 20 metropolitan statistical areas (MSAs). The MSM cycle recruited males (≥ 18 years) reporting any oral or anal sex with a male partner during their lifetime using Venue-based, time-space sampling (VBS). The PWID cycle recruited adults (≥ 18 years) reporting any non-prescribed injection drug use in the last 12 months (L12M) using respondent-driven sampling (RDS). The HET cycle recruited adults (18-60 years) reporting vaginal or anal sex with an opposite sex partner in L12M and residing in poverty areas (census tracts where ≥ 20% of residents live below the poverty threshold) using the RDS method.

Study sample

We used data from cycle 3 and cycle 4 on MSM (2011, 2014), PWID (2012, 2015), and HETs (2013, 2016) in the 5 project areas of interest (Atlanta, Los Angeles, Miami, New York City, and Seattle). Data were available for all five cities except for the HET cycle 4, which was not conducted in New York City, and Seattle. All participants with complete interview data who were sexually active, or who actively injected drugs in the L12M were included in our analysis. To fit the specifications of our simulation model, we excluded participants who were older than 64 years. Additionally, we excluded those who self-reported HIV positive, as we aimed to estimate risk behaviors and PrEP uptake among HIV negative or HIV status-unaware populations.

Statistical analysis

Descriptive analysis was conducted to obtain summary statistics on parameters of interest stratified by city, race/ethnicity, gender, risk group (MSM, MSM/PWID, PWID, HET) and calendar year. As MSM/PWID participating in the MSM cycle might be different from those identified in the PWID cycle due to different sampling methods, we estimated two sets of parameters for MSM/PWID using data from each cycle separately. For parameters such as annual number of opposite sex partners and same sex partners, we obtained the mean, standard deviation, median, interquartile range, and the 10th and 90th percentiles of the estimates. Due to the great uncertainty in quantifying individuals' sexual risk behaviors, these multiple statistics describing the risk behavior distribution will assist in our calibration of these parameters. We calculated the proportion and the exact 95% confidence intervals (CIs) of individuals who use condom every time with opposite/same partners, PWID who

use needles which have been used by others, MSM who report PrEP indication/uptake, and individuals who receive HIV testing, during L12M. For the calculation of proportion of individuals who receive HIV testing in L12M, the denominator contained HIV negative or HIV status-unaware participants and HIV positive participants who were diagnosed in L12M.

Demographics of sex partners were collected in PWID cycle 4 and HET cycle 4 only, and were used to estimate the assortative mixing by race/ethnicity for PWID and HET, respectively stratified by city. First, we cross tabulated the participants' race/ethnicity by their most recent sex partners' race/ethnicity. Second, we calculated the *Newman's* assortative mixing coefficients and 95% CIs stratified by race/ethnicity [152]. This coefficient captures the probability of mixing with the same race/ethnicity, accounting for mixing with the same race/ethnicity by chance alone. As sensitivity analysis, we also calculated the assortative mixing coefficients using data from participants' up to three most recent sex partners.

2. Medical Monitoring Project (MMP)

Data source description

The National HIV Surveillance System (NHSS) collects a core set of data on the characteristics of persons diagnosed with HIV infection in all US states and dependent areas. HIV surveillance projects such as the Centers for Disease Control and Prevention's (CDC) Medical Monitoring Project (MMP) provide complementary information about clinical outcomes of HIV infection, care seeking and care utilization of persons living with HIV, and ongoing transmission risk behaviors. From 2005 to 2014, MMP's study design relied on a probability sample of HIV diagnosed persons sampled from HIV facilities to generate nationally representative estimates of clinical outcomes and HIV-related behaviors. U.S. states and one territory were sampled, then facilities in those areas providing outpatient HIV care, and finally, eligible PLHIV. MMP methods, including non-response bias analysis and weighting techniques, have been described in detail elsewhere [153,154].

Study sample

The national population of inference for MMP is all HIV-diagnosed persons aged ≥ 18 years living in the United States. For each project area, the population of inference is all HIV diagnosed persons aged ≥ 18 years whose most recently reported address was within the project area. We to calculate city-specific estimates, we received data from 5 project areas, 1) Georgia (Atlanta), 2) Florida (Miami), 3) Los Angeles county, 4) New York City, 5) Washington state (Seattle).

Statistical analysis

We received descriptive analysis from MMP of summary statistics for parameters of interest stratified by city or state, race/ethnicity, gender, risk group (MSM, MSM/PWID, PWID, HET) from 2010 and 2014. The MMP analyzed matched interview and medical

record abstraction data from the 2010 and 2014 data collection cycles. Interview and medical record abstraction data were collected in each data collection cycle for the 2009-2014 data cycles. Data were reported for the 2010 and 2014 data cycles, collected from June 2010–May 2011 and June 2014-May 2015. The data were weighted to adjust for sampling probabilities.

Parameters included CD4 distribution among on-ART and off-ART PLHIV, distribution of CD4 cell counts at diagnosis, as well as the proportion of PLHIV initiating ART within 30 days of diagnosis, stratified by CD4 cell count. We received proportions of heterosexual and homosexual assortative mixing for the last sexual partner and last 5 sexual partners, as well as number of same and opposite sexual partners in the past 12 months and proportion of individuals using a condom with all sexual partners.

3. HIV Research Network (HIVRN)

Data source description

The HIVRN care providers are a consortium of adult and pediatric clinics located in the Northeast (Rochester, Boston, New York City, Baltimore, and Philadelphia), South (Dallas, Memphis, Tampa) and West (Portland, Oakland, and San Diego) regions of the United States. HIVRN sites abstract specified data elements from patients' medical records, including demographic data, service utilization, medications, and laboratory tests; abstracted data are assembled into a single database after quality assurance review.

Study sample

We included individuals aged 15-64 who were enrolled in participating clinics between 01 January 2007 and 30 September 2015.

Statistical analysis

Disease progression on ART characterized by the transition probability between CD4 strata, ART dropout probability, and ART re-initiation probability were simultaneously estimated using a continuous-time multi-state Markov model. This was operationalized by a matrix with 14 possible instantaneous transitions (CD4 ≥500 to 200-499; ≥500 to off-ART; ≥500 to death; 200-499 to ≥500; 200-499 to <200; 200-499 to off-ART; 200-499 to death; <200 to 200-499; <200 to off-ART; <200 to death; off-ART to ≥500; off-ART to 200-499; off-ART to <200; and off-ART to death). Analysis was stratified by region, and adjusted for risk group, race/ethnicity and gender.

4. National Survey of Family Growth (NSFG)

Data source description

The NSFG is a nationally representative survey of the U.S. household population using stratified multi-stage area probability sampling. Face-to-face interviews and audio computer-assisted self-interviews were conducted with women and men aged 15-44 years, capturing information such as sexual behavior, family life, and health status. The public use data files were available on the NSFG website. Additionally, a user agreement can be signed to gain access to a REGION file, which includes a 4-category REGION variable (Northeast, West, Midwest, and South), capturing respondent's residence at the time of the interview. More detailed residence information can only be accessed at the Census Research Data Centers.

Study sample

We obtained data from the 2011-2013 NSFG survey, including the public use data file and the REGION variable. A total of 5,601 women and 4,815 men were included. We used the NSFG data to estimate the sexual risk behaviors of HETs and low-risk MSM. MSM was defined as male who report having a male sex partner in the L12M. HET was defined as all participants except MSM and those who report injecting drugs in the L12M. We did not obtain the estimates for PWID due to the small sample size (N=47).

Statistical analysis

We performed survey analysis accounting for the stratified probability sampling design and sampling weight to obtain summary statistics on parameters of interest. The SAS *proc surveymeans* procedure was used to obtain the weighted means and 95% CIs for parameters including annual number of same/opposite sex partners, and *proc surveyfreq* was used to obtain the proportion and 95% CIs of individuals who use condom every time in the L12M. The *domain statement* was used to obtain estimates on subgroups stratified by region, race/ethnicity, gender, and risk group (MSM, HET).

Only the demographics of opposite sex partners were available, and were used to estimate the assortative mixing by race/ethnicity for HETs stratified by region. We calculated the *Newman's* assortative mixing coefficients and 95% CIs for each race/ethnicity group [152].

5. HIV Rapid Testing & Counseling in Sexually Transmitted Disease Clinics (the AWARE study)

Data source description

The AWARE study recruited 5,012 HIV negative or HIV status-unaware patients (≥ 18 years) seeking services from sexually transmitted disease clinics in nine U.S. cities (Pittsburgh, Jacksonville, Los Angeles, Miami, Portland, Seattle, Columbia, San Francisco, and Washington, DC.) between April and December 2010. It was a randomized controlled trial designed to assess the effect of brief patient-centered risk-reduction counseling at the time of a rapid HIV test on the subsequent acquisition of sexually transmitted infections. At

the baseline and 6-month follow-up, participants were assessed for their sexual risk behaviors and injection drug use in the last 6 months (L6M).

Study sample

We included all participants aged between 18-64 years in our analysis.

Statistical analysis

Descriptive analysis was performed to obtain estimates on participants' baseline HIV risk behaviors in the L6M among the full study sample, as well as among samples from each of the three cities of interest (Los Angeles, Miami, and Seattle) separately. All analysis was stratified by risk group (MSM, MSM/PWID, PWID, and HET), race/ethnicity and gender. We calculated the median and interquartile range estimates for the number of same and opposite sex partners, respectively. For MSM and MSM/PWID, their number of same sex partners was approximated by the number of anal sex partners, and the number of opposite sex partners was approximated by the number of vaginal sex partners. Additionally, we calculated the proportions and the exact 95% CIs of individuals who use condom every time, and PWID who report using needles which have been used by others.

6. The AIDS Linked to the IntraVenous Experience (ALIVE)

Data source description

The ALIVE study is a prospective cohort of adult (18 years +) who reported injection drug use within the past 11 years, recruited through community outreach in Baltimore, MD. The initial enrollment began in 1988 with 2,938 participants recruited (88% of participants were black/African American), and an additional 1,733 PWID were enrolled through later recruitment efforts. Follow-up visits for ALIVE occur semi-annually. At each visit, participants complete surveys and laboratory testing. Information on behavior, life events, and health-related outcomes is captured using audio-computer assisted self-interview.

Study sample

We included all black/African American participants who had an assessment (regardless of whether it is a baseline or follow-up) completed in 2010, and reported injection drug use in the L6M. We excluded those who were HIV positive, or those who were older than 64 years at the time of assessment.

Statistical analysis

Descriptive analysis was employed to obtain estimates on risk behaviors using data from the first assessment in 2010 of each participant. All analysis was stratified by risk group (MSM/PWID, and PWID), and gender. We calculated the median and interquartile range estimates for the number of same and opposite sex partners, respectively, and calculated the proportions and the exact 95% CIs of individuals who use condom every time, and PWID who report sharing needles.

7. Behavioral Risk Factor Surveillance System (BRFSS)

Data source description

The CDC's BRFSS is the largest health-related cross-sectional surveillance survey of U.S. residents and collects health-related risk behaviors and chronic health conditions from adults, aged 18 years and older. The BRFSS uses two samples: one for landline telephone respondents and one for cellular telephone respondents, and includes state-level stratification. The public use data files were available on the BRFSS website (www.cdc.gov/brfss) with respondents' geographic information available at the state level, and included indication of residence in a Metropolitan Statistical Area.

Study sample

We obtained data from the public use file of the 2010 BRFSS survey, including the MSCODE variable indicating non-nominal MSA residence. We included all adults aged <65 residing in the center city of an MSA or inside the county containing the center city of an MSA (MSCODE==1 | MSCODE==2) for each respective state, and assumed that population-level HIV testing behavior reported in BRFSS was representative of HIV testing behavior for low risk HET and for low risk MSM.

Statistical analysis

We estimated gender and race/ethnicity stratified HIV testing in the L12M accounting for both design weighting and iterative proportional fitting, and we used weights assigned to each respondent for the landline telephone and cellular telephone combined data to obtain the weighted means and 95% CIs.

8. New York City Community Health Survey (CHS)

Data source description

The New York City CHS is an annual cross-sectional telephone survey of randomly selected adults aged 18 and older from all five boroughs of New York City (Manhattan, Brooklyn, Queens, Bronx, and Staten Island). The survey is conducted using a computer-assisted telephone interviewing system and collects self-reported data from selected respondents with landline telephones and cell phones. The public use data files were available on the CHS website (https://www1.nyc.gov/site/doh/data/data-sets/community-health-survey-public-use-data.page)

Study sample

We obtained data from the public use file of the 2010 CHS survey, and we included all women aged <65 and all men aged <65 not reporting having sex with a man in the past 12 months (MSM==2). We assumed that HIV testing behavior reported in CHS was representative of HIV testing behavior for low risk HET and low risk MSM.

Statistical analysis

We estimated gender and race/ethnicity stratified HIV testing in the L12M accounting for probability of selection and post-stratification weights to obtain the weighted means and 95% CIs.

9. Treatment Episode Data Set (TEDS)

Data source description

The Substance Abuse and Mental Health Services Administration's (SAMHSA) TEDS admission data set is the only national client-level database on substance abuse treatment, and includes routinely collected information on all individuals admitted to facilities that receive public funds. In TEDS, an admission is defined as the formal acceptance of an individual into substance abuse treatment. The public use data files were available on the TEDS website (https://wwwdasis.samhsa.gov/dasis2/teds.htm) with geographic information available for admissions at the state level, including Core-Based Statistical Areas (CBSA) indicators.

Study sample

We obtained data from the 2010 to 2014 TEDS public use files, and we included all individuals aged ≥15 residing in the CBSA corresponding to each respective city, and defined PWID with OUD as individuals reporting: (i) the primary, secondary or tertiary substance use of heroin, non-prescription methadone, and other opiates and synthetics (including buprenorphine, codeine, Hydrocodone, hydromorphone, meperidine, morphine, opium, oxycodone, pentazocine, propoxyphene, tramadol, and any other drug with morphine-like effects); and (ii) injection as route of administration. As the data did not capture repeated treatment admissions, we assumed that each admission reported in TEDS was for a unique individual.

Statistical analysis

We estimated the number of individuals in a given year receiving opioid agonist treatment (i.e. medication-assisted treatment) by gender and race/ethnicity for every city except Atlanta (all OAT data was missing for Georgia (variable METHUSE)). As TEDS has been shown to be a conservative source of treatment coverage [155], we defined a conservative lower range by excluding PWID with OUD aged>55 and defined a liberal upper range by including ALL individuals reporting use of heroin.

S1 Supplement E: Model Calibration/Validation, Parameter Ranges, and PSA Probability Distributions

Since data used to populate the model were not always available, representative for specific subpopulations of interest, up-to-date, and/or exhibiting large variation, we undertook an extensive model calibration and validation exercise which will be described in further detail in an upcoming manuscript [156]. We provide an overview below.

We adopted a direct-search, Nelder-Mead algorithm, to iteratively calibrate key parameters with high uncertainty against three sets of observed calibration endpoints, including the total number of diagnosed PLHIV in each year, annual new HIV diagnoses, and annual all-cause mortality among PLHIV. We adjusted parameter values within pre-specified ranges using 2011 as the baseline year, and compared model outputs with reported values between 2012-2015 until the weighted mean percentage deviation (goodness-of-fit (GOF) metric) was minimized. We selected the set of key parameters for calibration using a one-at-a-time factor screening approach (the Morris method), on the basis of underlying uncertainty associated with evidence input estimates and their effects on the calibration targets. We also conducted model validation to assess face-validity, as well as the internal and external validity of the model. In particular, we externally validated model projections against the empirically estimated annual number of HIV incident cases.

In subsequent sections, we discuss the process for deriving model calibration targets for each city. Due to limitations in publically available surveillance data, and varying data quality across cities, we also documented the process by which we triangulated city-specific calibration targets from alternative sources. Data and evidence sources for model calibration and validation targets are attached in supplementary Excel files (S2 Supplement Tables E2 – E7).

1. Model Calibration Targets

We derived model calibration targets directly from city-level HIV surveillance reports when available, and describe the methods and assumptions required to create the calibration targets for our model. As with other evidence sources, any race/ethnicity other than black/African American or Hispanic/Latino, was categorized as white/other, and any risk group other than MSM, PWID and MWID was classified as HET to match overall totals of diagnoses, total PLHIV and all-cause deaths. Other assumptions that were required due to data limitations for specific cities are detailed below.

1.1 New HIV Diagnoses

Yearly numbers of new HIV diagnoses were derived directly from city-level HIV surveillance where available. In New York City [157], Los Angeles [158], Baltimore [159], and Seattle [160], numbers of new HIV diagnoses were available for all years with

minimal triangulation required. In most cases, two-way stratified totals were reported (by race/ethnicity and gender, race/ethnicity and risk group, and risk group and gender), and we triangulated three-way stratified estimates to match our target model calibration inputs. For Miami, the number of new HIV diagnoses were available for 2013-2015 [161], stratified by race/ethnicity and gender/risk group. We assumed that race/ethnicity proportions were equivalent for each risk group. We derived new diagnoses for 2012, using the state level diagnoses, adjusted for the relative proportion of new diagnoses from Miami for each PLHIV strata. For the Atlanta EMA (city boundary encompassed the counties described in S1 Supplement A), we used a 2014 EMA report of new HIV diagnoses [162], combined with Georgia state level surveillance estimates to derive relative percentages between the city and state for each strata of PLHIV [162,163]. We used these percentages to derive city-level estimates for 2012, 2013 and 2015, based on the assumption that city-level numbers remained the same relative proportion of state-level numbers for each strata as in 2014.

1.2 Total Diagnosed PLHIV

Total population numbers for diagnosed PLHIV were derived directly from city-level HIV surveillance reports and data queries where available. In New York City [157], Los Angeles [158], Seattle [160], and Baltimore [159], total numbers of PLHIV were available for all years. As with new diagnoses, the majority were two-way or fully stratified and required minimal triangulation. For Miami, we used data on total diagnosed PLHIV for Miami-Dade county from AIDSVu for 2011-2014 [164], and Miami-Dade county HIV surveillance data for 2015 [68]. For Atlanta, we used 2014 EMA reports to triangulate PLHIV numbers for 2011-2013 and 2015, combined with state-level estimates to derive relative percentages between city and state for each strata of PLHIV [162]. As with new diagnoses, we used these percentages for derive city-level estimates of the diagnosed PLHIV population for 2012, 2013, and 2015, using state-level numbers, assuming that city-level numbers remained the same relative proportion of state-level numbers for each strata in those years as 2014.

1.3 All-cause Mortality among PLHIV

We derived yearly all-cause mortality among PLHIV from city-level HIV surveillance reports where possible. For New York City [157], and Los Angeles [158], two-way stratified totals were reported for all-cause mortality among PLHIV, and we triangulated using the same methods as for new HIV diagnoses. For Baltimore, only total HIV-specific deaths were reported at the city-level [159], so we used state-level differences between the number of HIV-specific deaths and all-cause deaths among PLHIV to derive relative ratios between HIV-specific mortality and all-cause mortality. We derived the numbers of all-cause deaths for PLHIV in Baltimore using these proportions to distribute all-cause deaths to each stratified subgroup. In Miami, we used state-level numbers of HIV-

specific deaths, combined with state-level all-cause mortality among PLHIV to derive a multiplier for HIV-specific deaths [165]. We applied this multiplier to reported numbers of city-level HIV-specific deaths to derive the number of all-cause deaths among PLHIV for Miami. In Seattle, fully stratified city-level numbers for all-cause mortality among PLHIV were not reported, so we used state-level estimates to derive relative proportions of deaths for every strata [166]. We applied state-level proportions to city-level totals for all-cause mortality among PLHIV to derive fully stratified city-level mortality totals [160]. For Atlanta, we used state-level estimates of all-cause mortality from 2012-2015, stratified individually by risk group, gender and race/ethnicity [167], weighted by the relative proportion of diagnosed PLHIV within each strata in the 20 counties, relative to the entire state.

2. Model Validation Targets

We used yearly incidence estimates for each city as a validation step for our model. Among the six cities, New York City reported separate incidence estimates for each year from 2012-2015. For other cities, including Los Angeles (for which independent city-level incidence estimates were only available for 2012 and 2013), we derived incidence estimates using the difference between observed yearly state-level diagnoses and yearly state-level incidence estimated by the CDC [167]. We used these proportions to derive city-level incidence numbers based on the number of city-level diagnoses.

3. Population Growth Projections

To ensure that our model matched long-term population growth projections and changing demographics in each city, we used external reports and/or data to adjust total population growth parameters. Projections stratified by age, gender and race/ethnicity, were available for Atlanta [79], Baltimore [80], Los Angeles [81], and Miami [82]. For New York City, race/ethnicity stratified growth projections were not available at the city, county or state level, so we triangulated projections using age-gender stratified estimates combined with national trends for changing race/ethnic population compositions to 2040 [83,84]. For Seattle, stratified growth projections were not available at the city level, so we used population projections for Washington State [85]. We incorporated these projections to ensure that population growth parameters produced projections that matched the overall growth rates from long-term projections accounting for external factors and trends affecting city growth rates.

4. Probabilistic Sensitivity Analysis

Prior parameter ranges and probability distributions were used in both model calibration and probabilistic sensitivity analysis (PSA) processes from which parameter values were randomly drawn. Using distributional assumption guidelines [168,169], we fit each

parameter with parametric distributions according to the data type and quality of available evidence (S1 Supplement Table E1). Given these differences, the fitted distributions do not necessarily map to the parameter categories presented earlier.

S1 Supplement Figure E1. Data Quality Assessment for Calibration/Validation Targets

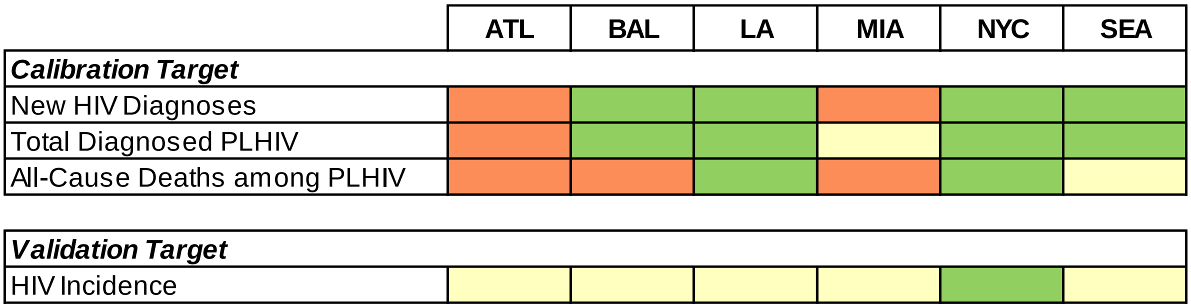

High Quality - Surveillance data reported without limitations on geographic representativeness, population stratification or time period

Moderate Quality - Surveillance data reported with some limitations on geographic representativeness, population stratification or time period

Low Quality - Surveillance data reported with significant limitations on geographic representativeness, population stratification or time period

S1 Supplement Table E1: PSA Distribution Selection by Parameter Type

Scenario or data type	Data Requirement	Distribution	Example
Census data†	N/A	N/A	Initial population stratification; population dynamics
Surveillance report data†	N/A	N/A	New diagnoses, diagnosed population
Proportion data - Binomial (1)	Proportion, Total number	Beta	MSM population
Proportion data - Binomial (2)	Point estimate, CI	Beta	Sexual assortativity
Proportion data - Binomial (3)	Point estimate, Min, Max	Beta Pert	PWID population
Proportion data - Multinomial (1)	Number of each category	Dirichlet	Initial CD4 distribution among PLHIV on ART
Proportion data - Multinomial (2)	Point estimate, Min, Max	Beta Pert + Standardization	Initial CD4 distribution among undiagnosed PLHIV
Probability data	Point estimate, CI	Beta	Transmission probability
Efficacy data (RR/OR/HR)	Point estimate, CI	Lognormal	OAT efficacy
Multiplier/Ratio	Point estimate, CI	Lognormal	Multiplier of utility for PWID on OAT
Rate	Rate (number of events), Time-period	Poisson	ART initiation rate
Health utility data	Point estimate, CI	Beta	Health utility weights
Cost data	Mean, Standard error	Gamma	Health care costs for HIV uninfected
Non-probability behavioural data	Point estimate, CI	Gamma	Number of sexal partners
Other data with only point estimates and ranges	Point estimate, Min, Max	Beta Pert	Sexual transmissibility
Inconsistent data from multiple sources	Min, Max	Uniform	MSM/PWID population, PrEP volume
Data based on assumption	N/A	Uniform + wide range	Health care costs for HIV-uninfected PWID
Jointly distributed data	Point estimate, Variance-covariance matrix OR Credible-interval contour	Bivariate Normal	Duration of acute stage and transmission probability during acute stage

† We did not explicitly model distributional assumptions and ranges for census and surveillance report data given the relatively low levels of uncertainty for these parameters.

S1 Supplement F: Scientific Advisory Committee Survey

Our review of literature and publicly available reports was able to provide the majority of data values needed for the dynamic compartmental model. However, where data was unavailable or was not rated highly in our quality assessment, we sought the expert advice from members of our Scientific Advisory Committee (SAC) on how to proceed in finding additional sources, and utilizing the data available to us.

To track experts' advice, we developed a comprehensive, web-based survey tailored for each of the cities using SurveyMonkey® and sent an invitation to our SAC leads. The survey asked 60 questions and was designed to take less than an hour to complete, with mandatory questions built throughout to elicit complete responsiveness. Participants were asked to review the best data points we found, identify additional sources we were not aware of, and describe their preference for assumptions we offered to improve the representativeness of data we worked with using triangulation. Participants were also encouraged to share the survey with public health experts in their respective city for additional review and input. The full survey is attached in a supplementary PDF document.

The survey was first made available on July 20, 2017 and remained open until October 31, 2017. 1 to 7 experts completed the survey per city, including 9 SAC members and 5 additional local public health experts. Two participants completed the survey for more than one city. The survey also revealed additional data sources identified by respondents, including internal data/reports not publicly available. We were able to access these reports directly or through assistance from our contacts within each city.

Using respondents' advice, we applied the following assumptions to adjust or incorporate the best available data into our model's starting values (S1 Supplement Table F1).

S1 Supplement Table F1: SAC Survey Results

Confidence in assumption		
N/A	Least	Most

Parameter	Assumption	ATL	BAL	LA	MIA	NY	SEA
Population size of PWID	Representative of city-level gender distribution of general population						
	Representative of national-level gender distribution						
Population size of MWID	Representative of national race/ethnicity specific estimates for proportion of male PWID who have sex with men						
	Representative of city-level estimates for males who are MSM						
	Representative of city-level estimates for males who are PWID						
Population size of HIV+ MSM	Equal proportion of MSM who are HIV positive across race/ethnicities						
	Representative of city-level cumulative diagnoses of HIV among MSM						
	Representative of city-level recent (last year) diagnoses of HIV among MSM						
Population size of HIV+ MWID	Representative of city-level cumulative diagnoses of HIV						
	Representative of city-level recent (last year) diagnoses of HIV						
Population size of HIV+ PWID	Equal proportion of PWID who are HIV positive across race/ethnicities						
	Representative of city-level cumulative diagnoses of HIV among PWID						
	Representative of city-level recent (last year) diagnoses of HIV among PWID						
Cross-tabulation of diagnosed PLHIV by race/ethnicity and transmission group	Proportional to city-level distribution of overall diagnosed PLHIV by race/ethnicity						
	Weighting the distribution of race/ethnicity within risk groups using published evidence at the state or other city level						
Distribution of "other" transmission category to four transmission groups	Redistribution using cumulative diagnoses proportions for risk group						
	Redistribution using past year diagnoses proportions for risk group						
	Redistribution proportional to general population						
Estimates for MSM sexual risk behaviour	Unweighted estimates based on NHBS-MSM						
	Recruitment weighted (venue-based sampling weights) estimates based on NHBS-MSM						
	Assumption-weighted estimate						
Estimates for PWID sexual risk behaviour	Unweighted estimates based on the NHBS-IDU						
	Recruitment-weighted (respondent-driven sampling weights) estimates based on the NHBS-IDU						
	For Baltimore only: Estimates based on race/ethnicity specific estimates from the ALIVE cohort						
Estimates for HET sexual risk behaviour	How confident are you that the region-specific estimates based on the NSFG represent true estimates of the sexual risk behaviors among all HETs						
Estimates for MWID HIV risk behaviour	Assume the sexual risk behaviors of MSM-PWID to be the same across six cities						
	Assume the sexual risk behaviors of MSM-PWID to be the same as MSM						
Estimates for MWID needle sharing behaviour	Assume the needle sharing probability of MSM-PWID to be the same across six cities						
	Assume the needle sharing probability of MSM-PWID to be the same as PWID						
ART effectiveness on HIV transmission by needle sharing	How confident are you about the estimates of ART effectiveness on HIV transmission by needle sharing						
Estimates on annual probability of ART interruption	How confident are you that the true probability of ART interruption can be determined by region-specific estimates						
HIV testing	Assume testing events are proportional to past year diagnoses						
	Assume testing events are proportional to cumulative diagnoses						
	Assume testing events are proportional to susceptible population size						
	Assume testing events are proportional to a susceptible-population-weighted probability of being tested derived from NHBS or BFRSS of reporting a HIV test receipt in the last 12 months						
Syringe distribution	Identical to national gender and race/ethnicity weighted syringe distribution numbers obtained from the Dave Purchase Memorial National Survey of Syringe Exchange Programs and reported by NASEN						
	Identical city-level average coverage for each risk, gender and race/ethnicity strata						
Opioid agonist treatment coverage	Assume distribution is representative of city-level gender distribution of the general population						
	Assume distribution is representative of national-level gender distribution of OAT clients						
Buprenorphine utilization	The number of PWID receiving BUP is determined to be 25% of state-level reported maximum physician prescribing capacity, based on evidence of the proportion of heroin users in a national survey of BUP prescription recipients						
	The number of PWID receiving BUP is determined to be 10% of state-level DATA-reported maximum physician prescribing capacity, based on evidence of the proportion of DATA-waivered physicians in NYC that accept Medicaid						
	The number of PWID receiving BUP is determined to be equivalent to the number of quarterly prescriptions from state Medicaid data of drugs containing BUP in the non-proprietary drug name						

PWID - People who inject drugs; MSM - Men who have sex with men; MWID - MSM who inject drugs; HET - Heterosexual ; PLHIV - People living with HIV; OAT - Opioid agonist treatment; BUP - Buprenorphine

Evidence Source Tables

Evidence tables are included in an accompanying Excel file.

References

1. Cooper N, Sutton A, Ades A, Paisley S, Jones D, et al. (2007) Use of evidence in economic decision models: practical issues and methodological challenges. Health Econ 16: 1277-1286.
2. Paisley S (2016) Identification of Evidence for Key Parameters in Decision-Analytic Models of Cost Effectiveness: A Description of Sources and a Recommended Minimum Search Requirement. PharmacoEconomics 34: 597-608.
3. Zechmeister-Koss I, Schnell-Inderst P, Zauner G (2014) Appropriate evidence sources for populating decision analytic models within health technology assessment (HTA): a systematic review of HTA manuals and health economic guidelines. Med Decis Making 34: 288-299.
4. Nosyk B, Min J, Lima V, Hogg R, Montaner J, et al. (2015) Cost-effectiveness of population-level expansion of highly active antiretroviral treatment for HIV in British Columbia, Canada: a modelling study. Lancet HIV 2: e393-e400.
5. Nosyk B, Min JE, Krebs E, Zang X, Compton M, et al. (2017) The Cost-Effectiveness of Human Immunodeficiency Virus Testing and Treatment Engagement Initiatives in British Columbia, Canada: 2011–2013. Clinical Infectious Diseases 66: 765-777.
6. Nosyk B, Zang X, Min JE, Krebs E, Lima VD, et al. (2017) Relative effects of antiretroviral therapy and harm reduction initiatives on HIV incidence in British Columbia, Canada, 1996-2013: a modelling study. The Lancet HIV 4: E303-E310.
7. Zang X, Krebs E, Wang L, Marshall B, Granich R, et al. (2018) Structural design and data requirements for simulation modeling in HIV/AIDS: a narrative review. Submitted.
8. Cooper N, Coyle D, Abrams K, Mugford M, Sutton A (2005) Use of evidence in decision models: an appraisal of health technology assessments in the UK since 1997. J Health Serv Res Policy 10: 245-250.
9. Centre for Evidence-Based Medicine (2009) Oxford Centre for Evidence-based Medicine – Levels of Evidence.
10. Weller S, Davis K (2002) Condom effectiveness in reducing heterosexual HIV transmission. The Cochrane database of systematic reviews: CD003255.
11. Giannou FK, Tsiara CG, Nikolopoulos GK, Talias M, Benetou V, et al. (2016) Condom effectiveness in reducing heterosexual HIV transmission: a systematic review and meta-analysis of studies on HIV serodiscordant couples. Expert Review of Pharmacoeconomics & Outcomes Research 16: 489-499.
12. Smith DK, Herbst JH, Zhang XJ, Rose CE (2015) Condom Effectiveness for HIV Prevention by Consistency of Use Among Men Who Have Sex With Men in the United States. Jaids-Journal of Acquired Immune Deficiency Syndromes 68: 337-344.
13. World Health Organization (2011) Prevention and treatment of HIV and other sexually transmitted infections among men who have sex with men and transgender people: recommendations for a public health approach, 2011. . Geneva: World Health Organization.
14. Cohen MS, Chen YQ, McCauley M, Gamble T, Hosseinipour MC, et al. (2011) Prevention of HIV-1 Infection with Early Antiretroviral Therapy. New England Journal of Medicine 365: 493-505.
15. Baggaley RF, White RG, Hollingsworth TD, Boily MC (2013) Heterosexual HIV-1 Infectiousness and Antiretroviral Use Systematic Review of Prospective Studies of Discordant Couples. Epidemiology 24: 110-121.

16. Rodger AJ, Cambiano V, Bruun T, Vernazza P, Collins S, et al. (2016) Sexual Activity Without Condoms and Risk of HIV Transmission in Serodifferent Couples When the HIV-Positive Partner Is Using Suppressive Antiretroviral Therapy. Jama-Journal of the American Medical Association 316: 171-181.
17. Rodger A BT, Cambiano V, Vernazza P, Strada V, Van Lunzen J. (2014) 153LB: HIV transmission risk through condomless sex if HIV positive partner is on supressive ART: PARTNER study. . Boston.
18. Nosyk B, Zang X, Min JE, Krebs E, Lima VD, et al. (2017) Relative effects of antiretroviral therapy and harm reduction initiatives on HIV incidence in British Columbia, Canada, 1996-2013: a modelling study. The lancet HIV 4: E303-E310.
19. Long EF, Brandeau ML, Owens DK (2010) The Cost-Effectiveness and Population Outcomes of Expanded HIV Screening and Antiretroviral Treatment in the United States. Annals of Internal Medicine 153: 778-+.
20. Long EF, Brandeau ML, Galvin CM, Vinichenko T, Tole SP, et al. (2006) Effectiveness and cost-effectiveness of strategies to expand antiretroviral therapy in St. Petersburg, Russia. Aids 20: 2207-2215.
21. Kaplan EH, Heimer R (1992) A Model-Based Estimate of Hiv Infectivity Via Needle Sharing. Journal of Acquired Immune Deficiency Syndromes and Human Retrovirology 5: 1116-1118.
22. Wall SD, Olcott EW, Gerberding JL (1991) Aids Risk and Risk Reduction in the Radiology Department. American Journal of Roentgenology 157: 911-917.
23. Hudgens MG, Longini IM, Vanichseni S, Hu DJ, Kitayaporn D, et al. (2002) Subtype-specific transmission probabilities for human immunodeficiency virus type 1 among injecting drug users in Bangkok, Thailand. American Journal of Epidemiology 155: 159-168.
24. Baggaley RF, Boily MC, White RG, Alary M (2006) Risk of HIV-1 transmission for parenteral exposure and blood transfusion: a systematic review and meta-analysis. Aids 20: 805-812.
25. Patel P, Borkowf CB, Brooks JT, Lasry A, Lansky A, et al. (2014) Estimating per-act HIV transmission risk: a systematic review. Aids 28: 1509-1519.
26. Quinn TC, Wawer MJ, Sewankambo N, Serwadda D, Li CJ, et al. (2000) Viral load and heterosexual transmission of human immunodeficiency virus type 1. New England Journal of Medicine 342: 921-929.
27. Downs AM, DeVincenzi I, Costigliola P, Ricchi E, Chiodo F, et al. (1996) Probability of heterosexual transmission of HIV: Relationship to the number of unprotected sexual contacts. Journal of Acquired Immune Deficiency Syndromes and Human Retrovirology 11: 388-395.
28. Hollingsworth TD, Anderson RM, Fraser C (2008) HIV-1 transmission, by stage of infection. Journal of Infectious Diseases 198: 687-693.
29. Kaplan EH (1990) Modeling Hiv Infectivity - Must Sex Acts Be Counted. Journal of Acquired Immune Deficiency Syndromes and Human Retrovirology 3: 55-61.
30. Mastro TD, de Vincenzi I (1996) Probabilities of sexual HIV-1 transmission. Aids 10 Suppl A: S75-82.
31. Nicolosi A, Leite MLC, Musicco M, Arici C, Gavazzeni G, et al. (1994) The Efficiency of Male-to-Female and Female-to-Male Sexual Transmission of the Human-Immunodeficiency-Virus - a Study of 730 Stable Couples. Epidemiology 5: 570-575.
32. Caceres CF, Vangriensven GJP (1994) Male-Homosexual Transmission of Hiv-1. Aids 8: 1051-1061.

33. Jacquez JA, Koopman JS, Simon CP, Longini IM (1994) Role of the Primary Infection in Epidemics of Hiv-Infection in Gay Cohorts. Journal of Acquired Immune Deficiency Syndromes and Human Retrovirology 7: 1169-1184.
34. Vittinghoff E, Douglas J, Judson F, McKirnan D, MacQueen K, et al. (1999) Per-contact risk of human immunodeficiency virus transmission between male sexual partners. American Journal of Epidemiology 150: 306-311.
35. Boily MC, Baggaley RF, Wang L, Masse B, White RG, et al. (2009) Heterosexual risk of HIV-1 infection per sexual act: systematic review and meta-analysis of observational studies. Lancet Infectious Diseases 9: 118-129.
36. Peterman TA, Stoneburner RL, Allen JR, Jaffe HW, Curran JW (1988) Risk of Human Immunodeficiency Virus Transmission from Heterosexual Adults with Transfusion-Associated Infections. Jama-Journal of the American Medical Association 259: 55-58.
37. Obrien TR, Busch MP, Donegan E, Ward JW, Wong LY, et al. (1994) Heterosexual Transmission of Human-Immunodeficiency-Virus Type-1 from Transfusion Recipients to Their Sex Partners. Journal of Acquired Immune Deficiency Syndromes and Human Retrovirology 7: 705-710.
38. Shiboski SC, Padian NS (1998) Epidemiologic evidence for time variation in HIV infectivity. Journal of Acquired Immune Deficiency Syndromes and Human Retrovirology 19: 527-535.
39. Padian NS, Shiboski SC, Glass SO, Vittinghoff E (1997) Heterosexual transmission of human immunodeficiency virus (HIV) in Northern California: Results from a ten-year study. American Journal of Epidemiology 146: 350-357.
40. Leynaert B, Downs AM, de Vincenzi I, H ESGHT (1998) Heterosexual transmission of human immunodeficiency virus - Variability of infectivity throughout the course of infection. American Journal of Epidemiology 148: 88-96.
41. de Vincenzi I (1994) A longitudinal study of human immunodeficiency virus transmission by heterosexual partners. European Study Group on Heterosexual Transmission of HIV. The New England journal of medicine 331: 341-346.
42. Attia S, Egger M, Muller M, Zwahlen M, Low N (2009) Sexual transmission of HIV according to viral load and antiretroviral therapy: systematic review and meta-analysis. Aids 23: 1397-1404.
43. Powers KA, Poole C, Pettifor AE, Cohen MS (2008) Rethinking the heterosexual infectivity of HIV-1: a systematic review and meta-analysis. Lancet Infectious Diseases 8: 553-563.
44. Baggaley RF, White RG, Boily MC (2010) HIV transmission risk through anal intercourse: systematic review, meta-analysis and implications for HIV prevention. International Journal of Epidemiology 39: 1048-1063.
45. Degruttola V, Seage GR, Mayer KH, Horsburgh CR (1989) Infectiousness of Hiv between Male-Homosexual Partners. Journal of Clinical Epidemiology 42: 849-856.
46. Grant RM, Wiley JA, Winkelstein W (1987) Infectivity of the Human-Immunodeficiency-Virus - Estimates from a Prospective-Study of Homosexual Men. Journal of Infectious Diseases 156: 189-193.
47. Blaser N, Wettstein C, Estill J, Vizcaya LS, Wandeler G, et al. (2014) Impact of viral load and the duration of primary infection on HIV transmission: systematic review and meta-analysis. Aids 28: 1021-1029.
48. Suthar AB, Granich RM, Kato M, Nsanzimana S, Montaner JSG, et al. (2015) Programmatic Implications of Acute and Early HIV Infection. Journal of Infectious Diseases 212: 1351-1360.

49. Fiebig EW, Wright DJ, Rawal BD, Garrett PE, Schumacher RT, et al. (2003) Dynamics of HIV viremia and antibody seroconversion in plasma donors: implications for diagnosis and staging of primary HIV infection. Aids 17: 1871-1879.
50. Bellan SE, Dushoff J, Galvani AP, Meyers LA (2015) Reassessment of HIV-1 Acute Phase Infectivity: Accounting for Heterogeneity and Study Design with Simulated Cohorts. Plos Medicine 12.
51. Wawer MJ, Gray RH, Sewankambo NK, Serwadda D, Li XB, et al. (2005) Rates of HIV-1 transmission per coital act, by stage of HIV-1 infection, in Rakai, Uganda. Journal of Infectious Diseases 191: 1403-1409.
52. Pinkerton SD (2008) Probability of HIV transmission during acute infection in Rakai, Uganda. Aids and Behavior 12: 677-684.
53. Escudero DJ, Lurie MN, Mayer KH, Weinreb C, King M, et al. (2016) Acute HIV infection transmission among people who inject drugs in a mature epidemic setting. Aids 30: 2537-2544.
54. (1998) The NIMH Multisite HIV Prevention Trial: reducing HIV sexual risk behavior. The National Institute of Mental Health (NIMH) Multisite HIV Prevention Trial Group. Science 280: 1889-1894.
55. Kamb ML, Fishbein M, Douglas JM, Rhodes F, Rogers J, et al. (1998) Efficacy of risk-reduction counseling to prevent human immunodeficiency virus and sexually transmitted diseases - A randomized controlled trial. Jama-Journal of the American Medical Association 280: 1161-1167.
56. Cleary PD, Vandevanter N, Rogers TF, Singer E, Shiptonlevy R, et al. (1991) Behavior Changes after Notification of Hiv-Infection. American Journal of Public Health 81: 1586-1590.
57. Higgins DL, Galavotti C, Oreilly KR, Schnell DJ, Moore M, et al. (1991) Evidence for the Effects of Hiv Antibody Counseling and Testing on Risk Behaviors. Jama-Journal of the American Medical Association 266: 2419-2429.
58. Neumann MS, Johnson WD, Semaan S, Flores SA, Peersman G, et al. (2002) Review and meta-analysis of HIV prevention intervention research for heterosexual adult populations in the United States. Journal of acquired immune deficiency syndromes 30: S106-S117.
59. Crepaz N, Marshall KJ, Aupont LW, Jacobs ED, Mizuno Y, et al. (2009) The Efficacy of HIV/STI Behavioral Interventions for African American Females in the United States: A Meta-Analysis. American Journal of Public Health 99: 2069-2078.
60. Henny KD, Crepaz N, Lyles CM, Marshall KJ, Aupont LW, et al. (2012) Efficacy of HIV/STI Behavioral Interventions for Heterosexual African American Men in the United States: A Meta-Analysis. Aids and Behavior 16: 1092-1114.
61. Marks G, Crepaz N, Senterfitt JW, Janssen RS (2005) Meta-analysis of high-risk sexual behavior in persons aware and unaware they are infected with HIV in the United States - Implications for HIV prevention programs. Jaids-Journal of Acquired Immune Deficiency Syndromes 39: 446-453.
62. United States Census Bureau (2016) April 1 2010 to July 1 2015 – United States – Metropolitan and micropolitan statistical area; and Puerto Rico. United States.
63. Tempalski B, Pouget E, Cleland C, Brady J, Cooper H, et al. (2013) Trends in the population prevalence of people who inject drugs in US metropolitan areas 1992-2007. PLoS One 8: e64789.
64. Grey JA, Bernstein KT, Sullivan PS, Purcell DW, Chesson HW, et al. (2016) Estimating the Population Sizes of Men Who Have Sex With Men in US States and Counties Using Data From the American Community Survey. JMIR Public Health and Surveillance 2: e14.

65. Centers for Disease Control and Prevention (2016) HIV Infection Risk, Prevention, and Testing Behaviors among Men Who Have Sex With Men—National HIV Behavioral Surveillance, 20 U.S. Cities, 2014. HIV Surveillance Special Report 15. .
66. Centers for Disease Control and Prevention (2014) HIV Infection and Risk, Prevention, and Testing Behaviors Among Injecting Drug Users — National HIV Behavioral Surveillance System, 20 U.S. Cities, 2009.
67. County of Los Angeles (2011) 2010 Annual HIV Surveillance Report. Los Angeles: Public Health LA County.
68. Florida Department of Health in Miami-Dade County (2015) HIV Surveillance.
69. New York City Department of Health and Mental Hygiene (2011) New York City HIV/AIDS Annual Surveillance Statistics 2010. New York City: New York City Department of Health and Mental Hygiene.
70. Public Health - Seattle and King County (2018) HIV/AIDS annual and quarterly reports.
71. Center for HIV Surveillance EaE, Department of Health and Mental Hygiene, Baltimore, MD., (2015) Baltimore City Annual HIV Epidemiological Profile 2013.
72. Georgia Department of Public Health (2012) HIV/AIDS Epidemiology Program HIV Care Continuum Surveillance Report.
73. Centers for Disease Control and Prevention (2015) Prevalence of Diagnosed and Undiagnosed HIV infection - United States, 2008-2012. Morbidity and mortality weekly report (MMWR) 64: 657-662.
74. Song R, Hall HI, Green TA, Szwarcwald CL, Pantazis N (2017) Using CD4 Data to Estimate HIV Incidence, Prevalence, and Percent of Undiagnosed Infections in the United States. J Acquir Immune Defic Syndr 74: 3-9.
75. Long E, Mandalia R, Mandalia S, Alistar S, Beck E, et al. (2014) Expanded HIV testing in low-prevalence, high-income countries: A cost-effectiveness analysis for the United Kingdom. PLoS One 9: e95735.
76. The HIV Research Network (HIVRN) Goals of the HIV Research Network. .
77. Harrison KM, Kajese T, Hall HI, Song R (2008) Risk factor redistribution of the national HIV/AIDS surveillance data: an alternative approach. Public Health Rep 123: 618-627.
78. Günthard HF, Saag MS, Benson CA, et al. (2016) Antiretroviral drugs for treatment and prevention of hiv infection in adults: 2016 recommendations of the international antiviral society–usa panel. JAMA 316: 191-210.
79. Atlanta Regional Commission (2008) ARC Fifty Forward Metro Atlanta: Demography & Diversity.
80. Maryland Department of Planning (2018) Total Population Projections by Age, Sex and Race.
81. State of California - Department of Finance (2018) County Population Projections (2010-2060).
82. University of Florida - Bureau of Economic and Business Research (2017) Population Projections by Age, Sex, Race, and Hispanic Origin for Florida and Its Counties, 2020–2045, With Estimates for 2017.
83. City of New York - Department of City Planning (2013) New York City Population Projections by Age/Sex & Borough, 2010-2040.
84. Centers for Disease Control and Prevention - CDC Wonder (2018) National Birth, Death, Migration Projections by Race 2014-2060 Request.
85. Washington State Office of Financial Management (2017) Projections of the state population by age, sex, race and Hispanic origin.
86. Administration. DoHaMHVS (2010) Maryland Vital Statistics Annual Report 2010

87. Los Angeles County Department of Public Health (2013) Mortality in Los Angeles County 2010.
88. New York City Department of Health and Mental Hygiene (2011) Summary of Vital Statistics 2010: The City of New York.
89. Florida Department of Health (2018) All Causes Crude Death Rate 2010.
90. Washington State Department of Health (2011) Age-Adjusted Mortality Rates.
91. Georgia Department of Public Health (2018) Age-Adjusted Death Rate 2010.
92. (HIVRN). HRN Goals of the HIV Research Network. .
93. Mangal TD (2017) Joint estimation of CD4+ cell progression and survival in untreated individuals with HIV-1 infection. Aids 31: 1073-1082.
94. Bernard CL, Brandeau ML, Humphreys K, Bendavid E, Holodniy M, et al. (2016) Cost-Effectiveness of HIV Preexposure Prophylaxis for People Who Inject Drugs in the United States. Annals of Internal Medicine 26: M15-2634.
95. Nosyk B, Min JE, Evans E, Li L, Liu L, et al. (2015) The effects of Opioid Substitution Treatment and Highly Active Antiretroviral Therapy on the cause-specific risk of mortality among HIV-positive people who inject drugs. Clin Infect Dis 61: 1157-1165.
96. Wang L, Krebs E, Min JE, Mathews W, Nijhawan A, et al. (2018) Combined estimation of disease progression and antiretroviral therapy retention among treated individuals with HIV in the United States. Submitted.
97. Evans E, Li L, Min J, Huang D, Urada D, et al. (2015) Mortality among individuals accessing pharmacological treatment for opioid dependence in California, 2006-10. Addiction 110: 996-1005.
98. Centers for Disease Control and Prevention (2014) Behavioral Risk Factor Surveillance System Survey Data. Atlanta, GA: Centers for Disease Control and Prevention.
99. New York City Department of Health and Mental Hygiene (2014) Community Health Survey.
100. Centers for Disease Control and Prevention (2014) Public use data file documentation. 2011-2013. National Survey of Family Growth. User's guide. Hyattsville, Maryland: Centers for Disease Control and Prevention, National Center for Health Science.
101. Vlahov D, Anthony JC, Munoz A, Margolick J, Nelson KE, et al. (1991) The Alive Study - a Longitudinal-Study of Hiv-1 Infection in Intravenous-Drug-Users - Description of Methods. Journal of Drug Issues 21: 759-776.
102. Metsch LR, Feaster DJ, Gooden L, Schackman BR, Matheson T, et al. (2013) Effect of risk-reduction counseling with rapid HIV testing on risk of acquiring sexually transmitted infections: the AWARE randomized clinical trial. Journal of the American Medical Association 310: 1701-1710.
103. Centers for Disease Control and Prevention (2014) HIV Risk, Prevention, and Testing Behaviors National HIV Behavioral Surveillance System Men Who Have Sex with Men 20 U.S. Cities, 2011, HIV Surveillance Special Report 8. .
104. Barbee LA, Khosropour CM, Dombrowksi JC, Golden MR (2017) New Human Immunodeficiency Virus Diagnosis Independently Associated With Rectal Gonorrhea and Chlamydia in Men Who Have Sex With Men. Sex Transm Dis 44: 385-389.
105. Looker KJ, Elmes JAR, Gottlieb SL, Schiffer JT, Vickerman P, et al. (2017) Effect of HSV-2 infection on subsequent HIV acquisition: an updated systematic review and meta-analysis. Lancet Infectious Diseases 17: 1303-1316.
106. Centers for Disease Control and Prevention (2015) HIV Infection, Risk, Prevention, and Testing Behaviors among Persons Who Inject Drugs—National HIV Behavioral

Surveillance: Injection Drug Use, 20 U.S. Cities, 2012. HIV Surveillance Special Report 11. Revised edition. .
107. Fujimoto K, Wang P, Ross MW, Williams ML (2015) Venue-Mediated Weak Ties in Multiplex HIV Transmission Risk Networks Among Drug-Using Male Sex Workers and Associates. American Journal of Public Health 105: 1128-1135.
108. Raymond HF, McFarland W (2009) Racial Mixing and HIV Risk Among Men Who Have Sex with Men. Aids and Behavior 13: 630-637.
109. Mellors JW, Munoz A, Giorgi JV, Margolick JB, Tassoni CJ, et al. (1997) Plasma viral load and CD4+ lymphocytes as prognostic markers of HIV-1 infection. Annals of Internal Medicine 126: 946-954.
110. Sanders GD, Bayoumi AM, Sundaram V, Bilir SP, Neukermans CP, et al. (2005) Cost-effectiveness of screening for HIV in the era of highly active antiretroviral therapy. New England Journal of Medicine 352: 570-585.
111. Gaines TL, Caldwell JT, Ford CL, Mulatu MS, Godette DC (2016) Relationship between a Centers for Disease Control and Prevention expanded HIV testing initiative and past-year testing by race/ethnicity: a multilevel analysis of the Behavioral Risk Factor Surveillance System. AIDS Care 28: 554-560.
112. Centers for Disease Control and Prevention (2016) Behavioral and Clinical Characteristics of Persons Receiving Medical Care for HIV Infection — Medical Monitoring Project, United States.
113. Wang LK, E.; Min, J.E.; Mathews, W.; Nijhawan, A.; Somboonwit, C; Aberg, J.A.; Moore, R.D.; Gebo, K.A.; Nosyk, B. (2018) Combined Estimation of Disease Progression and Antiretroviral Therapy Retention Among Treated Individuals with HIV in the United States. Submitted.
114. Georgia Health News (2017) Injectable drugs can kill, but clean syringes can save lives.
115. City of Baltimore (2016) Baltimore City Syringe Exchange Program.
116. City of Los Angeles (2014) AIDS Coordinator's Office.
117. Centers for Disease Control and Prevention (2015) Syringe Service Programs for Persons Who Inject Drugs in Urban, Suburban, and Rural Areas — United States, 2013.
118. New York State Department of Health AIDS Institute (2014) Comprehensive Harm Reduction Reverses the Trend in New HIV Infections.
119. Public Health - Seattle & King County (2017) HIV/STD Program.
120. Aspinall EJ, Nambiar D, Goldberg DJ, Hickman M, Weir A, et al. (2013) Are needle and syringe programmes associated with a reduction in HIV transmission among people who inject drugs: a systematic review and meta-analysis. International Journal of Epidemiology 43: 235-248.
121. SAMHSA (2018) Treatment Episode Data Set (TEDS).
122. SAMHSA (2017) Number of DATA-Certified Physicians.
123. Hansen HB, Siegel CE, Case BG, Bertollo DN, DiRocco D, et al. (2013) Variation in use of buprenorphine and methadone treatment by racial, ethnic, and income characteristics of residential social areas in New York City. J Behav Health Serv Res 40: 367-377.
124. Saloner B, Daubresse M, Caleb Alexander G (2017) Patterns of Buprenorphine-Naloxone Treatment for Opioid Use Disorder in a Multistate Population. Med Care 55: 669-676.
125. Timko C, Schultz NR, Cucciare MA, Vittorio L, Garrison-Diehn C (2016) Retention in medication-assisted treatment for opiate dependence: A systematic review. J Addict Dis 35: 22-35.

126. Low AJ, Mburu G, Welton NJ, May MT, Davies CF, et al. (2016) Impact of Opioid Substitution Therapy on Antiretroviral Therapy Outcomes: A Systematic Review and Meta-Analysis. Clin Infect Dis 63: 1094-1104.
127. MacArthur GJ, Minozzi S, Martin N, Vickerman P, Deren S, et al. (2012) Opiate substitution treatment and HIV transmission in people who inject drugs: systematic review and meta-analysis. Bmj 3.
128. Mera R, Magnuson D, Trevor H, Bush S, Rawlings K, et al. Changes in Truvada (TVD) for HIV pre-exposure prophylaxis (PrEP) utilisation in the United States:(2012-2016); 2017.
129. AIDS info (2012) FDA approves first drug for reducing the risk of sexually acquired HIV infection.
130. Emory University - Rollins School of Public Health (2015) AIDSVu - ZIP3 PrEP Data Sets.
131. Sullivan PS, Giler RM, Mouhanna F, Pembleton ES, Guest JL, et al. (2018) Trends in the use of oral emtricitabine/tenofovir disoproxil fumarate for pre-exposure prophylaxis against HIV infection, United States, 2012-2017. Ann Epidemiol 28: 833-840.
132. AIDS Institute: NYS Department of Health (2016) PrEP Measurement in NYS.
133. Lieb S, Fallon S, Friedman S, Thompson D, Gates G, et al. (2011) Statewide estimation of racial/ethnic populations of men who have sex with men in the US. Public Health Rep 126: 60-72.
134. Centers for Disease Control and Prevention (2014) Preexposure Prophylaxis for the prevention of HIV infection in the United States.
135. Anderson PL, Glidden DV, Liu A, Buchbinder S, Lama JR, et al. (2012) Emtricitabine-Tenofovir Concentrations and Pre-Exposure Prophylaxis Efficacy in Men Who Have Sex with Men. Science Translational Medicine 4: 151ra125.
136. Liu AY, Cohen SE, Vittinghoff E, Anderson PL, Doblecki-Lewis S, et al. (2016) Preexposure Prophylaxis for HIV Infection Integrated With Municipal- and Community-Based Sexual Health Services. JAMA Intern Med 176: 75-84.
137. Centers for Medicare and Medicaid Services (2017) Physician fee schedule search.
138. United States Department of Veterans Affairs - Office of Procurement Acquisition and Logistics (OPAL) (2018) Pharmaceutical Prices.
139. Centers for Medicare and Medicaid Services (2017) Clinical laboratory fee schedule.
140. McCollister K, Yang X, Sayed B, French MT, Leff JA, et al. (2017) Monetary conversion factors for economic evaluations of substance use disorders. J Subst Abuse Treat 81: 25-34.
141. Enns B, Krebs E, Min JE, Mathews W, Moore RD, et al. (2018) Heterogeneity in the costs of medical care among people living with HIV/AIDS in the United States. Submitted.
142. Agency for Healthcare Research and Quality (2018) Mean expenditure per person by census region, United States, 1996-2015. Medical Expenditure Panel Survey Household Component Data.
143. Tookes H, Diaz C, Li H, Khalid R, Doblecki-Lewis S (2015) A Cost Analysis of Hospitalizations for Infections Related to Injection Drug Use at a County Safety-Net Hospital in Miami, Florida. PLoS One 10.
144. Baser O, Chalk M, Fiellin DA, Gastfriend DR (2011) Cost and utilization outcomes of opioid-dependence treatments. American Journal of Managed Care 17: S235-248.
145. Holtgrave DR, Pinkerton SD (1997) Updates of cost of illness and quality of life estimates for use in economic evaluations of HIV prevention programs. JAIDS Journal of Acquired Immune Deficiency Syndromes 16: 54-62.

146. Honiden S, Sundaram V, Nease RF, Holodniy M, Lazzeroni LC, et al. (2006) The effect of diagnosis with HIV infection on health-related quality of Life. Quality of Life Research 15: 69-82.
147. Schackman BR, Goldie SJ, Freedberg KA, Losina E, Brazier J, et al. (2002) Comparison of health state utilities using community and patient preference weights derived from a survey of patients with HIV/AIDS. Med Decis Making 22: 27-38.
148. Tengs TO, Lin TH (2002) A meta-analysis of utility estimates for HIV/AIDS. Med Decis Making 22: 475-481.
149. Joyce VR, Barnett PG, Bayoumi AM, Griffin SC, Kyriakides TC, et al. (2009) Health-related quality of life in a randomized trial of antiretroviral therapy for advanced HIV disease. Journal of Acquired Immune Deficiency Syndromes 50: 27-36.
150. Kauf TL, Roskell N, Shearer A, Gazzard B, Mauskopf J, et al. (2008) A predictive model of health state utilities for HIV patients in the modern era of highly active antiretroviral therapy. Value in Health 11: 1144-1153.
151. Song DL, Altice FL, Copenhaver MM, Long EF (2015) Cost-effectiveness analysis of brief and expanded evidence-based risk reduction interventions for HIV-infected people who inject drugs in the United States. PLoS One 10.
152. Newman MEJ (2003) Mixing patterns in networks. Physical Review E 67.
153. Medical Monitoring Project (2013) Medical Monitoring Project 2014 Protocol.
154. Medical Monitoring Project (2009) Medical Monitoring Project 2010 Protocol.
155. Gfroerer J, Bose J, Trunzo D, Strashny A, Batts K, et al. (2014) Estimating Substance Abuse Treatment: A Comparison of Data from a Household Survey, a Facility Survey, and an Administrative Data Set. Substance Abuse and Mental Health Administration.
156. Zang X, Krebs E, Min J, Marshall B, Granich R, et al. (2018) Development and calibration of a dynamic HIV transmission model for 6 US cities. Pre-submission.
157. NYC Health (2015) HIV/AIDS Surveillance Data.
158. County of Los Angeles Public Health (2018) LA Health Data Now!
159. Maryland Department of Health (2018) HIV Statistics.
160. Public Health - Seattle & King County (2018) HIV/AIDS annual and quarterly reports.
161. Florida Department of Health in Miami-Dade County (2016) HIV Cases Diagnosed from 2013 to 2015.
162. Georgia Department of Public Health - Office of HIV/AIDS (2016) Georgia Integrated HIV Prevention & Care Plan 2017-2021.
163. Georgia Department of Public Health (2018) Georgia's HIV/AIDS Epidemiology Surveillance Section.
164. Emory University - Rollins School of Public Health (2018) AIDSVu.
165. Florida Department of Health - Division of Public Health Statistics & Performance Management (2018) HIV/AIDS Deaths.
166. Washington State Department of Health (2015) Washington State HIV Surveillance Semiannual Report 2nd Edition 2015.
167. Centers for Disease Control and Prevention (2017) NCHHSTP AtlasPlus.
168. Briggs AH, Claxton K, Sculpher MJ (2006) Decision modelling for health economic evaluation: Handbooks in Health Economic E.
169. Briggs AH, Goeree R, Blackhouse G, O’Brien BJ (2002) Probabilistic analysis of cost-effectiveness models: choosing between treatment strategies for gastroesophageal reflux disease. Medical Decision Making 22: 290-308.
