# Supplementary material for: Developing a dynamic HIV transmission model for 6 U.S. cities: An evidence synthesis
Source: PLoS One. 2019 May 30;14(5):e0217559. doi: 10.1371/journal.pone.0217559 (PMC6542533; doi:10.1371/journal.pone.0217559)
Supplement: S2 Supplement — Supplement Tables Supporting information includes (i) excel file of values, ranges and PSA distributions for all model parameters, (ii) excel file of values and ranges for calibration/validation targets for all cities, and (iii) pdf of full survey given to SAC members for data verification. (ZIP) [file pone.0217559.s002.zip › CascadeCEA_EvidenceSynthesis_SupplementTables/CascadeCEA_EvidenceSynthesis_Supplement F_SAC survey.pdf]

Supplement F. Scientific Advisory Committee Survey on SurveyMonkey®

Localized Economic Modeling: Scientific Advisory Committee Survey

ABOUT THIS SURVEY

Thank you for taking the time to complete this 45-60 minute survey to support the evidence synthesis component of our project. The survey has been designed to ensure we capture the best possible data available for each city, and also elicit your expert judgement on various assumptions we have had to make to estimate parameter values in our model.

We will be sharing some data points, ranges and estimation techniques for parameters in 4 key domains of our model:

1. **Population size estimates for HIV risk groups.**
2. **The force of HIV infection.**
3. **Antiretroviral therapy (ART) engagement and disease progression.**
4. **Health system engagement.**

You will be asked three kinds of questions:

1. **To identify any additional data sources.**
2. **To rate your confidence in the assumptions used to estimate parameter values. Hypothetical examples will be provided to demonstrate the implications of each assumption.**
3. **To explain why you rated different assumptions/methods the way you did.**

* 1. For which city would like to complete this survey? (Please select only one city for which you have expertise on the HIV/AIDS epidemic)

- ◯ Atlanta, GA
- ◯ Baltimore, MD
- ◯ Los Angeles, CA
- ◯ Miami, FL
- ◯ New York City, NY
- ◯ Seattle, WA

* 2. Please provide your contact information so we may follow up with you on your responses.

**Name**	
**Organization**	
**City/Town**	
**State/Province**	-- select state --
**Email Address**	

SECTION 1: HIV RISK GROUP POPULATION ESTIMATES

In this section, we will ask you to consider city-level estimates for the population size of adults (15-64) within our model's four HIV transmission risk groups:

1. Men who have sex with men (MSM)
2. People who inject drugs (PWID)
3. Men who have sex with men and inject drugs (MSM-PWID)
4. Heterosexuals (HET)

You will be asked to assess data sources and assumptions used to estimate the city-level population size of HIV negative and HIV positive adults within each risk group, stratified by race/ethnicity and gender.

We will also ask you to rank your confidence in assumptions used to:
1. Cross tabulate diagnosed PLHIV by race/ethnicity and transmission risk group
2. Distribute "other" transmission categories to the model's four HIV transmission risk groups.

The purpose of the figure below is to clarify the terminologies that will be used throughout this survey. As an example, the phrase "MSM population size" will be used to refer to the general MSM population, regardless of their HIV status. This figure will appear before each sub-section to identify which group we are asking about.

Population Size of People Who Inject Drugs (PWID)

On the next page, you will be asked to consider estimates for the population size of <u>all</u> adult (15-64) people who use drugs, which includes those who are HIV positive <u>and</u> HIV negative.

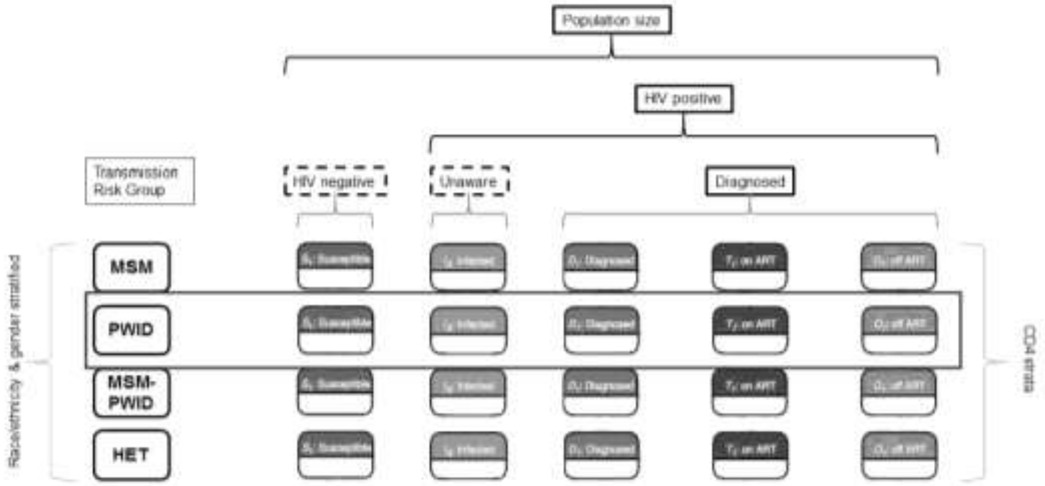

Population Size of People Who Inject Drugs (PWID)

To estimate the adult (15-64) population size of PWID in each city, we use Tempalski and colleagues 2007 MSA-level estimates of PWID prevalence, which are stratified by race/ethnicity.

	Population prevalence of PWID, 2007			
**City**	**Combined**	**White**	**Black/African American**	**Hispanic/Latino**
Los Angeles, CA	1.0% (0.5-2.0%)	1.2% (0.5-2.3%)	2.4 %(1.1-4.6%)	1.0% (0.5-2.0%)
Miami, FL	0.5% (0.2-0.8%)	1.4% (0.5-2.3%)	0.6% (0.2-1.0%)	0.2% (0.1-0.3%)
Atlanta, GA	0.6% (0.3-0.7%)	0.5% (0.3-0.7%)	0.8% (0.4-1.0%)	0.2% (0.1-0.3%)
Baltimore, MD	3.2% (1.4-4.8%)	1.9% (0.8-2.8%)	6.3% (2.7-9.3%)	1.8% (0.8-2.7%)
New York City, NY	1.6% (0.8-2.6%)	0.8% (0.4-1.3%)	2.1% (1.0-3.3%)	3.0% (1.5-4.9%)
Seattle, WA	1.5% (1.0-2.6%)	1.2% (0.8-2.0%)	5.2% (3.4-8.8%)	1.5% (1.0-2.5%)

Source: Tempalski B, et al. Trends in the population prevalence of people who inject drugs in US metropolitan areas 1992-2007. PLoS One. 2013;8(6):e64789

3. Are you aware of <u>more recent</u> data to estimate the population size of PWID in {{ Q1 }}?

- ◯ No
- ◯ Yes (please specify)

4. Using a scale of 1 (not at all confident) to 5 (completely confident), please rate how confident you are in the following methods used to distribute PWID by gender within each race/ethnicity in {{ Q1 }}.

	1<br>Not at all confident	2<br>Not very confident	3<br>Neutral	4<br>Somewhat confident	5<br>Completely confident
**1. Assume distribution is representative of city-level gender distribution of the general population**<br>If 50% of Black adults in Los Angeles are male, then 50% of the Black PWID population in LA will be male	○	○	○	○	○
**2. Assume distribution is representative of national-level gender distribution of PWID**<br>If 70% of the Black PWID population in the US is male, then 70% of the Black PWID population in Los Angeles is male	○	○	○	○	○

5. Please tell us more about why you rated these methods the way you did.

Population Size of Men Who Have Sex With Men and Inject Drugs (MSM-PWID)

On the next page, you will be asked to consider estimates for the population size of all adult (15-64) men who have sex with men and inject drugs, which includes those who are HIV positive and HIV negative.

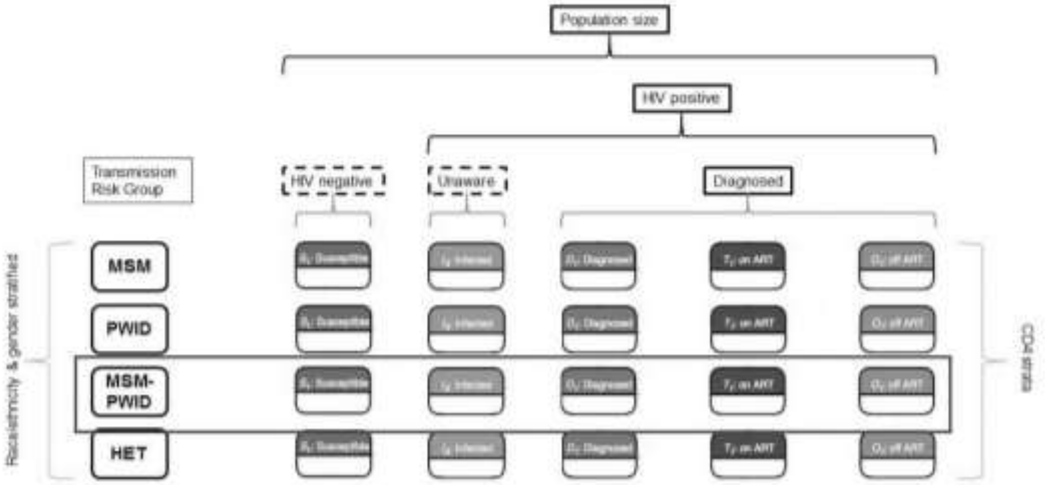

Population Size of Men Who Have Sex with Men and Inject Drugs (MSM-PWID)

To estimate the population size of adult (15-64) MSM-PWID, we use 2010 CDC national-level estimates for the percentage of men who *have ever* had sex with a man and *have ever* injected drugs, stratified by race/ethnicity.

Race/Ethnicity	Percentage of PWID who are MSM-PWID[1]	Percentage of adult males who are MSM-PWID[2]
Overall	0.100-0.104	6.9% (3.6-19.9%)
White	0.119-0.140	
Black/African American	0.070-0.086	
Hispanic/Latino	0.115-0.120	

1. Broz, D., et al. (2014). "HIV infection and risk, prevention, and testing behaviors among injecting drug users - National HIV Behavioral Surveillance System, 20 US cities, 2009." MMWR 63(ss06): 1-51.

2. Centers for Disease Control and Prevention (2015). HIV infection, risk, prevention, and testing behaviors among persons who inject drugs - National HIV behavioral surveillance: Injection Drug Use, 20 US Cities, 2012. HIV Surveillance Special Report 11. Atlanta, GA, Centers for Disease Control and Prevention.

6. Are you aware of city-level data to estimate the size of the MSM-PWID population in {{ Q1 }}?

- ◯ No
- ◯ Yes (please specify)

7. Using a scale from 1 (not at all confident) to 5 (completely confident), please rate how confident you are in the following methods used to estimate the proportion of males who are MSM-PWID by race/ethnicity in {{ Q1 }}.

	1<br>Not at all confident	2<br>Not very confident	3<br>Neutral	4<br>Somewhat confident	5<br>Completely confident
**1. Representative of national race/ethnicity specific estimates for proportion of male PWID who have sex with men.**<br>If 5% of the White male PWID population in the US has sex with men, then 5% of New York's white male PWID population has sex with men					
**2. Representative of city-level estimates for males who are MSM**<br>If 8% of New York's MSM population injects drugs, then 8% of White MSM in New York inject drugs					
**3. Representative of city-level estimates for males who are PWID**<br>If 15% of New York's males who inject drugs have sex with men, then 15% of the White male PWID in New York have sex with men					

8. Please tell us more about why you rated these methods the way you did.

Size of the HIV positive MSM population

On the next page, you will be asked to consider estimates for the population size of HIV positive adult (15-64) men who have sex with men. This population includes both those who are unaware of their status <u>and</u> those who are diagnosed.

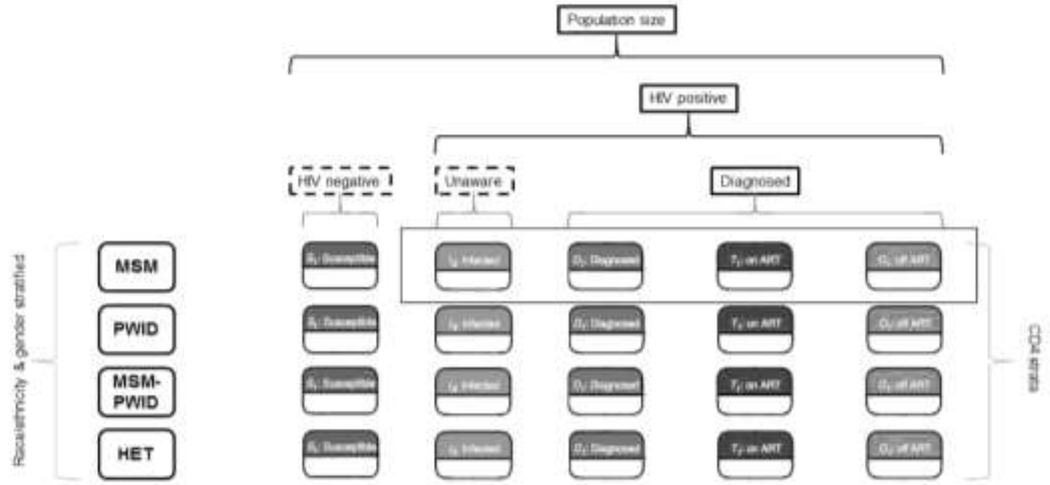

Size of the HIV Positive MSM population

To estimate the population size of HIV Positive MSM in each city, we use 2008 CDC estimates on the prevalence of HIV infection among MSM .

City	HIV prevalence among MSM
Los Angeles, CA	19.0% (15.1-22.0%)
Miami, FL	25.0% (22.0-29.0%)
Atlanta, GA	6.0% (4.0-10.0%)
Baltimore, MD	38.0% (33.0-43.0%)
New York City, NY	29.0% (25.0-33.0%)
Seattle, WA	15.0% (11.0-19.0%)

Source: Centers for Disease Control and Prevention. Prevalence and awareness of HIV infection among men who have sex with men - 21 cities, United States, 2008. MMWR. 2010;59(37):1201-28.

9. Are you aware of more recent data to estimate HIV prevalence among MSM in {{ Q1 }}?

◯ No

◯ Yes (please specify)

10. Using a scale from 1 (not at all confident) to 5 (completely confident), please rate how confident are you in the following methods to estimate the proportion of MSM who are HIV positive by race/ethnicity in {{ Q1 }}.

	1 Not at all confident	2 Not very confident	3 Neutral	4 Somewhat confident	5 Completely confident
1. **Equal proportion of MSM who are HIV positive across race/ethnicities** If 25% of MSM in Miami are estimated to be HIV positive, then 25% of Hispanic/Latino MSM in Miami are HIV positive	◯	◯	◯	◯	◯
2. **Representative of city-level cumulative diagnoses of HIV among MSM** If 30% of Miami's cumulative diagnoses of HIV among MSM are Hispanic/Latino, then 30% of MSM who are HIV positive in Miami are Hispanic/Latino.	◯	◯	◯	◯	◯
3. **Representative of city-level recent (last year) diagnoses of HIV among MSM** If 20% of Miami's last-year diagnoses of HIV among MSM are Hispanic/Latino, then 20% of MSM who are HIV positive in Miami are Hispanic/Latino	◯	◯	◯	◯	◯

11. Please tell us more about why you rated these methods the way you did.

Size of HIV Positive MSM-PWID Population

On the next page, you will be asked to consider estimates for the population size of all HIV positive adult (15-64) men who have sex with men and inject drugs. This population includes both those who are unaware of their status <u>and</u> those who are diagnosed.

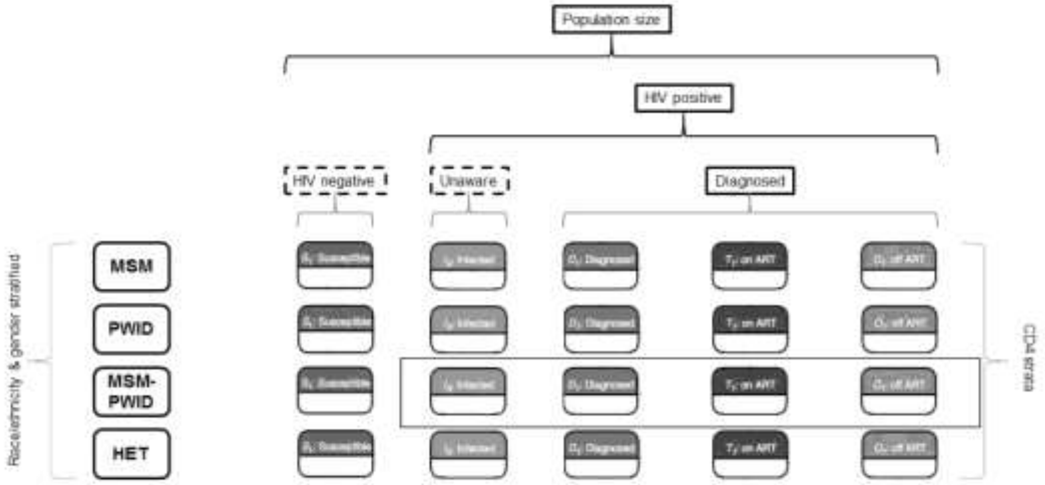

Size of HIV Positive MSM-PWID Population

To estimate the population size of HIV positive MSM-PWID in each city, we require race/ethnicity and risk group specific HIV prevalence estimates for MSM-PWID. We have not yet been able to locate such prevalence estimates.

12. Are you aware of any city-level data to estimate HIV prevalence among MSM-PWID in {{ Q1 }} ?

- ◯ No
- ◯ Yes (please specify)

13. Using a scale from 1 (not at all confident) to 5 (completely confident), please rate how confident you are in the following methods to estimate the proportion of HIV positive MSM-PWID by race/ethnicity in {{ Q1 }}.

	1 Not at all confident	2 Not very confident	3 Neutral	4 Somewhat confident	5 Completely confident
**1.Representative of city-level cumulative diagnoses of HIV** If 30% of Seattle's cumulative diagnoses of HIV among MSM-PWID are White, then 30% of MSM-PWID who are HIV positive are White.	◯	◯	◯	◯	◯
**2. Representative of city-level recent (last year) diagnoses of HIV** If 15% of Seattle's recent diagnoses of HIV among MSM-PWID are White, then 15% of MSM-PWID who are HIV positive are White.	◯	◯	◯	◯	◯

14. Please tell us more about why you rated these methods the way you did.

Size of HIV Positive PWID population

On the next page, you will be asked to consider estimates for the population size of all HIV positive adult (15-64) people who inject drugs. This population includes both those who are unaware of their status <u>and</u> those who are diagnosed.

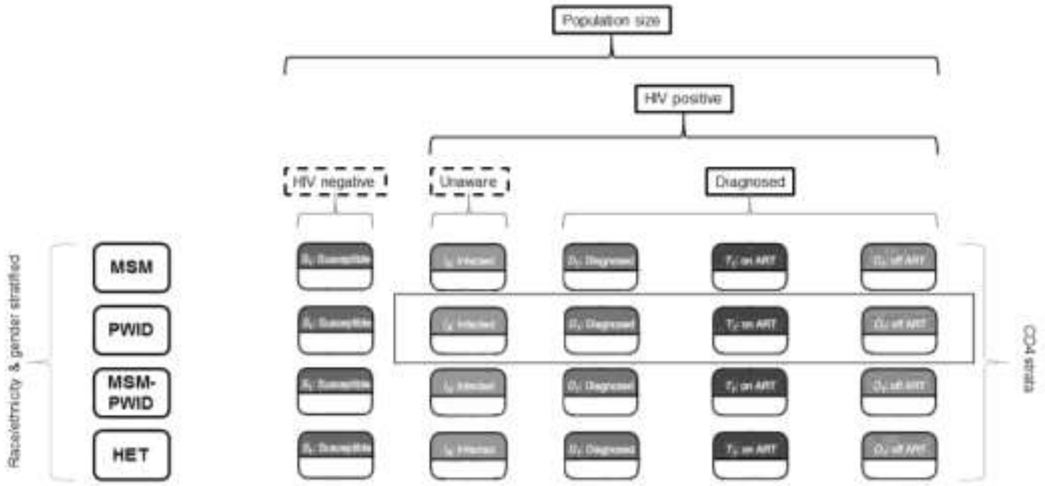

Localized Economic Modeling: Scientific Advisory Committee Survey

Size of HIV Positive PWID Population

To estimate the size of the HIV-infected PWID population in each city, we use 2002 estimates from Tempalski et al (2009) on HIV prevalence rates among PWID.

City	HIV Prevalence among PWID
Los Angeles, CA	3.8%
Miami, FL	22.8%
Atlanta, GA	14.9%
Baltimore, MD	11.7%
New York City, NY	11.0%
Seattle, WA	2.9%

Source: Tempalski B, Lieb S, Cleland C, Cooper H, Brady J, Friedman S. HIV prevalence rates among injection drug users in 96 large US metropolitan areas, 1992-2002. J Urban Health. 2009;86(1):132-54.

15. Are you aware of <u>more recent</u> data to estimate HIV prevalence among PWID in {{ Q1 }}?

- ◯ No
- ◯ Yes (please specify)

16. Using a scale from 1 (not at all confident) to 5 (completely confident), please rate how confident are you in the following methods to estimate the proportion of PWID who are HIV positive by race/ethnicity in {{ Q1 }}.

	1 Not at all confident	2 Not very confident	3 Neutral	4 Somewhat confident	5 Completely confident
1. **Equal proportion of PWID who are HIV positive across race/ethnicities** If 25% of PWID in Miami are estimated to be HIV positive, then 25% of Black PWID in Miami are HIV positive	○	○	○	○	○
**2. Representative of city-level cumulative diagnoses of HIV among PWID** If 30% of Miami's cumulative diagnoses of HIV among PWID are Black, then 30% of PWID who are HIV positive in Miami are Black.	○	○	○	○	○
**3. Representative of city-level recent (last year) diagnoses of HIV among PWID** If 20% of Miami's last-year diagnoses of HIV among PWID are Black, then 20% of PWID who are HIV positive in Miami are Black	○	○	○	○	○

17. Please tell us more about why you rated these methods the way you did.

Cross-tabulation of diagnosed PLHIV by race/ethnicity and transmission group

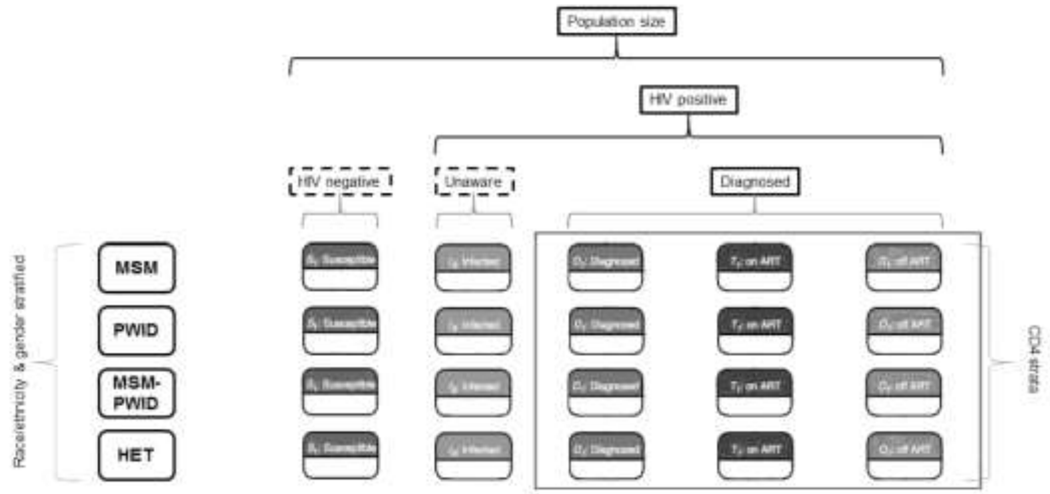

Cross-tabulation of PLHIV by race/ethnicity and transmission group

We have obtained the population size of diagnosed PLHIV, cross-tabulated by sex, race/ethnicity and risk group, from Los Angeles' and Seattle's HIV surveillance reports (see example below). However, most city-level reports do not provide cross-tabulation of race/ethnicity and risk group. Instead, they provide the distribution of diagnosed PLHIV by risk, and by race/ethnicity as separate data.

TABLE 3. CUMULATIVE HIV/AIDS DIAGNOSES AMONG ADULTS/ADOLESCENTS BY GENDER, TRANSMISSION CATEGORY, AND RACE/ETHNICITY
REPORTED BY DECEMBER 31, 2010
LOS ANGELES COUNTY

Adult/Adolescent Transmission Category (1)	Race/Ethnicity White No. (%)	Black No. (%)	Hispanic/Latino No. (%)	Asian/PI No. (%)	AI/AN (2) No. (%)	Total (3) No. (%)
MALE						
Male-male sexual contact	26054 (88)	9862 (77)	18960 (85)	1501 (91)	199 (76)	57142 (85)
Injection drug user (IDU)	918 (3)	1181 (9)	1180 (5)	26 (2)	15 (6)	3365 (5)
Male-male sexual contact/IDU	2371 (8)	1240 (10)	1390 (6)	55 (3)	44 (17)	5182 (8)
Hemophilia/coagulation disorder	91 (<1)	24 (<1)	39 (<1)	9 (1)	<5 (-)	164 (<1)
Heterosexual contact (4)	202 (1)	414 (3)	772 (3)	37 (2)	<5 (-)	1438 (2)
Transfusion recipient	124 (<1)	36 (<1)	68 (<1)	16 (1)	<5 (-)	245 (<1)
Other/Undetermined	<5 (-)	<5 (-)	<5 (-)	<5 (-)	<5 (-)	26 (<1)
Male subtotal	29760	12757	22409	1643	261	67562
[% of subtotal]	[44]	[19]	[33]	[2]	[<1]	[100]

18. Are you aware of any city-level data for diagnosed PLHIV that is cross-tabulated by race/ethnicity and risk group? (Please skip this question if answering for LA or Seattle)

- ◯ No
- ◯ Yes (please specify)

19. Using a scale from 1 (not at all confident) to 5 (completely confident), please rate how confident you are in the following strategies to distribute diagnosed PLHIV by race/ethnicity in {{ Q1 }}.

	1<br>Not at all confident	2<br>Not very confident	3<br>Neutral	4<br>Somewhat confident	5<br>Completely confident
**1. Proportional to city-level distribution of overall diagnosed PLHIV by race/ethnicity**<br>If 60% of Atlanta's diagnosed PLHIV are Black, then 60% of Atlanta's diagnosed MSM cases are Black.	○	○	○	○	○
**2. Weighting the distribution of race/ethnicity within risk groups using published evidence at the state or other city level**<br>If 40% of diagnosed MSM in Georgia are Black, then 40% of diagnosed MSM in Atlanta are Black	○	○	○	○	○

20. Please tell us more about why you rated these methods the way you did.

Distribution of "other" transmission categories to four transmission groups

New York City has a high proportion of diagnosed PLHIV whose risk group is not categorized as PWID, MSM, PWID-MSM, or HET. Instead, for 24% of males and 31% of females diagnosed and living with HIV, their transmission risk is defined as other or unknown.

21. Using a scale from 1 (not at all confident) to 5 (completely confident), please rate how confident you are in the following strategies to distribute other/unknown cases to the four transmission groups.

	1 Not at all confident	2 Not very confident	3 Neutral	4 Somewhat confident	5 Completely confident
**1. Redistribution using cumulative diagnoses proportions for risk group** If 24% of cumulative diagnosed cases are other/unknown and 47% are MSM, then 64% of diagnosed should be MSM after redistribution *[New % MSM = Old % MSM / (1-%other/unknown)]*	○	○	○	○	○
**2. Redistribution using past year diagnoses proportions for risk group** If 24% of recent diagnosed cases are other/unknown, and 50% are MSM - then 66% of diagnosed cases should be MSM after redistribution *[new % MSM = old % MSM /(1-% other/unknown)]*	○	○	○	○	○
**3. Redistribution proportional to general population** If 13% of men are MSM; and 47% of diagnosed males are MSM and 24% are other/unknown; then 50% of diagnosed men are MSM after redistribution *[new %MSM = old %MSM + %oth/unknown * %MSM among males]*	○	○	○	○	○

22. Please tell us more about why you rated these statements the way you did.

SECTION 2: FORCE OF INFECTION

The force of infection in our dynamic model captures the probability of HIV transmission via three modes given viral infectivity and HIV risk behaviors: heterosexual contact, homosexual contact and needle-sharing.

The following questions pertain to obtaining city-level estimates on (stratified by risk group, race/ethnicity and gender, where applicable):

1. **Sexual risk behaviour**
    - **Annual number of opposite sex partners**
    - **Annual number of same sex partners**
    - **Proportion of individuals who used condoms every time during heterosexual sex in the past year**
    - **Proportion of individuals who used condoms every time during homosexual sex in the past year**
2. **Injection risk behavior**
    - **Annual needle sharing probability**
3. **ART effectiveness/efficacy on HIV transmission by needle sharing**

Localized Economic Modeling: Scientific Advisory Committee Survey

Estimates for MSM Sexual Risk Behaviour

We aimed to estimate MSM sexual risk behaviours using primary analysis of city-specific data from National HIV Behavioral Surveillance for MSM (NHBS-MSM).

The NHBS-MSM survey used a venue-based sampling method. We are aware that venue-based samples might represent MSM with higher risk, as compared to the general MSM population. Both unweighted and recruitment-weighted estimates do not necessarily apply to MSM who never attend MSM-oriented social venues.

23. How representative do you think venue-based samples (i.e. NHBS) are of MSM in {{ Q1 }}?

- ◯ Represent more than half of MSM
- ◯ Represent about half of the MSM
- ◯ Represent less than half of the MSM

24. Please tell us more about why you selected the response you did.

25. Are you aware of any relevant literature or data source that could improve the estimates of MSM sexual risk behaviour in {{ Q1 }}?

- ◯ No
- ◯ Yes (please specify)

26. While NHBS samples may not be representative of the general MSM population, they may remain the best source to provide estimates.

Using a scale from 1 (not at all confident) to 5 (completely confident), please rate how confident you are that the true estimates of sexual risk behaviours among all MSM in {{ Q1 }} can be determined by the following methods.

	1 Not at all confident	2 Not very confident	3 Neutral	4 Somewhat confident	5 Completely confident
**1. Unweighted estimates based on the NHBS-MSM**	◯	◯	◯	◯	◯
**2. Recruitment-weighted (venue-based sampling weights) estimates based on NHBS-MSM**	◯	◯	◯	◯	◯
**3. Assumption-weighted estimate** If 60% of general MSM have the same mean number of partners as all NHBS-MSM (5); and the other 40% has a lower risk with a mean of 2 partners; then the estimates for the overall general MSM population is 3.8	◯	◯	◯	◯	◯

27. Please tell us more about why you rated the way you did

28. Are you aware of any data sources that could improve our estimates of MSM sexual risk behavior in {{ Q1 }}?

◯ No

◯ Yes (please specify)

Localized Economic Modeling: Scientific Advisory Committee Survey

Estimates for PWID Sexual Risk Behaviour and Needle Sharing

We aimed to estimate PWID sexual risk behavior and needle sharing behavior using primary analysis of data from National HIV Behavioral Surveillance for PWID (NHBS-IDU) (city-specific). The NHBS-IDU used respondent-driven sampling method.

Alternatively, the estimates for Baltimore can also be obtained from the ALIVE cohort study.

29. Using a scale of 1 (not at all confident) to 5 (completely confident), please rate how confident you are that the true estimates of sexual risk behaviors and needle sharing among all PWID can be determined by the following methods.

	1 Not at all confident	2 Not very confident	3 Neutral	4 Somewhat confident	5 Completely confident
**1. Unweighted estimates based on the NHBS-IDU**	○	○	○	○	○
**2. Recruitment-weighted (respondent-driven sampling weights) estimates based on the NHBS-IDU**	○	○	○	○	○
**3. For Baltimore only: Estimates based on race/ethnicity specific estimates from the ALIVE cohort**	○	○	○	○	○

30. Please tell us more about why you rated the way you did.

31. Are you aware of any data sources that could improve estimates for PWIDsexual risk behaviour in {{ Q1 }}?

- ◯ No
- ◯ Yes (please specify)

32. Are you aware of any data sources that could improve estimates for PWIDneedle sharing in {{ Q1 }}?

- ◯ No
- ◯ Yes (please specify)

Estimates for HET Sexual Risk Behaviour

We estimated heterosexual risk behaviour among adults (15-44) using the National Survey of Family Growth (NSFG), a household based stratified multi-stage area probability sample of the US general population. Region-specific (northeast, midwest, south and west) estimates were obtained from the NSFG 2011-13 data files (below).

We are not using the National HIV Behavioural Surveillance for heterosexuals because the study sample represents HET of high risk and low socio-economic status.

Table. Sexual risk behaviors among heterosexual* participants in the NSFG, 2011-2013

		Race/Ethnicity		
		White	Black	Hispanic
Gender	Region	**Annual average number of opposite sex partners (95% CI)**		
Male	Northeast	1.32 (1.10 - 1.55)	2.35 (1.44 - 3.26)	2.00 (1.30 - 2.71)
	Midwest	1.38 (1.19 - 1.56)	1.76 (1.59 - 1.93)	1.21 (0.87 - 1.54)
	South	1.37 (1.19 - 1.55)	1.92 (1.74 - 2.10)	1.30 (1.13 - 1.46)
	West	1.15 (1.04 - 1.26)	1.63 (0.91 - 2.34)	1.35 (1.15 - 1.55)
Female	Northeast	1.14 (1.07 - 1.21)	1.55 (1.35 - 1.75)	1.67 (0.91 - 2.43)
	Midwest	1.24 (1.08 - 1.39)	1.47 (1.22 - 1.73)	1.16 (0.91 - 1.42)
	South	1.17 (1.09 - 1.25)	1.24 (1.15 - 1.33)	1.17 (1.06 - 1.27)
	West	1.21 (1.11 - 1.30)	1.14 (1.00 - 1.28)	1.11 (1.03 - 1.18)
		**Proption of individuals who used condoms every time in the past year (95% CI)**		
Male	Northeast	21.39 (15.28 - 27.49)	22.91 (13.98 - 31.83)	26.08 (16.67 - 35.49)
	Midwest	23.03 (15.64 - 30.42)	27.29 (21.41 - 33.18)	33.08 (16.21 - 49.96)
	South	15.08 (11.85 - 18.32)	20.66 (15.97 - 25.36)	18.38 (10.27 - 26.49)
	West	16.64 (10.84 - 22.44)	14.48 (0.00 - 32.92)	21.11 (15.59 - 26.63)
Female	Northeast	15.50 (11.45 - 19.55)	35.72 (30.41 - 41.03)	15.05 (9.31 - 20.80)
	Midwest	12.82 (8.46 - 17.19)	17.74 (9.56 - 25.91)	14.25 (7.47 - 21.03)
	South	11.02 (7.45 - 14.60)	20.59 (14.77 - 26.42)	15.37 (9.49 - 21.25)
	West	9.98 (7.38 - 12.58)	20.88 (6.47 - 35.30)	15.83 (10.09 - 21.56)

Source: the national survey of family grow th (NSFG) 2011-2013 public use data files and REGION data file (Age 15-44 years).
Methods: w eighted estimates accounting for the multi-stage stratified sampling design.
*Heteroseuxals defined by individuals' self-reported sexual orientation.

33. Using a scale from 1 (not at all confident) to 5 (completely confident), please rate how confident you are that the region-specific estimates based on NSFG represent true estimates of the sexual risk behaviors among all HETs in {{ Q1 }}.

- ◯ **1**- Not at all confident
- ◯ **2**- Not very confident
- ◯ **3**- Neutral
- ◯ **4**- Somewhat confident
- ◯ **5**- Completely confident

34. Please tell us more about why you rated your confidence the way you did.

35. Are you aware of any data sources that could improve our estimates of heterosexual sexual risk in {{ Q1 }}?

- No
- Yes (please specify)

Localized Economic Modeling: Scientific Advisory Committee Survey

Estimates for MSM-PWID HIV Risk Behaviour

We aimed to obtain estimates for MSM-PWID sexual risk behaviour based on NHBS-MSM and NHBS-IDU surveys. However, there may be an insufficient sample size to estimate city, race/ethnicity, and gender-specific estimates for MSM-PWID.

36. Using a scale from 1 (not at all confident) to 5 (completely confident), please rate how confident you are that the true estimates of sexual risk behaviours for MSM-PWID in {{ Q1 }} can be determined by the following methods.

	1 Not at all confident	2 Not very confident	3 Neutral	4 Somewhat confident	5 Completely confident
**1. Assume the sexual risk behaviours of MSM-PWID to be the same across six cities** (six city combined estimates)	○	○	○	○	○
**2. Assume the sexual risk behaviors of MSM-PWID to be the same as MSM** (e.g., if the average number of same sex partners is 5 for White MSM in your city, then assume it is 5 for White MSM-PWID)	○	○	○	○	○

37. Please tell us more about why you rated these methods the way you did

38. Are you aware of any data sources that could improve our estimates of MSM-PWID sexual risk behaviour in {{ Q1 }}?

- ○ No
- ○ Yes (please specify)

Estimates for MSM-PWID Needle Sharing Behaviour

We aimed to obtain estimates for MSM-PWID needle sharing probability based on NHBS-MSM and NHBS-IDU surveys. However, there may be an insufficient sample size to estimate city-, race/ethnicity, and gender-specific estimates for MSM-PWID.

39. Using a scale from 1(not at all confident) to 5 (completely confident), please rate how confident you are that the true estimates of needle sharing probability for MSM-PWID in {{ Q1 }} can be determined by the following methods.

	1 Not at all confident	2 Not very confident	3 Neutral	4 Somewhat confident	5 Completely confident
**1. Assume the needle sharing probability of MSM-PWID to be the same across six cities** (six city combined estimates)	◯	◯	◯	◯	◯
**2. Assume the needle sharing probability of MSM-PWID to be the same as PWID** (e.g., if the needle sharing probability is 25% for White PWID in your city, then assume it is 25% for White MSM-PWID)	◯	◯	◯	◯	◯

40. Please tell us more about why you rated the way you did.

41. Are you aware of any data source that could improve the estimates of MSM-PWID needle sharing probability in {{ Q1 }}?

- ◯ No
- ◯ Yes (please specify)

Localized Economic Modeling: Scientific Advisory Committee Survey

ART effectiveness on HIV transmission by needle sharing

We have found limited evidence on ART effectiveness on HIV transmission by needle sharing. Other modelling studies have used point (range) estimates of 50% (10%-90%). [1,2]

1. Long EF, Brandeau ML, Owens DK. The cost-effectiveness and population outcomes of expanded HIV screening and antiretroviral treatment in the United States. Annals of internal medicine. 2010 Dec 21;153(12):778-89.

2. Nosyk B, Zang X, Min JE, Krebs E, Lima VD, Milloy MJ, Shoveller J, Barrios R, Harrigan PR, Kerr T, Wood E. Relative effects of antiretroviral therapy and harm reduction initiatives on HIV incidence in British Columbia, Canada, 1996–2013: a modelling study. The Lancet HIV. 2017 Mar 30.

42. Using a scale from 1 (not at all confident) to 5 (completely confident), please rate how confident you are about these estimates for ART effectiveness on HIV transmission by needle sharing?

- **1** Not at all Confident
- **2** Not very confident
- **3** Neutral
- **4** Somewhat confident
- **5** Completely confident

43. Please tell us more about why you rated your confidence the way you did.

44. Are you aware of any other evidence on ART effectiveness on HIV transmission by needle sharing?

- No
- Yes (please specify)

SECTION 3: ART ENGAGEMENT AND DISEASE PROGRESSION

The following questions pertain to obtaining city-level estimates on (stratified by risk group, race/ethnicity, gender and CD4, where applicable):

1. **ART interruption probability**
2. **Time from diagnosis to ART initiation**

Localized Economic Modeling: Scientific Advisory Committee Survey

Estimates on Annual ART interruption probability

We've estimated ART interruption probability using a multi-state Markov model, based on data from the HIV Research Network (HIVRN). The network of HIV care providers is a consortium of adult and pediatric clinics in which some are hospital-based with a university affiliation, others are hospital-based not affiliated with a university, and others are community-based providers.

Region indicators of the participating clinics were available in the dataset, including Northeast (Rochester, Boston, New York City, Baltimore, and Philadelphia), South (Dallas, Memphis, Tampa) and West (Portland, Oakland, and San Diego).

Table. Annual probability of ART interruption among HIVRN participants with a **CD4<200***, 2007-2015

		Race/Ethnicity		
		White	Black	Hispanic
Region	Risk group	**Annual probability of ART interruption (95% CI)**		
Northeast	MSM	0.14 (0.12 - 0.18)	0.19 (0.17 - 0.22)	0.19 (0.16 - 0.23)
Northeast	PWID*	0.19 (0.15 - 0.24)	0.25 (0.21 - 0.30)	0.26 (0.21 - 0.32)
Northeast	HET_Female	0.14 (0.11 - 0.19)	0.19 (0.16 - 0.22)	0.19 (0.16 - 0.23)
Northeast	HET_Male	0.16 (0.13 - 0.20)	0.21 (0.18 - 0.25)	0.21 (0.18 - 0.26)
South	MSM	0.22 (0.19 - 0.25)	0.25 (0.22 - 0.28)	0.16 (0.14 - 0.18)
South	PWID*	0.31 (0.26 - 0.37)	0.33 (0.30 - 0.38)	0.22 (0.18 - 0.26)
South	HET_Female	0.21 (0.17 - 0.25)	0.23 (0.21 - 0.26)	0.15 (0.12 - 0.18)
South	HET_Male	0.23 (0.20 - 0.28)	0.26 (0.23 - 0.28)	0.16 (0.14 - 0.19)
West	MSM	0.21 (0.18 - 0.25)	0.28 (0.23 - 0.34)	0.19 (0.16 - 0.23)
West	PWID*	0.26 (0.20 - 0.33)	0.34 (0.26 - 0.44)	0.24 (0.18 - 0.31)
West	HET_Female	0.19 (0.14 - 0.26)	0.26 (0.19 - 0.34)	0.17 (0.13 - 0.25)

Source: participants enrolled in HIV Research Netw ork participating clinics betw een 2007-2015.
Methods: region-stratified multivariable multi-state Markov models, adjusting for race/ethnicity and risk goup
* ART interruption probability from a CD4<200 w as presented as an example.
* PWID-Female, PWID-Male, and MSM-PWID w ere grouped together as w e did not have pow er to detect any statistically-significant difference.

45. Using a scale from 1 (not at all confident) to 5 (completely confident), please rate how confident you are that the true probability of ART interruption in {{ Q1 }} can be determined by the region-specific estimates shown in the table above.

- ○ **1** Not at all confident
- ○ **2** Not very confident
- ○ **3** Neutral
- ○ **4** Somewhat confident
- ○ **5** Completely confident

46. Please tell us more about why you rated your confidence the way you did.

47. Are you aware of other data source that could improve these estimates of ART interruption probability?

- No
- Yes (please specify)

Localized Economic Modeling: Scientific Advisory Committee Survey

Time to initiate ART

48. Are you aware of any data to determine time-to-ART initiation following diagnosis in {{ Q1 }} (ex. longitudinal viral load data)?

- ◯ No
- ◯ Yes (please specify)

Localized Economic Modeling: Scientific Advisory Committee Survey

DOMAIN 4: Health System Engagement

In this section, we will ask about parameters related to health system engagement of susceptibles and PLHIV. Specifically, we will be asking about:

1. HIV testing
2. Syringe Distribution
3. Opioid Agonist Treatment (OAT) coverage
4. Buprenorphine (BUP) Utilization
5. Pre-exposure prophylaxis(PrEP)

Localized Economic Modeling: Scientific Advisory Committee Survey

HIV Testing

We are striving to obtain city-level HIV testing events data for 2010 & 2014. In particular, we need the yearly number of unique test events for each risk/gender/ethnicity where multiple tests on the same day for an individual are counted as one testing event (as per CDC reporting).

Our best source of evidence for testing events are:

Atlanta: aggregate city-level testing events data from CDC (2014 only; state-level for 2010).

Baltimore: aggregate city-level testing events data from CDC (2014 only; state-level for 2010).

Los Angeles: site-specific testing data with distribution stratified by gender/ethnicity and risk (2010; 2014 city-level aggregate data with gender/ethnicity distribution only).

Miami: state-level risk/ethnicity stratified testing distribution & city-level gender/ethnicity testing events data with atypical risk categorization.

New York City: aggregate city-level testing events data from CDC.

Seattle: aggregate state-level testing events data from CDC and King county surveillance testing events data for MSM and totals (derived from chart in surveillance report).

49. Are you aware of better HIV testing data (stratified by risk, gender and race/ethnicity) available for {{ Q1 }}?

- ◯ No
- ◯ Yes (please specify)

50. Using a scale from 1 (not at all confident) to 5 (completely confident), please rate how confident you are that the true distribution of testing event numbers for each risk, gender, and race/ethnicity strata for a given aggregate city-level number of yearly testing events can be determined by the following assumptions.

	1<br>Not at all confident	2<br>Not very confident	3<br>Neutral	4<br>Somewhat confident	5<br>Completely confident
**1. Assume testing events are proportional to past year diagnoses**<br>If Hispanic/Latino heterosexual women represent 1.25% of new HIV diagnoses, then 1.25% of city-level testing events will be attributed to Hispanic/Latino heterosexual women					
**2. Assume testing events are proportional to cumulative diagnoses**<br>If Hispanic/Latino HET women represent 0.25% of PLHIV (i.e., cumulative HIV diagnoses), then 0.25% of city-level testing events will be attributed to Hispanic/Latino HET women					
**3. Assume testing events are proportional to susceptible population sizes**<br>If Hispanic/Latino HET women represent 4.5% of the susceptible population, then 4.5% of city-level testing events will be attributed to hispanic/latino HET women					

	1<br>Not at all confident	2<br>Not very confident	3<br>Neutral	4<br>Somewhat confident	5<br>Completely confident
**4. Assume testing events are proportional to a susceptible-population-weighted probability of being tested derived from NHBS or BFRSS of reporting a HIV test receipt in the last 12 months**<br>Implies past year strata-specific rates are representative	◯	◯	◯	◯	◯

51. Please tell us more about why you rated these methods the way you did

Localized Economic Modeling: Scientific Advisory Committee Survey

Syringe Distribution

We are striving to obtain city-level number of syringes distributed by syringe services programs (SSP), ideally stratified by race/ethnicity or gender. Our best estimates for syringe distribution are:

Atlanta: We are using national median number of syringes distributed by SSP for each city-level SSP site as reported by North American Syringe Exchange Network (NASEN)

Baltimore: We are waiting to hear back from the Program Director of Community Risk Reduction Services at the Baltimore City Health Department about our request for more specific data.

Los Angeles: We have obtained stratified data from the City of Los Angeles.

Miami: We are waiting to hear back from Dan Feaster who is reaching out on our behalf to SSPs operating in Miami.

New York City: We have aggregate state-level syringe distribution data for 2006 & 2012. We are assuming city-level distribution numbers equivalent to the city-to-state proportion of SSPs. We have contacted the Injection Drug Users Health Alliance, but have yet to receive a response.

Seattle: We have obtained stratified data from King County.

52. Using a scale from 1 (not at all confident) to 5 (completely confident), please rate how confident you are that the true distribution of syringes for each risk, gender, and race/ethnicity strata for a given aggregate city-level number of syringes can be determined by the following assumptions.

	1 Not at all confident	2 Not very confident	3 Netural	4 Somewhat confident	5 Completely confident
**1. Identical to national gender and race/ethnicity weighted syringe distribution numbers obtained from the Dave Purchase Memorial National Survey of Syringe Exchange Programs and reported by NASEN**	○	○	○	○	○
**2. Identical city-level average coverage for each risk, gender and race/ethnicity strata**	○	○	○	○	○

53. Please tell us more about why you rated these statements the way you did.

54. Are you aware of a person or organization that we could contact for syringe distribution numbers in your city?

- No
- Yes (please specify)

Localized Economic Modeling: Scientific Advisory Committee Survey

Opioid Agonist Treatment coverage

To obtain city-level number of individuals receiving opioid agonist treatment (OAT), we are using gender and race/ethnicity stratified state-level number of opioid treatment program (OTP) clients receiving OAT that report any use of opioids AND injection as route of administration (from Treatment Episodes Dataset) weighted by the city-to-state proportion of OTP facilities (from N-SSATS 2010 & 2014 directories).

Example: If there are 5,000 white males in California reporting OAT receipt in OTPs and injection as route of administration (in TEDS), and if 30% of California OTPs are in Los Angeles, then we will assume that 1,500 white males are receiving OAT (1500*0.30).

OTP facilities from N-SSATS directory				
	2010		2014	
	OTP	OTP (%)	OTP	OTP (%)
ATL	16	43.2%	23	57.5%
BAL	43	74.1%	41	70.7%
LA	42	32.6%	39	35.1%
MIA	8	24.2%	10	27.0%
NYC	104	83.9%	88	86.3%
SEA	12	63.2%	12	57.1%

OAT clients from TEDS; OTP facilities from N-SSATS directory								
2010		Total^	(M)			(F)		
			W/O	B/AA	H/L	W/O	B/AA	H/L
CA		21,066						
	LA	4,588	1,632	267	1,240	882	154	413
FL		6,236						
	MIA	395	154	3	16	212	1	9
GA		6,808						
	ATL	2,944				N/A		
MD		5,421						
	BAL	2,190	952	267	22	787	149	13
NY		11,748						
	NYC	4,704	1,421	377	1,567	731	179	429
WA		1,957						
	SEA	846	379	35	23	357	25	27

2014		Total	(M)			(F)		
			W/O	B/AA	H/L	W/O	B/AA	H/L
CA		29,090						
	LA	6,595	2,412	330	1,783	1,333	157	580
FL		916						
	MIA	142	58	1	5	74	-	4
GA		10,193						
	ATL	5,861				N/A		
MD		6,027						
	BAL	2,258	939	306	54	768	165	26
NY		15,988						
	NYC	6,985	2,663	346	1,795	1,577	167	437
WA		3,600						
	SEA	1,454	695	51	41	600	36	31

*Stratified OAT clients data is not available for Atlanta as only state-level aggregate number of OAT clients is available.

55. Are you aware of better data sources for OAT coverage, stratified by race/ethnicity in {{ Q1 }}?

○ No

Yes (please specify)

56. Using a scale of 1 (not at all confident) to 5 (completely confident), please rate how confident you are in the following methods used to distribute the proportion of OAT clients by gender within each race/ethnicity in {{ Q1 }}.

	1 Not at all confident	2 Not very confident	3 Neutral	4 Somewhat confident	5 Completely confident
**1 Assume distribution is representative of city-level gender distribution of the general population** If 50% of white adults in Los Angeles are male, then 50% of the white OAT clients in Atlanta will be male	◯	◯	◯	◯	◯
**2. Assume distribution is representative of national-level gender distribution of OAT clients** If 70% of White OAT clients in the US are male, then 70% of the white OAT population in Atlanta will be male	◯	◯	◯	◯	◯

Buprenorphine Utilization

We want to incorporate city-specific evidence of buprenorphine (BUP, including BUP/Naloxone) utilization among PWID.

57. Using a scale from 1 (not at all confident) to 5 (completely confident), please rate how confident you are that the true number of PWID receiving OAT with BUP can be determined by the following assumptions, given further weighting by city-to-state proportion of substance abuse treatment (SAT) facilities offering BUP (from N-SSATS 2010 & 2014 directories).

	1 Not at all confident	2 Not very confident	3 Neutral	4 Somewhat confident	5 Completely confident
**1. The number of PWID receiving BUP is determined to be 25% of state-level reported maximum physician prescribing capacity, based on evidence of the proportion of heroin users in a national survey of BUP prescription recipients.**	◯	◯	◯	◯	◯
**2. The number of PWID receiving BUP is determined to be 10% of state-level DATA-reported maximum physician prescribing capacity, based on evidence of the proportion of DATA-waivered physicians in NYC that acccept Medicaid.**	◯	◯	◯	◯	◯
**3. The number of PWID receiving BUP is determined to be equivalent to the number of quarterly prescriptions from state Medicaid data of drugs containing BUP in the non-proprietary drug name.**	◯	◯	◯	◯	◯

58. Please tell us more about why you answered the way you did.

59. Are you aware of better data sources to estimate BUP utilization rates among PWID in {{ Q1 }}?

- ◯ No
- ◯ Yes (please specify)

Localized Economic Modeling: Scientific Advisory Committee Survey

Pre-exposure prophylaxis (PrEP)

We will obtain stratified city-level uptake among adults (15-64) of PrEP directly from primary analysis of NHBS data.

60. Are you aware of better data sources for PrEP uptake among MSM in {{ Q1 }} for the year 2014?

- ( ) No
- ( ) Yes (Please specify)
